# Immunological and molecular features of the tumor microenvironment of long-term survivors of ovarian cancer

**DOI:** 10.1172/JCI179501

**Published:** 2024-10-29

**Authors:** Brad H. Nelson, Phineas Hamilton, Minh Tung Phung, Katy Milne, Bronwyn Harris, Shelby Thornton, Donald Stevens, Shreena Kalaria, Karanvir Singh, Céline M. Laumont, Elena Moss, Aliya Alimujiang, Nicola S. Meagher, Adelyn Bolithon, Sian Fereday, Catherine J. Kennedy, Joy Hendley, Dinuka Ariyaratne, Kathryn Alsop, Nadia Traficante, Ellen L. Goode, Anthony Karnezis, Hui Shen, Jean Richardson, Cindy McKinnonDeurloo, Anne Chase, Bronwyn Grout, Jennifer Anne Doherty, Holly R. Harris, Kara L. Cushing-Haugen, Michael Anglesio, Karolin Heinze, David Huntsman, Aline Talhouk, Gillian E. Hanley, Jennifer Alsop, Mercedes Jimenez-Linan, Paul D.P. Pharoah, Jessica Boros, Alison H. Brand, Paul R. Harnett, Raghwa Sharma, Jonathan L. Hecht, Naoko Sasamoto, Kathryn L. Terry, Beth Karlan, Jenny Lester, Michael E. Carney, Marc T. Goodman, Brenda Y. Hernandez, Lynne R. Wilkens, Sabine Behrens, Renée Turzanski Fortner, Peter A. Fasching, Christiani Bisinotto, Francisco José Candido dos Reis, Prafull Ghatage, Martin Köbel, Esther Elishaev, Francesmary Modugno, Linda Cook, Nhu Le, Aleksandra Gentry-Maharaj, Usha Menon, María J. García, Cristina Rodriguez-Antona, Kyo Farrington, Linda E. Kelemen, Stefan Kommoss, Annette Staebler, Dale W. Garsed, James D. Brenton, Anna M. Piskorz, David D.L. Bowtell, Anna DeFazio, Susan J. Ramus, Malcolm C. Pike, Celeste Leigh Pearce

**Affiliations:** 1 Deeley Research Centre, BC Cancer, Victoria, British Columbia, Canada.; 2 Department of Biochemistry and Microbiology, University of Victoria, Victoria, British Columbia, Canada.; 3 Department of Medical Genetics, University of British Columbia, Vancouver, British Columbia, Canada.; 4 School of Public Health, University of Michigan, Ann Arbor, Michigan, USA.; 5 Department of Population Health Sciences, School of Medicine and Public Health, University of Wisconsin-Madison, Madison, Wisconsin, USA.; 6 School of Clinical Medicine, University of New South Wales (NSW) Medicine and Health, University of NSW Sydney, Sydney, New South Wales, Australia.; 7 The Daffodil Centre, The University of Sydney, a joint venture with Cancer Council NSW, Sydney, New South Wales, Australia.; 8 Adult Cancer Program, Lowy Cancer Research Centre, University of NSW Sydney, Sydney, New South Wales, Australia.; 9 Peter MacCallum Cancer Centre, Melbourne, Victoria, Australia.; 10 Sir Peter MacCallum Department of Oncology, The University of Melbourne, Parkville, Victoria, Australia.; 11 Centre for Cancer Research, The Westmead Institute for Medical Research, Sydney, New South Wales, Australia.; 12 Department of Gynaecological Oncology, Westmead Hospital, Sydney, New South Wales, Australia.; 13 Faculty of Medicine and Health, The University of Sydney, Sydney, New South Wales, Australia.; 14 Division of Epidemiology, Department of Quantitative Health Sciences, Mayo Clinic, Rochester, Minnesota, USA.; 15 Department of Pathology, University of California Davis School of Medicine, Sacramento, California, USA.; 16 Van Andel Institute, Grand Rapids, Michigan, USA.; 17 Department of Population and Public Health Sciences, Keck School of Medicine, University of Southern California, Los Angeles, California, USA.; 18 Patient advocate.; 19 Huntsman Cancer Institute, Department of Population Health Sciences, University of Utah, Salt Lake City, Utah, USA.; 20 Program in Epidemiology, Division of Public Health Sciences, Fred Hutchinson Cancer Center, Seattle, Washington, USA.; 21 Department of Epidemiology, University of Washington, Seattle, Washington, USA.; 22 Department of Obstetrics and Gynecology, University of British Columbia, Vancouver, British Columbia, Canada.; 23 British Columbia’s Gynecological Cancer Research Team (OVCARE), University of British Columbia, BC Cancer, and Vancouver General Hospital, Vancouver, British Columbia, Canada.; 24 Department of Molecular Oncology, BC Cancer Research Centre, Vancouver, British Columbia, Canada.; 25 Centre for Cancer Genetic Epidemiology, Department of Oncology, University of Cambridge, Cambridge, United Kingdom.; 26 Department of Histopathology, Addenbrooke’s Hospital, Cambridge, United Kingdom.; 27 Department of Computational Biomedicine, Cedars-Sinai Medical Center, West Hollywood, California, USA.; 28 Crown Princess Mary Cancer Centre and; 29 Tissue Pathology and Diagnostic Oncology, NSW Health Pathology, Westmead Hospital, Sydney, New South Wales, Australia.; 30 Western Sydney University, Sydney, New South Wales, Australia.; 31 Department of Pathology, Beth Israel Deaconess Medical Center and Harvard Medical School, Boston, Massachusetts, USA.; 32 Obstetrics and Gynecology Epidemiology Center, Department of Obstetrics and Gynecology, Brigham and Women’s Hospital and Harvard Medical School, Boston, Massachusetts, USA.; 33 Department of Epidemiology, Harvard T.H. Chan School of Public Health, Boston, Massachusetts, USA.; 34 David Geffen School of Medicine, Department of Obstetrics and Gynecology, University of California at Los Angeles, Los Angeles, California, USA.; 35 Department of Obstetrics and Gynecology, John A. Burns School of Medicine University of Hawaii, Honolulu, Hawaii, USA.; 36 Cancer Prevention and Control Program, Cedars-Sinai Cancer Center, Cedars-Sinai Medical Center, Los Angeles, California, USA.; 37 University of Hawaii Cancer Center, Honolulu, Hawaii, USA.; 38 Division of Cancer Epidemiology, German Cancer Research Center (DKFZ), Heidelberg, Germany.; 39 Department of Research, Cancer Registry of Norway, Oslo, Norway.; 40 Department of Gynecology and Obstetrics, Comprehensive Cancer Center Erlangen-EMN, Friedrich-Alexander University Erlangen-Nuremberg, University Hospital Erlangen, Erlangen, Germany.; 41 Department of Gynecology and Obstetrics, Ribeirão Preto Medical School, University of São Paulo, Ribeirão Preto, Brazil.; 42 Department of Oncology, Division of Gynecologic Oncology, Cumming School of Medicine, University of Calgary, Calgary, Alberta, Canada.; 43 Department of Pathology and Laboratory Medicine, University of Calgary, Foothills Medical Center, Calgary, Alberta, Canada.; 44 Department of Pathology, University of Pittsburgh School of Medicine, Pittsburgh, Pennsylvania, USA.; 45 Department of Epidemiology, University of Pittsburgh School of Public Health, Pittsburgh, Pennsylvania, USA.; 46 Division of Gynecologic Oncology, Department of Obstetrics, Gynecology and Reproductive Sciences, University of Pittsburgh School of Medicine, Pittsburgh, Pennsylvania, USA.; 47 Women’s Cancer Research Center, Magee-Womens Research Institute and Hillman Cancer Center, Pittsburgh, Pennsylvania, USA.; 48 Epidemiology, School of Public Health, University of Colorado, Aurora, Colorado, USA.; 49 Community Health Sciences, University of Calgary, Calgary, Alberta, Canada.; 50 Cancer Control Research, BC Cancer Agency, Vancouver, British Columbia, Canada.; 51 MRC Clinical Trials Unit, Institute of Clinical Trials and Methodology and; 52 Department of Women’s Cancer, Elizabeth Garrett Anderson Institute for Women’s Health, University College London, London, United Kingdom.; 53 Cancer Biology Department, Sols-Morreale Biomedical Research Institute (IIBM), CSIC UAM, Madrid, Spain.; 54 Hereditary Endocrine Cancer Group, Spanish National Cancer Research Center (CNIO), Madrid, Spain.; 55 Centre for Biomedical Network Research on Rare Diseases (CIBERER), Instituto de Salud Carlos III, Madrid, Spain.; 56 Division of Acute Disease Epidemiology, South Carolina Department of Health and Environmental Control, Columbia, South Carolina, USA.; 57 Department of Women’s Health and; 58 Institute of Pathology and Neuropathology, Tuebingen University Hospital, Tuebingen, Germany.; 59 Cancer Research UK Cambridge Institute, University of Cambridge, Cambridge, United Kingdom.; 60 Department of Epidemiology and Biostatistics, Memorial Sloan Kettering Cancer Center, New York, New York, USA.

**Keywords:** Immunology, Oncology, Cancer, Cellular immune response, Epidemiology

## Abstract

**BACKGROUND:**

Despite an overall poor prognosis, about 15% of patients with advanced-stage tubo-ovarian high-grade serous carcinoma (HGSC) survive 10 or more years after standard treatment.

**METHODS:**

We evaluated the tumor microenvironment of this exceptional, understudied group using a large international cohort enriched for long-term survivors (LTS; 10+ years; *n* = 374) compared with mid-term (MTS; 5–7.99 years; *n* = 433) and short-term survivors (STS; 2–4.99 years; *n* = 416). Primary tumor samples were immunostained and scored for intraepithelial and intrastromal densities of 10 immune-cell subsets (including T cells, B cells, plasma cells, myeloid cells, PD-1^+^ cells, and PD-L1^+^ cells) and epithelial content.

**RESULTS:**

Positive associations with LTS compared with STS were seen for 9 of 10 immune-cell subsets. In particular, the combination of intraepithelial CD8^+^ T cells and intrastromal B cells showed near 5-fold increased odds of LTS compared with STS. All of these associations were stronger in tumors with high epithelial content and/or the C4/Differentiated molecular subtype, despite immune-cell densities generally being higher in tumors with low epithelial content and/or the C2/Immunoreactive molecular subtype.

**CONCLUSION:**

The tumor microenvironment of HGSC LTS is distinguished by the intersection of T and B cell coinfiltration, high epithelial content, and C4/differentiated molecular subtype, features which may inspire new approaches to immunotherapy.

**FUNDING:**

Ovarian Cancer Research Program (OCRP) of the Congressionally Directed Medical Research Program (CDMRP), U.S. Department of Defense (DOD); American Cancer Society; BC Cancer Foundation; Canada’s Networks of Centres of Excellence; Canadian Cancer Society; Canadian Institutes of Health Research; Cancer Councils of New South Wales, Victoria, Queensland, South Australia, and Tasmania, Cancer Foundation of Western Australia; Cancer Institute NSW; Cancer Research UK; Deutsche Forschungsgesellschaft; ELAN Funds of the University of Erlangen-Nuremberg; Fred C. and Katherine B. Andersen Foundation; Genome BC; German Cancer Research Center; German Federal Ministry of Education and Research, Programme of Clinical Biomedical Research; Instituto de Salud Carlos III; Mayo Foundation; Minnesota Ovarian Cancer Alliance; Ministerio de Economía y Competitividad; Medical Research Council (MRC); National Center for Advancing Translational Sciences; National Health and Medical Research Council of Australia (NHMRC); Ovarian Cancer Australia; Peter MacCallum Foundation; Sydney West Translational Cancer Research Centre; Terry Fox Research Institute; The Eve Appeal (The Oak Foundation); UK National Institute for Health Research Biomedical Research Centres at the University of Cambridge; University of Pittsburgh School of Medicine; U.S. National Cancer Institute of the National Institutes of Health; VGH & UBC Hospital Foundation; Victorian Cancer Agency.

## Abstract 2

Ovarian cancers associated with exceptionally long-term survival show unexpectedly modest immune responses combined with more dense, differentiated tumor epithelium.

## Introduction

Although advanced-stage tubo-ovarian high-grade serous carcinoma (HGSC) remains a challenging, largely incurable disease, about 15% of patients survive 10 or more years after diagnosis (1), providing an opportunity to define the features associated with long-term survival. Progress on this front has been hampered by the relative rarity of such cases combined with the need for large long-term research programs with biospecimen banking and systematic clinical follow-up to accrue sufficient cases (2). For example, in the HGSC patient cohort of The Cancer Genome Atlas (TCGA), only 1% (4 of 405) of cases had an overall survival (OS) of 10+ years (3). To address these challenges, the Multidisciplinary Ovarian Cancer Outcomes Group (MOCOG) was formed to identify the immunologic, genomic, and epidemiological factors associated with long-term survival in HGSC. We report here our findings regarding the immune tumor microenvironment (TME) of 374 long-term survivors (LTS; 10+ years) compared with 433 mid-term survivors (MTS; 5–7.99 years), and 416 short-term survivors (STS; 2–4.99 years). Unusually poor survivors (< 2 years) were not included in the study because these patients may have had primary platinum-resistant disease or exceptionally late diagnosis.

The presence at diagnosis of tumor-infiltrating lymphocytes (TILs), in particular CD8^+^ T cells, is associated with improved survival from HGSC (4–7). Other TIL subsets associated with favorable prognosis include CD4^+^ T cells, CD20^+^ B cells, and plasma cells (7–14). Expression of PD-1 by TILs and expression of its ligand (PD-L1) by tumor and myeloid cells (15–17) is also a favorable prognostic feature in HGSC, likely reflecting the role of this pathway in active antitumor immunity (18). However, the immune cell composition associated with exceptional patient survival has not been defined and represents a critical knowledge gap on the path to developing more effective immunotherapies.

In contrast to TILs, cancer-associated fibroblasts (CAFs) and high stromal content in general are negative prognostic factors in HGSC and other cancers (19–27). Indeed, numerous studies have identified a “C1/Mesenchymal” (C1/MES) subtype of HGSC that is enriched in CAFs and associated with poor prognosis (3, 28, 29). However, the methods used to define stroma vary widely between studies and generally overlook the fact that TILs can dominate the stromal compartment of immunologically active tumors (11). Thus, the relationship between tumor stroma and antitumor immunity is complex, and the relative influence of these factors on long-term survival is unknown.

Gene expression profiling studies have generally converged on 4 biologically relevant molecular subtypes of HGSC (3, 15, 29–32). As mentioned, C1/MES tumors have the highest stromal and CAF content. C2/Immunoreactive (C2/IMM) tumors are enriched for T cells, B cells, and other immune cells. C4/Differentiated (C4/DIF) tumors express higher levels of MUC16 and other epithelial gene products and have moderate levels of immune cells. C5/Proliferative (C5/PRO) tumors express gene products associated with stem cells, cell cycle, and epithelial-to-mesenchymal transition and have negligible immune-cell infiltration. The C1/MES subtype has been associated with poor survival in most studies, whereas the C2/IMM subtype and, in many cases, the C4/DIF subtype have been associated with prolonged survival (3, 15, 29–33).

Here, we report a systematic analysis of TIL subsets, epithelial/stromal content, and molecular subtype in the MOCOG cohort, which revealed what we believe to be a novel intersection between these factors and long-term survival in HGSC.

## Results

### Diverse immune-cell subsets are associated with long-term survival.

**Figure 1:**
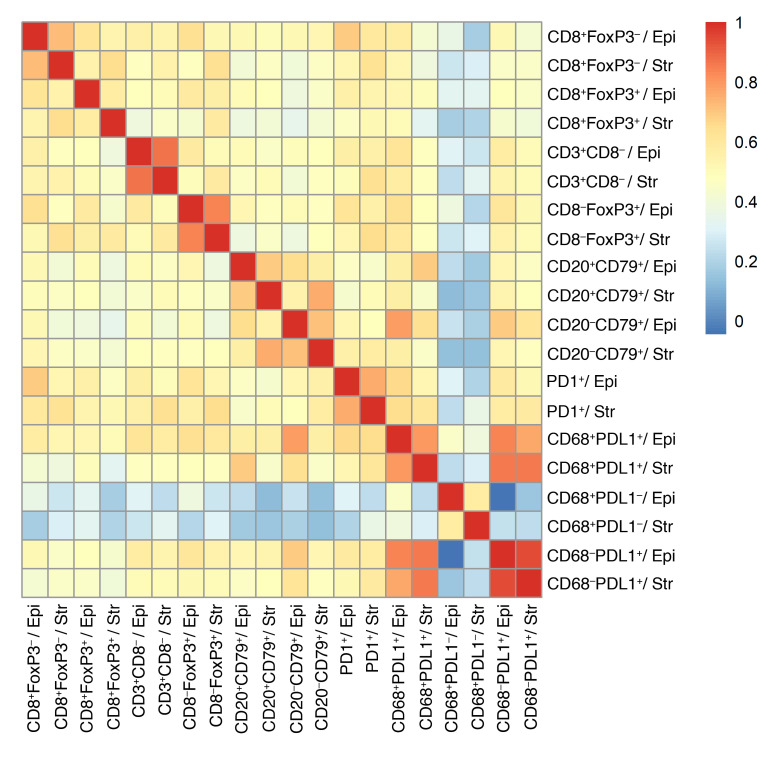
Heatmap showing pairwise Spearman correlations between immune cell subsets. The color scale indicates the strength of the correlation between densities with red indicating high positive correlation. Epi, intraepithelial location; Str, intrastromal location of immune cells.

Table 1 and Supplemental Table 1 (Supplemental Material available online with this article; https://doi.org/10.1172/JCI179501DS1) summarize key features of the 1,223 evaluated participants (374 LTS, 433 MTS, 416 STS). Multi-color IHC or immunofluorescence (IF) was used to determine the densities of 10 immune-cell subsets in the epithelial and stromal regions of the tumors; an 11th immune-cell subset was generated by summing CD8^+^FoxP3^–^ and CD8^+^FoxP3^+^ T cell counts to create a single “CD8^+^ T cell” measure (Table 2). Immune cells were generally more abundant in stromal than epithelial tumor regions. Most immune-cell subsets were positively correlated (Figure 1), the only exception being CD68^+^PD-L1^–^ TAMs, which showed no association with other immune-cell types.

**Figure 2:**
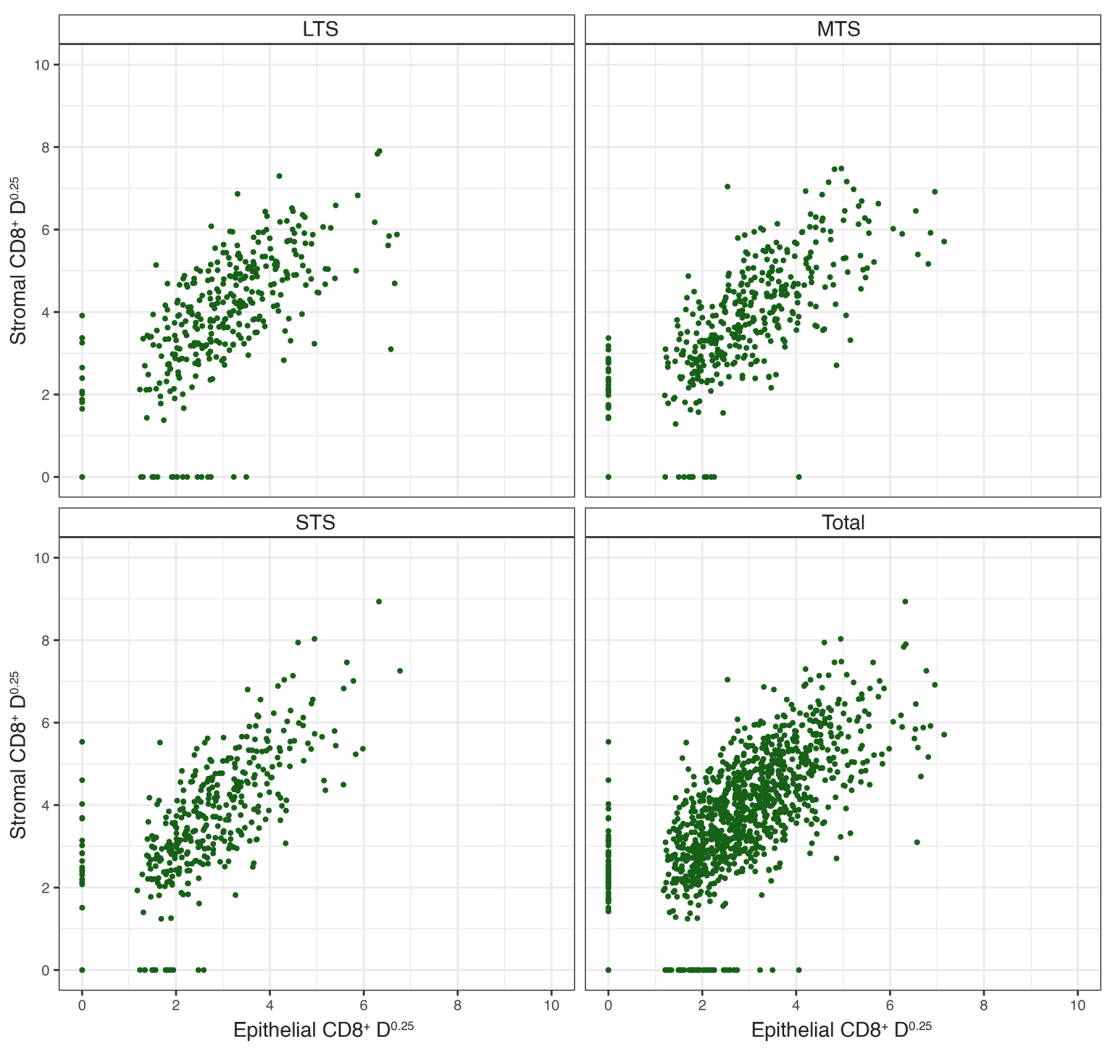
Intrastromal versus intraepithelial CD8^+^ TIL densities (cells/mm^2^) in tumors from all participants and from the STS, MTS, and LTS subgroups. The relationship of the intrastromal CD8^+^ density values to the intraepithelial CD8^+^ density values showed no differences between STS and LTS (*P* = 0.60).

In HGSC and other cancers, an “immune excluded” TIL pattern has been described in which T cells are predominantly restricted to tumor stroma rather than epithelium (34). To assess this, we plotted intraepithelial versus intrastromal CD8^+^ T cell densities (Figure 2). Although a small number of tumors were devoid of intraepithelial CD8^+^ cells or intrastromal CD8^+^ cells (which was likely attributable to the small size of the tissue microarray [TMA] cores), we found no evidence of a distinct subgroup of tumors with substantial intrastromal values and negligible intraepithelial values overall or individually for the LTS, MTS, and STS groups (Figure 2). Thus, immune exclusion was not evident in our cohort, and we did not consider this or other spatial patterns in subsequent analyses.

Table 3 shows the ORs (using D^0.25^; see Methods and Statistics) comparing LTS to STS, LTS to MTS, and MTS to STS for all immune-cell subsets in epithelium and in stroma. In the LTS to STS comparison, with the exception of CD68^+^PD-L1^–^ TAMs, all immune-cell subsets were substantially more abundant in LTS than STS in the epithelium and/or stroma. Within the epithelial compartment, we found highly statistically significant associations with survival for intraepithelial CD8^+^ T cells (including both FoxP3^–^ and FoxP3^+^ subsets), PD-1^+^ immune cells, CD68^–^PD-L1^+^ cells (presumptive PD-L1^+^ tumor cells), and B cells. Within the stromal compartment, the most significant associations (*P* ≤ 0.002) were seen for CD8^+^FoxP3^+^ T cells, PD-1^+^ cells, CD20^–^CD79^+^ plasma cells, and CD68^–^PD-L1^+^ cells. Intra-epithelial CD8^+^FoxP3^+^ T cells and PD-1^+^ cells had the strongest associations based on OR magnitude. Most immune marker associations for MTS versus STS were substantially weaker than those for LTS versus STS (Table 3). Results restricted to participants whose samples were known to be adnexal (data not shown) or who were known to have received primary cytoreductive surgery (PCS) (Supplemental Table 2) were not materially different from our main results (data not shown).

### Prognostic effects of TILs are strongest in tumors with high epithelial content.

**Figure 3:**
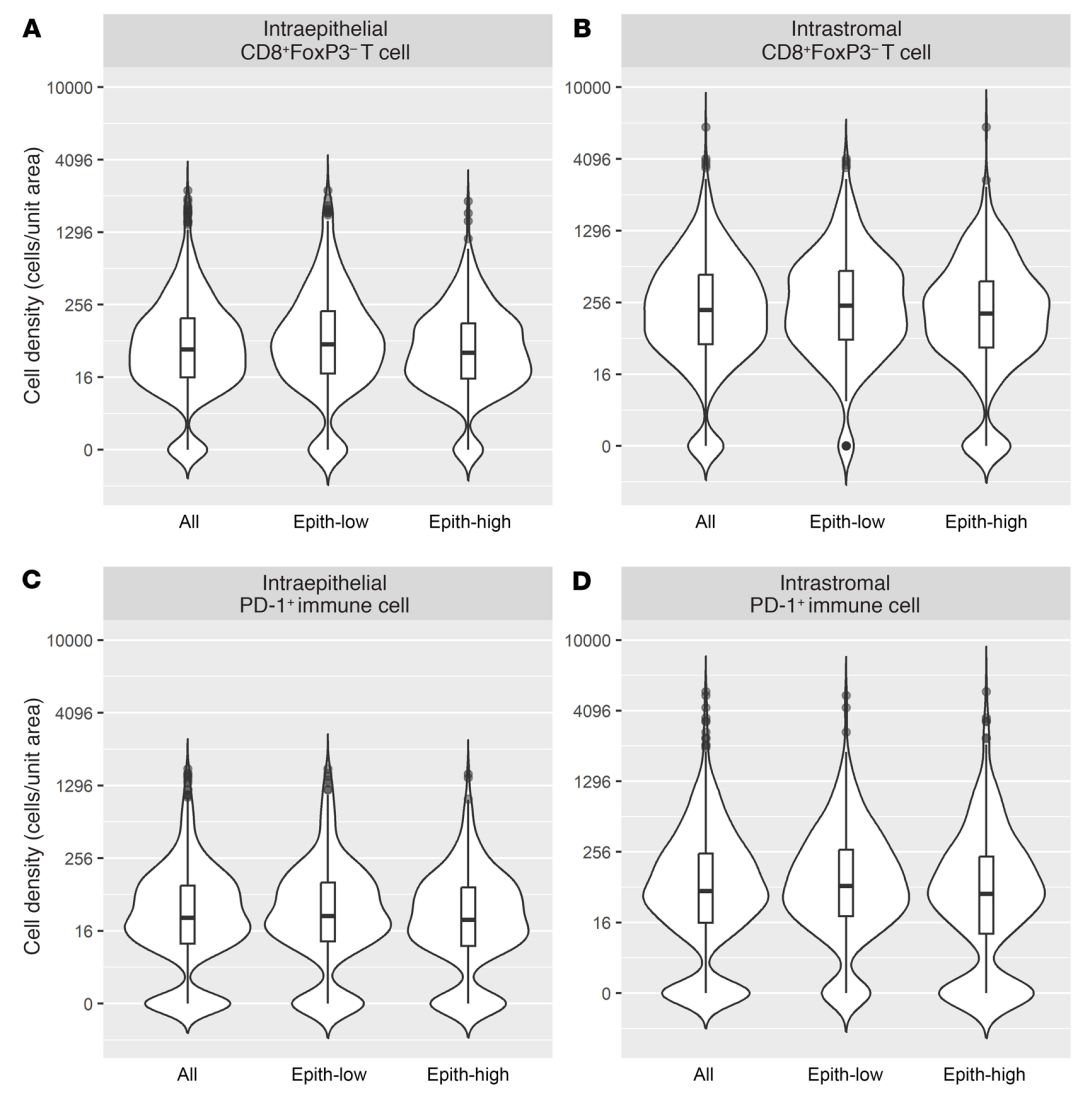
Violin plots comparing the densities of immune cell subsets in epithelium-high versus –low tumors. (**A**) Density of intraepithelial CD8^+^FoxP3^–^ T cells in all tumors, epithelium-low tumors, and epithelium-high tumors. (**B**) Intrastromal CD8^+^FoxP3^–^ T cells. (**C**) Intraepithelial PD-1^+^ immune cells. (**D**) Intrastromal PD-1^+^ immune cells. Embedded box plots indicate median (horizontal line), quartile (box edges), and outliers (points).

To investigate whether epithelial-to-stromal content influenced the magnitude or prognostic significance of immune-cell infiltrates, patients were stratified into epithelium-high versus -low groups (see Supplemental Methods and Statistics). Epithelial content showed no significant association with LTS, MTS, or STS groups. The densities of almost all immune-cell subsets (both intraepithelial and intrastromal) were higher in the epithelium-low group, with the exception of intraepithelial CD68^+^PD-L1^+^ TAMs and intra-epithelial and intrastromal CD68^+^PD-L1^–^ TAMs (Table 2). For example, Figure 3 compares the densities of CD8^+^FoxP3^–^ T cells and PD-1^+^ cells in epithelium-high versus epithelium-low tumors.

**Figure 4:**
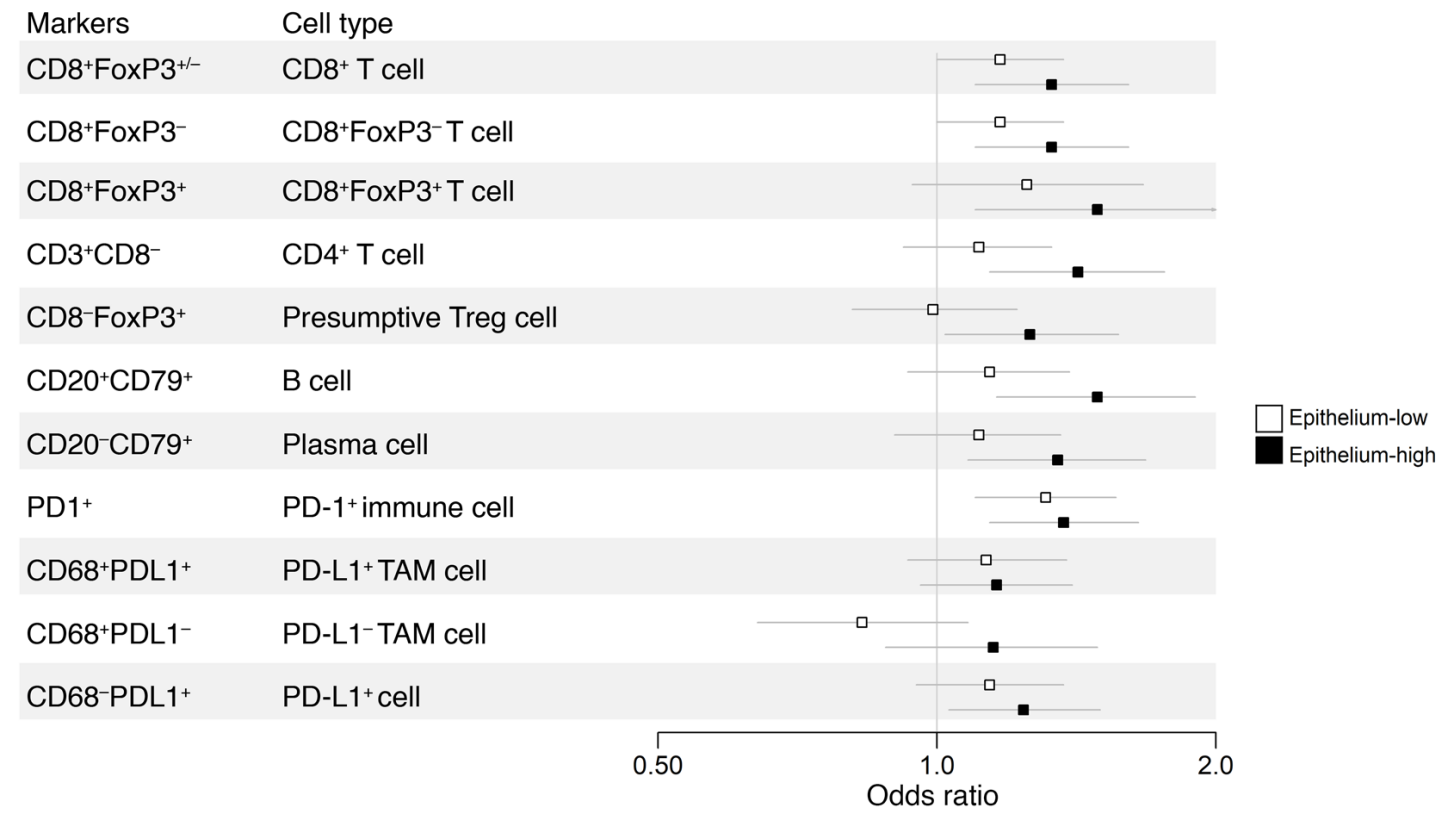
Forest plot of the odds ratios and 95% CIs of LTS compared with STS for intraepithelial immune cell subsets stratified by epithelium-high versus epithelium-low tumors.

We next evaluated the impact of epithelial content on the prognostic effects of immune markers. Table 4, Figure 4, and Supplemental Figure 1 show a comparison of the LTS and STS groups. Immune marker associations were markedly stronger in the epithelium-high group compared with the epithelium-low group. In the epithelium-high group, 9 of 11 intraepithelial (Table 4, Figure 4) and 10 of 11 intrastromal (Supplemental Figure 1) immune-cell subsets showed a statistically significant association with survival groups (LTS versus STS); this included all T cell, B cell, and plasma cell subsets. By contrast, in the epithelium-low group, only intraepithelial CD8^+^ T cells and PD-1^+^ cells were statistically significantly associated with survival group. Thus, the association of TILs with LTS was largely restricted to tumors with high epithelial content, even though TIL densities were generally higher in epithelium-low tumors (Figure 3). Similar results were obtained when quartile (see Methods section, Statistics) OR results were used to compare LTS to STS by epithelium group (Supplemental Table 3). In the MTS versus STS comparison, the influence of epithelial content was evident but less striking (Supplemental Table 4).

Further analyses of immune-cell subsets were performed in 2 population-based HGSC cohorts: Canadian Ovarian Experimental Unified Resource (COEUR) (*n* = 981) (35) and Ovarian Outcomes Unit (OOU) (*n* = 192) (36); the latter had previously been stained to detect T cell phenotypes (including CD39^+^ and CD103^+^ T cells) that were not evaluated in the MOCOG or COEUR panels. Similar to the MOCOG findings, these analyses showed that the prognostic effects of most TIL subsets were restricted to epithelium-high tumors, despite immune-cell densities being generally higher in epithelium-low tumors (Supplemental Material and Supplemental Tables 5–8).

### Intrastromal B cells complement the prognostic effect of intraepithelial CD8^+^ T cells.

We investigated which combination of immune cells best predicted outcome in the epithelium-high group. Because of the well-accepted importance of intraepithelial CD8^+^ T cells, we included this marker in the analysis a priori. After accounting for intraepithelial CD8^+^ T cells, intrastromal B cells were the only other immune-cell subset that distinguished LTS and STS (Supplemental Table 9). PD-1^+^ immune cells and CD8^+^ T cell densities were highly correlated, and interchanging PD-1^+^ and CD8^+^ left the conclusions unchanged. In a joint effects model (Table 5), an OR of 4.87 was observed for the highest quartile for intraepithelial CD8^+^ T cells and nonzero for intrastromal B cells (*P* < 0.001, with no statistical interaction between these 2 markers [*P* > 0.05]).

### The prognostic effects of immune cells depend on molecular subtype.

Molecular subtyping data was available for 217 LTS, 251 MTS, and 226 STS MOCOG cases (33). Across all 3 groups combined, the molecular subtypes were distributed as: C1/MES (24%), C2/IMM (27%), C4/DIF (33%), and C5/PRO (16%). There was no statistically significant association between molecular subtype and survival group. Consistent with prior reports (33, 37), epithelial content was highest in the C4/DIF subtype (median, 75%), lowest in the C1/MES subtype (median, 57%), and intermediate in the C2/IMM (median, 67%) and C5/PRO subtypes (median, 69%), (*P* < 0.001).

**Figure 5:**
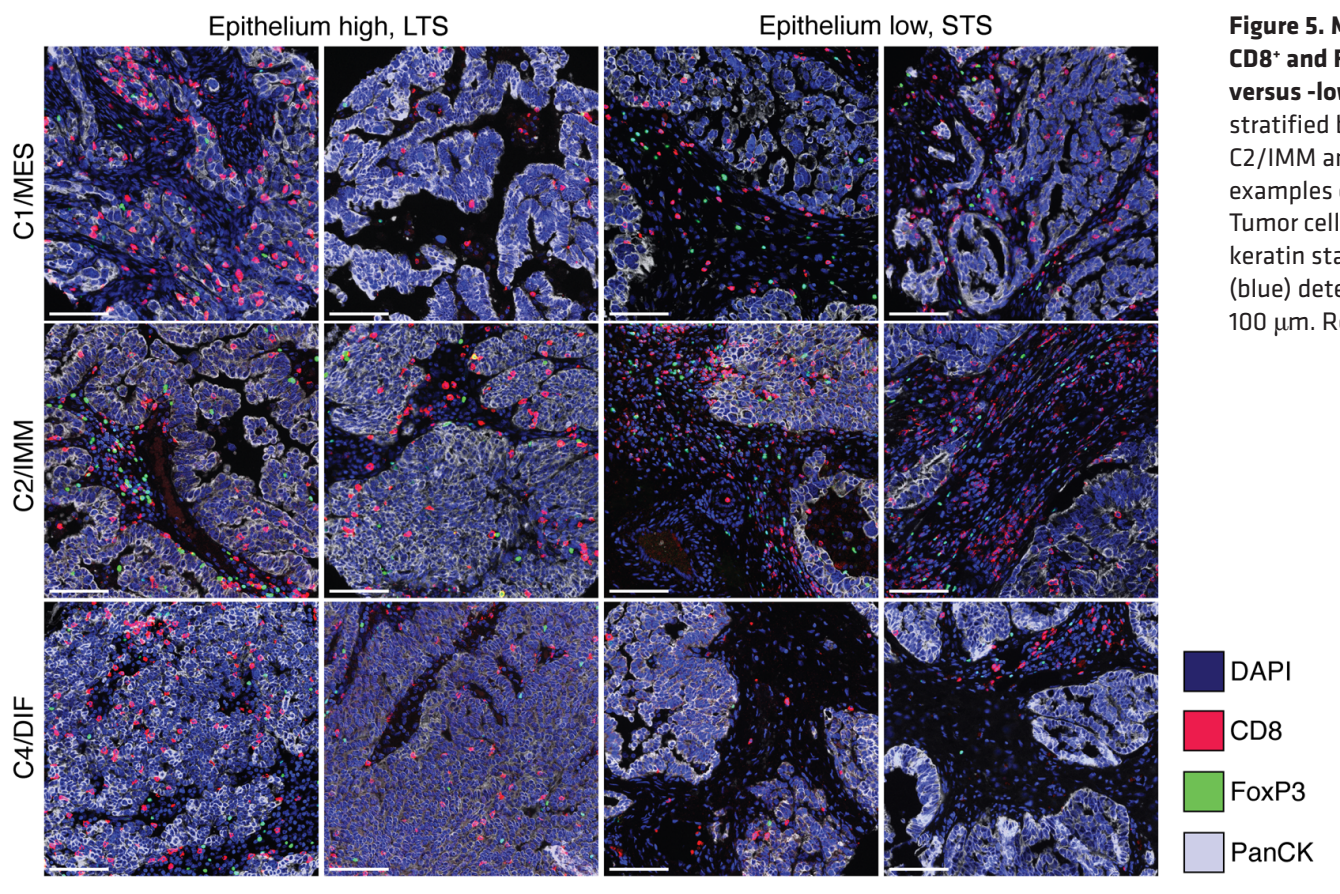
Multiplex IF images showing CD8^+^ and FoxP3^+^ TILs in epithelium-high versus -low tumors. Images further stratified by molecular subtype (C1/MES, C2/IMM and C4/DIF); 2 representative examples of each subgroup are shown. Tumor cells are highlighted by pan-cytokeratin staining (light gray). DAPI staining (blue) detects all cell nuclei. Scale bars: 100 μm. Red, CD8^+^; green, FoxP3^+^.

As expected, the C2/IMM subtype had the highest median densities of all intraepithelial and intrastromal immune-cell subsets, followed in order by the C1/MES, C4/DIF, and C5/PRO subtypes (Table 6 and Figure 5). For most immune-cell subsets, the ratio of intrastromal to intraepithelial cell densities did not vary substantially between molecular subtypes.

**Figure 6:**
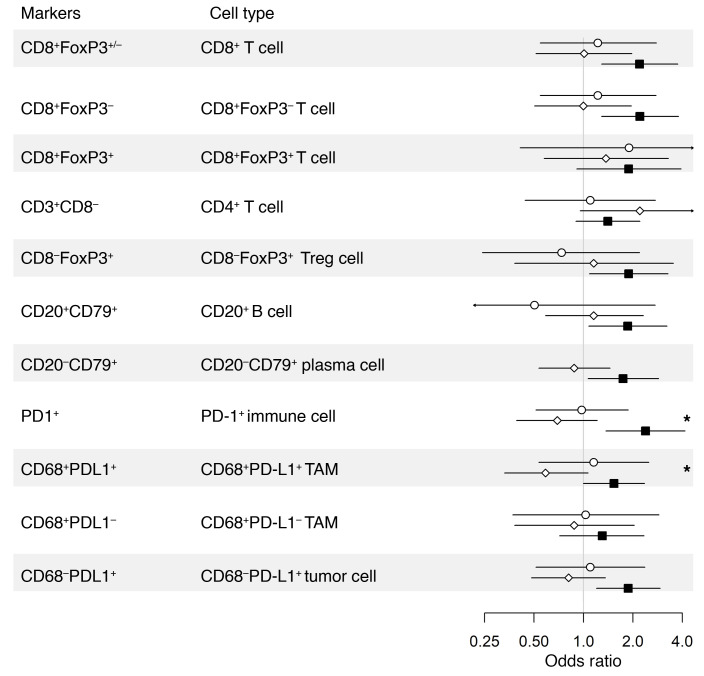
Forest plot of the odds ratios and 95% CIs of LTS compared with STS of intraepithelial immune-cell subsets for the C1/MES, C2/IMM, and C4/DIF molecular subtypes in epithelium-high cases. Plasma cell results for the C1/MES subtype could not be calculated. The C5/PRO subtype is not presented as several could not be calculated. *P < 0.05 for heterogeneity across subtypes.

Unexpectedly, the association between immune cells and LTS was near exclusive (with only 1 exception) to the C4/DIF subtype. Statistically significant positive associations were seen for 5 of 11 intraepithelial and 4 of 11 intrastromal cell subsets (Table 7, Figure 6, and Supplemental Figure 2), despite the fact that C4/DIF tumors ranked third among the molecular subtypes with respect to the densities of 19 of 22 immune-cell subsets (Table 6). In contrast, the C2/IMM molecular subtype had the highest median levels of all intraepithelial and intrastromal immune-cell subsets (Table 6), yet only intraepithelial CD8^+^FoxP3^+^ T cells showed a statistically significant association with LTS within this molecular subtype (Table 7).

To investigate whether these C4/DIF results reflected the influence of tumor epithelium, we restricted the comparison between LTS and STS to epithelium-high tumors (Table 8). As expected (33, 37), a higher proportion of C4/DIF tumors were epithelium-high (*n* = 104 of 155, 67%) compared with C1/MES (*n* = 31 of 109, 28%), C2/IMM (*n* = 61 of 108, 56%), and C5/PRO tumors (*n* = 33 of 71, 46%) (Tables 7 and 8). Strikingly, within epithelium-high tumors, the prognostic significance of immune cells was exclusive to the C4/DIF subtype, with 8 of 11 intraepithelial and 6 of 11 intrastromal subsets showing a statistically significant association with LTS. There were no statistically significant associations within the other molecular subtypes (Table 8).

## Discussion

The immunological and microenvironmental features associated with LTS had remained largely undefined in HGSC owing to the rarity of such patients and the paucity of biospecimens with sufficient follow-up data. To address this critical gap, we assembled a large international cohort comprised of similar numbers of STS, MTS, and LTS patients. The TME of LTS cases was distinguished by coinfiltration of T cells, B cells, and plasma cells, along with upregulation of the PD-1/PD-L1 pathway. Remarkably, these prognostic associations were almost entirely restricted to tumors with high-epithelial content and the C4/DIF molecular subtype, factors that have not previously been implicated as influencing the prognostic effects of TILs and TAMs. These findings were not attributable to higher TIL or TAM densities in epithelium-high or C4/DIF tumors, indicating that they instead reflect other biological features associated with these tumor subgroups. Our findings suggest that immunotherapies for HGSC should be designed to engage not only T cells but also the B cell and myeloid cell lineages. They further suggest that the immunobiology of epithelium-high, C4/DIF tumors warrants further study to understand their apparent enhanced susceptibility to immune-based control mechanisms.

In addition to TILs, intraepithelial CD68^+^PD-L1^+^TAMs showed an association with LTS, consistent with prior reports (16, 38). While PD-L1 has a well-established immunosuppressive role, it is also an indicator of active TIL responses, which may explain the favorable prognostic association (16). Apart from PD-L1, TAMs can suppress antitumor immunity through a variety of other mechanisms, and future studies are warranted to assess the influence of these additional factors on long-term outcomes (39).

In principle, our finding that the prognostic effect of immune cells is attenuated in epithelium-low tumors could reflect the immunosuppressive properties of CAF-rich tumor stroma (19–27, 40). In particular, the C1/MES subtype of HGSC is characterized by desmoplastic stroma and, reportedly, a preferential localization of T cells in tumor stroma (28, 29). This “immune-excluded” pattern has also been reported in other cancers, although a consensus definition has not been reached (41). Despite these prior reports, we saw no evidence of a distinct immune-excluded subgroup of tumors in the STS, MTS, or LTS groups. Moreover, we found that epithelium-low tumors had higher average densities of almost all immune-cell subsets (except CD68^+^PD-L1^–^ TAMs) in both the epithelial and stromal compartments. Therefore, while CAFs and/or other stromal elements could explain the blunted prognostic effect of TIL in epithelium-low tumors, immune exclusion does not appear to be the underlying mechanism. Notably, McGregor and colleagues found that epithelial content had a minimal influence on TIL densities and activation profiles in HGSC; instead, epithelium-high tumors showed evidence of increased activation of cross-presenting dendritic cells, which could activate tumor-specific CD8^+^ T cells (42). Thus, further studies are warranted to assess the functional status of TILs in epithelium-high versus -low tumors.

Our finding that the prognostic effect of TILs is restricted to epithelium-high tumors has implications for TIL scoring in the HGSC setting. Several studies have scored cases based on the absolute number of intraepithelial T cells per field (4, 7); presumably epithelium-high tumors would score higher with this approach, which could inadvertently amplify the prognostic effect of TILs. Furthermore, exclusion of tumor cores with low epithelial content would enrich for epithelium-high tumors, again amplifying the prognostic significance of TILs. Thus, epithelial content is an important variable in immune-related prognostic studies.

To our knowledge, this is the first study to assess the prognostic significance of TILs across the 4 molecular subtypes of HGSC, which led to the unexpected finding that the association between TILs and LTS is almost entirely restricted to the C4/DIF subtype. A relatively large yet understudied group, C4/DIF tumors were initially reported to have only moderate TIL levels yet favorable prognostic significance on par with the C2/IMM subtype (29). The TCGA study named this the “Differentiated” subtype based on higher expression of epithelial markers (e.g., MUC16 and MUC1), which was suggested to reflect a more mature stage of development (3). Wang and colleagues identified within the C4/DIF subtype a fifth molecular subtype they called “antimesenchymal” owing to the downregulation of genes associated with the C1/MES subtype (43). This novel subtype, which could represent the epithelium-high C4/DIF subset reported here, was associated with the longest survival among the 5 subtypes (43). Talhouk and colleagues also found that C4/DIF tumors were associated with high patient survival rates, equivalent to C2/IMM tumors (33). Moreover, C4/DIF tumors were more likely to have an adnexal location and exhibited high tumor purity, equivalent to C5/PRO tumors (33). Intriguingly, C4/DIF tumors were also associated with a younger age at diagnosis (33), which was also reported by Tothill and colleagues (29).

Waldron and colleagues too found that C4/DIF tumors were associated with younger patient age and higher tumor purity (37). They further showed that C4/DIF tumors had lower ploidy, lower copy number variation, and lower subclonality compared with the other molecular subtypes, consistent with a lower number of genome doublings (37). In contrast, C5/PRO tumors showed a higher number of gene amplifications, higher ploidy, and increased frequency of genome duplication. Single-cell RNA-seq (scRNA-seq) revealed that the majority of tumor cells (as opposed to other cell types in the admixture) exhibited a C4/DIF transcriptional signature, with the remaining tumor cells being assigned a C5/PRO signature (37), a finding consistent with other scRNA-seq studies (44, 45). They proposed that C4/DIF and C5/PRO tumors represent different ends of an evolutionary time scale — more recently arising tumors versus older tumors, respectively — and that the C1/MES and C2/IMM subtypes are derivatives whose signatures merely reflect the presence of mesenchymal and immune cells (37). If this hypothesis is correct, the favorable immunological and prognostic associations seen with C4/DIF tumors could be attributable to evolutionarily “younger” tumors, earlier diagnosis, age at diagnosis, and/or lower intratumoral heterogeneity, features that have previously been linked to improved prognosis (46–48) and could plausibly facilitate more effective antitumor immunity.

Finally, the prognostic benefits of C4/DIF tumors could also have an immunological explanation. Applying scRNA-seq to primary HGSC samples, Olbrecht and colleagues found that tumor cells mapping to the C4/DIF subtype were enriched for transcripts reflecting IFN signaling, suggesting exposure to an active immune response (45). Owing to their more differentiated state, C4/DIF tumors could also have higher densities of antigenic epitopes for recognition by CD8^+^ T cells; for example, peptides derived from MUC16 are predominant components of the MHC class I and class II peptide landscape in HGSC (49). Thus, C4/DIF tumor cells and tumors exhibit distinct clinical, histological, transcriptional, genomic, and immunological features that warrant further study as potential determinants of patient survival.

C2/IMM tumors had the highest densities of all immune-cell subsets; however, with the exception of CD8^+^FoxP3^+^ T cells, immune cells showed no statistically significant association with LTS within this molecular subtype. This finding was unexpected given the well-established prognostic benefit of TILs in HGSC (50), including the report of a positive dose-response association between intraepithelial CD8^+^ T cells and survival (4). This could reflect a dynamic range issue wherein most C2/IMM cases have immune cell densities that exceed the threshold required to promote LTS. If so, however, it is unclear why the C2/IMM subtype did not show a stronger association with LTS relative to the other molecular subtypes. Another possible explanation is that prior studies have not been as highly enriched for LTS cases as the present study. For example, the TCGA (30) and Predictor of high-grade serous Ovarian carcinoma molecular subtype (PrOTYPE) (33) studies had only 1% and 8.4% LTS cases, respectively, and may have been underpowered to detect the combined effects of immune cell infiltrates and C4/DIF subtype on LTS shown here.

The substantial prognostic effect of B cells and plasma cells shown here aligns with prior reports in HGSC (8, 11–14, 51) and the emerging appreciation for the role of the B cell compartment in antitumor immunity in other cancers (52, 53). Furthermore, in a recent genomic/transcriptomic study of HGSC, we found that plasma cell gene signatures were independent predictors of LTS along with *BRCA2*-type homologous recombination deficiency, *PCNA* expression, and residual disease (10). It was recently proposed that the prognostic benefit of plasma cells may be impaired when they colocalize with CAFs, (54) which fits with our finding that the prognostic benefit of plasma cells and B cells is strongest in epithelium-high (presumably CAF low) tumors. With respect to possible effector mechanisms, B cells and plasma cells can potentially enhance T cell responses by helping to organize lymphoid aggregates, including tertiary lymphoid structures, and by serving as antigen-presenting cells (52, 53). Indeed, a hallmark of “exhausted/dysfunctional” tumor-infiltrating CD8^+^ T cells in HGSC and other cancers is expression of the B cell–recruiting chemokine CXCL13 (36, 55–57), suggesting that T cells are programmed to solicit B cell help in the face of chronic antigen stimulation. Accordingly, we found that tumors containing dense intraepithelial CD8^+^ T cells (highest quartile) combined with intrastromal CD20^+^ B cells were associated with an almost 5-fold higher likelihood of being LTS versus STS. In addition to T cell–based mechanisms, the antibodies produced by plasma cells in HGSC have been shown to bind tumor antigens (8, 12) enabling them to potentially block the function of their target protein directly and/or engage innate effector mechanisms such as complement-mediated cytotoxicity, antibody-dependent cellular phagocytosis by TAMs, and antibody-dependent cellular cytotoxicity by natural killer and TAM cells (52, 53). These latter mechanisms could explain the coassociations between CD68^+^ PD-L1^+^ TAM cells and various TIL subsets and their marked enrichment in LTS cases.

Our study has several potential limitations. While the use of TMAs from 19 different studies, 4 continents and a 26-year time frame should increase the generalizability of our findings, it also presented technical challenges related to immunohistological staining and scoring. To mitigate variability in specimen age and quality, we deployed 4 small, robust panels of markers; this restricted our ability to definitively identify some cell types (e.g., Tregs, plasma cells) and to assess nearest-neighbor relationships between diverse immune-cell subsets in the same tissue section. A related limitation was the use of TMAs instead of whole sections, which reduced our ability to detect prognostically relevant immune aggregates, including tertiary lymphoid structures (11). TMAs also may not capture the full heterogeneity of TIL and TAM patterns (58), but this issue would be partly mitigated by our large sample sizes. The use of TMAs is also relevant to our classification of cases into epithelium-high versus -low groups, as the tumor cores on TMAs represent only a small fraction of a patient’s overall tumor burden and are typically punched from areas with the highest epithelial content. Indeed, the cores used for TMA construction were selected by each study’s pathologist, resulting in nonstandardized epithelial versus stromal content between studies; however, such variation was mitigated by the large sample size of the MOCOG cohort and the corroborating results obtained from the COEUR and OOU cohorts. A further limitation is that we defined stromal regions by the absence of epithelial features rather than directly staining for markers of CAFs, endothelium, or other stromal cell types that can influence prognosis (19–27). Thus, an important future direction will be to assess immune cells in larger tumor regions using more highly multiplexed methods (which were not available when this study was initiated) that include detection of key stromal cell types. Use of such methods will also enable analysis of the spatial relationships between cell types, which can have a substantial influence on antitumor immunity and patient survival (59–61). It will also be important to validate new findings in independent cohorts, in particular what we believe to be the novel influence of epithelial content and C4/DIF molecular subtype on the prognostic effect of TIL. Finally, our use of a high threshold for LTS (≥ 10 years) necessitated inclusion of patients predominately from the 2000’s; therefore, contemporary treatment regimens (e.g., angiogenesis and PARP inhibitors) were not well represented in the cohort. In future studies, it will be important to determine how these newer therapeutic agents modify the relationship between tumor biology and patient survival.

Our findings have implications for the treatment of HGSC. First, they provide further justification for the development of combination immunotherapies that coordinately enhance the orthogonal effector mechanisms used by T cells, B-lineage cells, and myeloid cells (52, 53). Second, they challenge the notion that immune exclusion is a major barrier in C1/MES tumors given these tumors harbored relatively abundant TILs (similar or higher than C4/DIF tumors) in both the epithelial and stromal compartments. Finally, our work suggests that epithelium-high and/or C4/DIF tumors may represent especially attractive targets for immunotherapy and could help elucidate the critical immune barriers present in other subtypes. In this regard, C4/DIF tumors represent the largest molecular subtype (33.2% of cases in the PrOTYPE study) (33), yet they have received the least investigation from an immunological perspective. By resembling normal epithelium more closely, C4/DIF tumors may be more conducive to immune-mediated control mechanisms. For example, there is growing appreciation of the importance of biomechanical forces in immune surveillance and tumor cell killing (62). This could provide rationale for combining immunotherapy with pharmaceutical agents that promote tumor cell differentiation and/or a more normal epithelial architecture (63). Thus, C4/DIF tumors may hold important clues for developing the next generation of immunotherapies for HGSC and related malignancies.

## Methods

### Sex as a biological variable.

This study was focused exclusively on HGSC, a disease which affects only biological females.

### Study population and tumor samples.

The MOCOG cohort was assembled from studies in Australia, Europe, North America, and Brazil. Each participating study received local ethics review board approval. Specimens were obtained with written informed consent (or a formal waiver of consent) with approval by the relevant ethics review board. Of *n* = 1,298 total tumors, 1,223 were successfully stained and scored (Supplemental Table 1). Patients were diagnosed between 1985 and 2011 with FIGO Stage III/IV ovarian, fallopian tube, or primary peritoneal HGSC. Survival groups were defined as LTS (10+ years), MTS (5–7.99 years) and STS (2–4.99 years) from the date of diagnosis. STS and MTS were frequency matched to LTS by study, year of diagnosis, and patient age at diagnosis. Supplemental Figure 3 illustrates the study design. Studies constructed their own TMAs from formalin-fixed paraffin-embedded (FFPE) blocks of tumor tissue. TMA cores were 0.6–1.0 mm from areas selected by each study’s pathologist. 34.1% of cases had 1 core, 58.4% 2 cores, 7.1% 3 cores, and 0.4% 4 cores. See Supplemental Materials for additional details.

### Immune marker staining and scoring.

All staining and scoring were performed at BC Cancer, Victoria. MOCOG TMAs were stained by multicolor IHC or IF with 4 panels of antibodies: (64) panel A detected CD3 and CD8; panel B detected CD20 and CD79; panel C detected CD8, FoxP3, and CD25; and panel D detected PD-1, PD-L1, and CD68. All panels detected pancytokeratin to identify tumor epithelium. CD4^+^ T cells were defined as CD3^+^CD8^–^ cells (36). See Supplemental Methods for additional details and information pertaining to antibodies.

### Statistics.

Immune-marker density (D; cells/mm^2^) for a particular marker was calculated separately for epithelial and stromal compartments. For cases with multiple cores, the epithelial area was taken as the sum of all their individual TMA epithelial areas and similarly for the stromal area. For analyses including all participants, we transformed the densities (D) by raising them to the power 0.25 (D^0.25^); this transformation gave close to the maximum log-likelihood of the fitted models across the range of immune subsets and substantially reduced the skewness of the distribution of the values (65). To provide categorical comparisons and to better appreciate the magnitude of the associations, we also categorized marker D values into quartiles separately for the 5 largest studies (AUS, DOV, MAY, SEA, and VAN), based on the distribution of the D values of the STS, separately for epithelial and stromal markers. If the proportion of zero D values was greater than 50% overall, we compressed the quartile values into 2 categories (zero, nonzero). Quartile analyses was not appropriate for the remaining studies due to smaller sample sizes. Statistical significance was defined as *P* ≤ 0.05. No adjustments were made for multiple testing. Analyses were conducted using R Studio (version 1.3.1073) and Stata (version 16). See Supplemental Materials for additional details.

### Data availability.

Individual patient data and related tumor information underlying this article cannot be shared publicly due to data privacy protection laws. Requests for further analyses to be done on a collaborative basis will be addressed on reasonable request to the corresponding author. No custom code or software was used.

## Author contributions

BHN, MCP, and CLP conceptualized and designed the study, provided resources, supervised the study, analyzed data, were responsible for administration of the project, and wrote the original manuscript and revised the final draft of the manuscript. PH and MTP analyzed data, wrote the original manuscript, and provided feedback on the final draft of the manuscript. DDLB, AD, SJR, JDB, and PDPP conceptualized the study, provided resources, and gave critical feedback on the original and final drafts of the manuscript. DWG, AK, HS, MA, JR, CMD, AC, BG, and AMP gave critical study design input and feedback on the original and final drafts of the manuscript. KM, BH, ST, DS, S Kalaria, KS, CML, EM, and AA generated and analyzed data. NSM, AB, SF, CJK, JH, DA, KA, and NT managed data and acquisition of resources. NS, KLT, BK, JL, MEC, GEH, AGM, KLCH, and AS provided resources and input on the final draft of the manuscript. ELG, JAD, HRH, KH, DH, AT, JA, MJL, JB, AHB, PRH, RS, JLH, MTG, BYH, LRW, SB, RTF, PAF, CB, FJCR, PG, MK, EE, FM, LC, NL, UM, MJG, CRA, KF, LEK, and S Kommoss provided resources.

## Supplementary Material

Supplemental data

ICMJE disclosure forms

## Figures and Tables

**Table 5:**
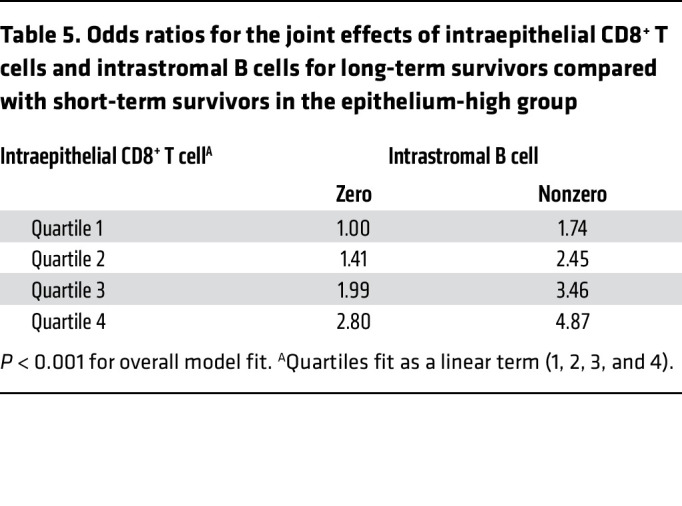
Odds ratios for the joint effects of intraepithelial CD8^+^ T cells and intrastromal B cells for long-term survivors compared with short-term survivors in the epithelium-high group

**Table 4:**
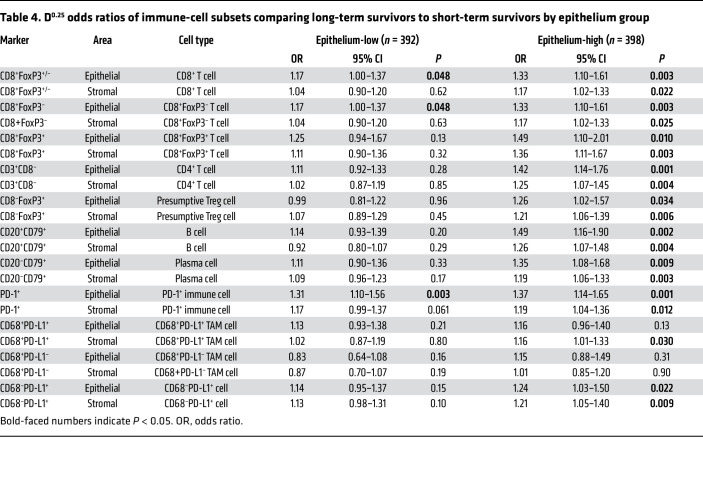
D^0.25^ odds ratios of immune-cell subsets comparing long-term survivors to short-term survivors by epithelium group

**Table 3:**
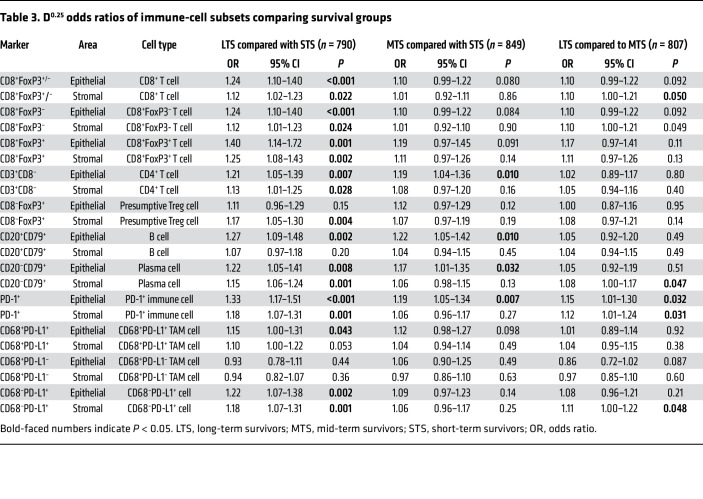
D^0.25^ odds ratios of immune-cell subsets comparing survival groups

**Table 2:**
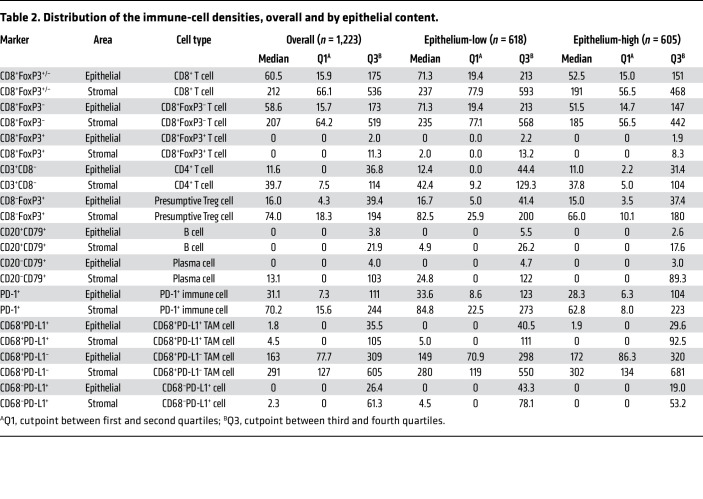
Distribution of the immune-cell densities, overall and by epithelial content.

**Table 1:**
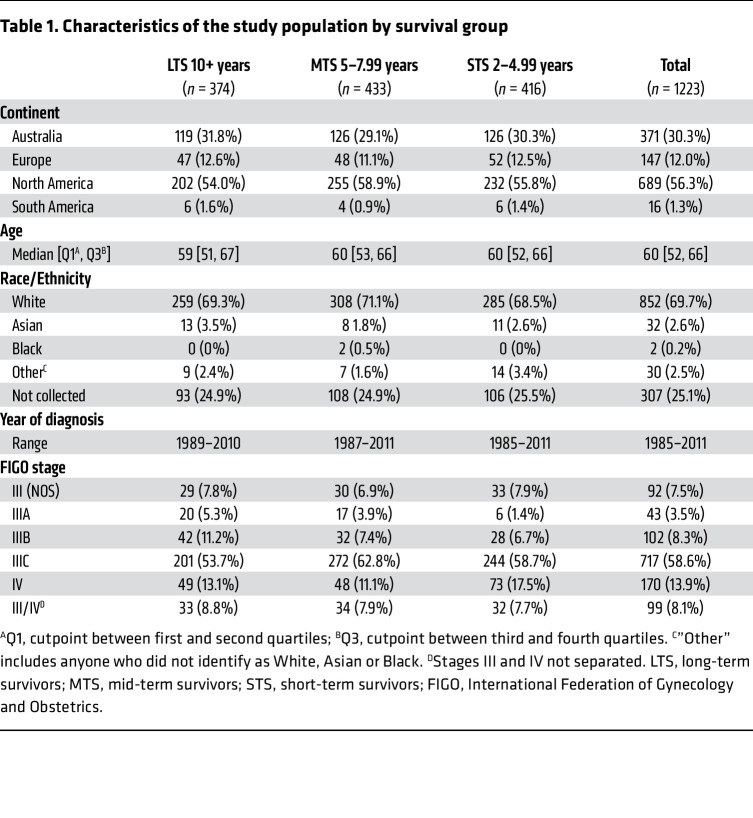
Characteristics of the study population by survival group

**Table 6:**
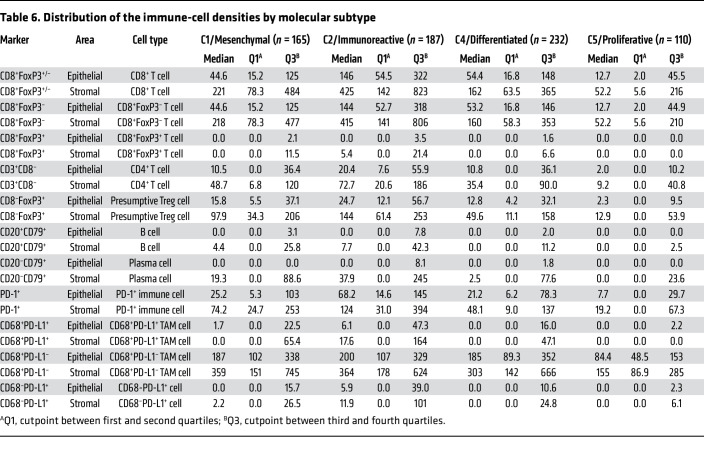
Distribution of the immune-cell densities by molecular subtype

**Table 7:**
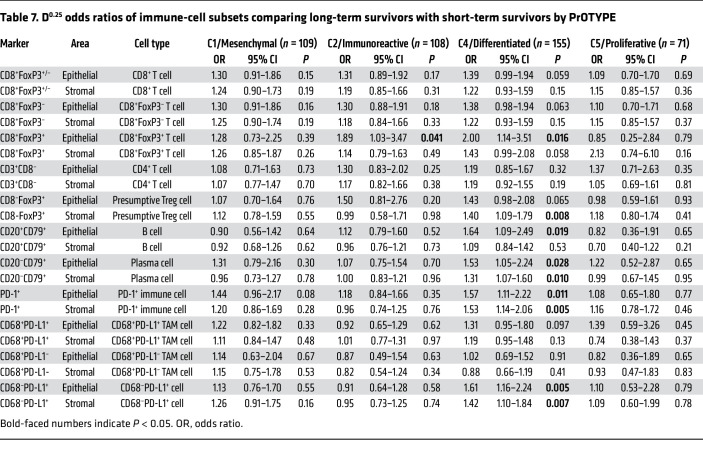
D^0.25^ odds ratios of immune-cell subsets comparing long-term survivors with short-term survivors by PrOTYPE

**Table 8:**
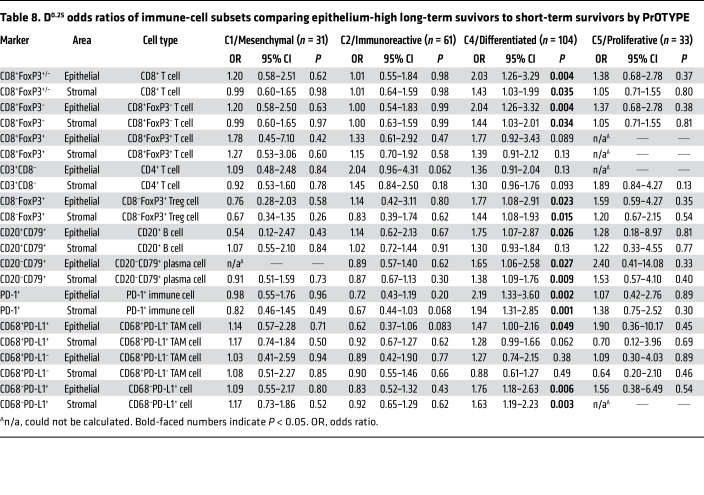
D^0.25^ odds ratios of immune-cell subsets comparing epithelium-high long-term suvivors to short-term survivors by PrOTYPE

## Version 1 10/29/2024

In-Press Preview

## Version 2 12/16/2024

Electronic publication
