# Immunological and molecular features of the tumor microenvironment of long-term survivors of ovarian cancer

**DOI:** 10.1172/JCI179501

**Published:** 2024-10-29

**Authors:** Brad H. Nelson, Phineas Hamilton, Minh Tung Phung, Katy Milne, Bronwyn Harris, Shelby Thornton, Donald Stevens, Shreena Kalaria, Karanvir Singh, Céline M. Laumont, Elena Moss, Aliya Alimujiang, Nicola S. Meagher, Adelyn Bolithon, Sian Fereday, Catherine J. Kennedy, Joy Hendley, Dinuka Ariyaratne, Kathryn Alsop, Nadia Traficante for the Australian Ovarian Cancer Study Group, Ellen L. Goode, Anthony Karnezis, Hui Shen, Jean Richardson, Cindy McKinnon Deurloo, Anne Chase, Bronwyn Grout, Jen Doherty, Holly R. Harris, Kara L. Cushing-Haugen, Michael Anglesio, Karolin Heinze, David Huntsman, Aline Talhouk, Gillian E. Hanley, Jennifer Alsop, Merce Jiminez-Linan, Paul Pharoah, Jessica Boros, Alison H. Brand, Paul R Harnett, Raghwa Sharma, Jonathan L. Hecht, Naoko Sasamoto, Kathryn L. Terry, Beth Karlan, Jenny Lester, Michael E. Carney, Marc T. Goodman, Brenda Y. Hernandez, Lynne R. Wilkens, Sabine Behrens, Renée Turzanski Fortner, Peter A. Fasching, Christiani Bisinotto, Francisco José Candido dos Reis, Prafull Ghatage, Martin Köbel, Esther Elishaev, Francesmary Modugno, Linda Cook, Nhu Le, Aleksandra Gentry-Maharaj, Usha Menon, María J. García, Cristina Rodriguez-Antona, Kyo Farrington, Linda E. Kelemen, Stefan Kommoss, Annette Staebler, Dale W. Garsed, James D. Brenton, Anna M. Piskorz, David D. L. Bowtell, Anna DeFazio, Susan J. Ramus, Malcolm C. Pike, Celeste Leigh Pearce

**Affiliations:** 1 Deeley Research Centre, BC Cancer, Victoria, BC, Canada; 2 Department of Biochemistry and Microbiology, University of Victoria, Victoria, BC, Canada; 3 Department of Medical Genetics, University of British Columbia, Vancouver, BC, Canada; 4 School of Public Health, University of Michigan, Ann Arbor, MI, USA; 5 Department of Population Health Sciences, School of Medicine and Public Health, University of Wisconsin-Madison, Madison, WI, USA; 6 School of Clinical Medicine, UNSW Medicine and Health, University of NSW Sydney, Sydney, NSW, Australia; 7 The Daffodil Centre, The University of Sydney, a joint venture with Cancer Council NSW, Sydney, NSW, Australia; 8 Adult Cancer Program, Lowy Cancer Research Centre, University of NSW Sydney, Sydney, NSW, Australia; 9 Peter MacCallum Cancer Centre, Melbourne, VIC, Australia; 10 Sir Peter MacCallum Department of Oncology, The University of Melbourne, Parkville, VIC, Australia; 11 Centre for Cancer Research, The Westmead Institute for Medical Research, Sydney, NSW, Australia; 12 Department of Gynaecological Oncology, Westmead Hospital, Sydney, NSW, Australia; 13 Faculty of Medicine and Health, The University of Sydney, Sydney, NSW, Australia; 14 Division of Epidemiology, Department of Quantitative Health Sciences, Mayo Clinic, Rochester, MN, USA; 15 Department of Pathology, University of California Davis School of Medicine, Sacramento, CA, USA; 16 Van Andel Institute, Grand Rapids, MI, USA; 17 Department of Population and Public Health Sciences, Keck School of Medicine, University of Southern California, Los Angeles, CA, USA; 18 Patient advocate; 19 Huntsman Cancer Institute, Department of Population Health Sciences, University of Utah, Salt Lake City, UT, USA; 20 Program in Epidemiology, Division of Public Health Sciences, Fred Hutchinson Cancer Center, Seattle, WA, USA; 21 Department of Epidemiology, University of Washington, Seattle, WA, USA; 22 Department of Obstetrics and Gynecology, University of British Columbia, Vancouver, BC, Canada; 23 British Columbia’s Gynecological Cancer Research Team (OVCARE), University of British Columbia, BC Cancer, and Vancouver General Hospital, Vancouver, BC, Canada; 24 Department of Molecular Oncology, BC Cancer Research Centre, Vancouver, BC, Canada; 25 Centre for Cancer Genetic Epidemiology, Department of Oncology, University of Cambridge, Cambridge, UK; 26 Department of Histopathology, Addenbrooke’s Hospital, Cambridge, UK; 27 Department of Computational Biomedicine, Cedars-Sinai Medical Center, West Hollywood, CA, USA; 28 Crown Princess Mary Cancer Centre, Westmead Hospital, Sydney, NSW, Australia; 29 Tissue Pathology and Diagnostic Oncology, NSW Health Pathology, Westmead Hospital, Sydney, NSW, Australia; 30 Western Sydney University, Sydney, NSW, Australia; 31 Department of Pathology, Beth Israel Deaconess Medical Center and Harvard Medical School, Boston, MA, USA; 32 Obstetrics and Gynecology Epidemiology Center, Department of Obstetrics and Gynecology, Brigham and Women’s Hospital and Harvard Medical School, Boston, MA, USA; 33 Department of Epidemiology, Harvard T.H. Chan School of Public Health, Boston, MA, USA; 34 David Geffen School of Medicine, Department of Obstetrics and Gynecology, University of California at Los Angeles, Los Angeles, CA, USA; 35 Department of Obstetrics and Gynecology, John A. Burns School of Medicine University of Hawaii, Honolulu, HI, USA; 36 Cancer Prevention and Control Program, Cedars-Sinai Cancer Center, Cedars-Sinai Medical Center, Los Angeles, CA, USA; 37 University of Hawaii Cancer Center, Honolulu, HI, USA; 38 Division of Cancer Epidemiology, German Cancer Research Center (DKFZ), Heidelberg, Germany; 39 Department of Research, Cancer Registry of Norway, Oslo, Norway; 40 Department of Gynecology and Obstetrics, Comprehensive Cancer Center Erlangen-EMN, Friedrich-Alexander University Erlangen-Nuremberg, University Hospital Erlangen, Erlangen, Germany; 41 Department of Gynecology and Obstetrics, Ribeirão Preto Medical School, University of São Paulo, Ribeirão Preto, Brazil; 42 Department of Oncology, Division of Gynecologic Oncology, Cumming School of Medicine, University of Calgary, Calgary, AB, Canada; 43 Department of Pathology and Laboratory Medicine, University of Calgary, Foothills Medical Center, Calgary, AB, Canada; 44 Department of Pathology, University of Pittsburgh School of Medicine, Pittsburgh, PA, USA; 45 Department of Epidemiology, University of Pittsburgh School of Public Health, Pittsburgh, PA, USA; 46 Division of Gynecologic Oncology, Department of Obstetrics, Gynecology and Reproductive Sciences, University of Pittsburgh School of Medicine, Pittsburgh, PA, USA; 47 Women’s Cancer Research Center, Magee-Womens Research Institute and Hillman Cancer Center, Pittsburgh, PA, USA; 48 Epidemiology, School of Public Health, University of Colorado, Aurora, CO, USA; 49 Community Health Sciences, University of Calgary, Calgary, AB, Canada; 50 Cancer Control Research, BC Cancer Agency, Vancouver, BC, Canada; 51 MRC Clinical Trials Unit, Institute of Clinical Trials and Methodology, University College London, London, UK; 52 Department of Women’s Cancer, Elizabeth Garrett Anderson Institute for Women’s Health, University College London, London, UK; 53 Cancer Biology Department, Sols-Morreale Biomedical Research Institute (IIBM), CSIC UAM, Madrid, Spain; 54 Hereditary Endocrine Cancer Group, Spanish National Cancer Research Center (CNIO), Madrid, Spain; 55 Centre for Biomedical Network Research on Rare Diseases (CIBERER), Instituto de Salud Carlos III, Madrid, Spain; 56 Division of Acute Disease Epidemiology, South Carolina Department of Health and Environmental Control, Columbia, SC, USA; 57 Department of Women’s Health, Tuebingen University Hospital, Tuebingen, Germany; 58 Institute of Pathology and Neuropathology, Tuebingen University Hospital, Tuebingen, Germany; 59 Cancer Research UK Cambridge Institute, University of Cambridge, Cambridge, UK; 60 Department of Epidemiology and Biostatistics, Memorial Sloan Kettering Cancer Center, New York, NY, USA

## Abstract

**Background:**

Despite an overall poor prognosis, about 15% of patients with advanced-stage tubo-ovarian high-grade serous carcinoma (HGSC) survive ten or more years after standard treatment.

**Methods:**

We evaluated the tumor microenvironment of this exceptional, understudied group using a large international cohort enriched for long-term survivors (LTS; 10+ years; *n* = 374) compared to medium-term (MTS; 5–7.99 years; *n* = 433) and short-term survivors (STS; 2–4.99 years; *n* = 416). Primary tumor samples were immunostained and scored for intra-epithelial and intra-stromal densities of 10 immune-cell subsets (including T cells, B cells, plasma cells, myeloid cells, PD-1^+^ cells, and PD-L1^+^ cells) and epithelial content.

**Results:**

Positive associations with LTS compared to STS were seen for 9/10 immune-cell subsets. In particular, the combination of intra-epithelial CD8^+^ T cells and intra-stromal B cells showed near five-fold increased odds of LTS compared to STS. All of these associations were stronger in tumors with high epithelial content and/or the C4/Differentiated molecular subtype, despite immune-cell densities generally being higher in tumors with low epithelial content and/or the C2/Immunoreactive molecular subtype.

**Conclusions:**

The tumor microenvironment of HGSC long-term survivors is distinguished by the intersection of T and B cell co-infiltration, high epithelial content and C4/Differentiated molecular subtype, features which may inspire new approaches to immunotherapy.

## Introduction

Although advanced-stage tubo-ovarian high-grade serous carcinoma (HGSC) remains a challenging, largely incurable disease, about 15% of patients survive ten or more years after diagnosis,(1) providing an opportunity to define the features associated with long-term survival. Progress on this front has been hampered by the relative rarity of such cases combined with the need for large long-term research programs with biospecimen banking and systematic clinical follow-up to accrue sufficient cases.(2) For example, in the HGSC patient cohort of The Cancer Genome Atlas (TCGA), only 1% (4/405) of cases had an overall survival (OS) of 10+ years.(3) To address these challenges, the Multidisciplinary Ovarian Cancer Outcomes Group (MOCOG) was formed to identify the immunologic, genomic and epidemiological factors associated with long-term survival in HGSC. We report here our findings regarding the immune tumor microenvironment (TME) of 374 long-term survivors (LTS; 10+ year) compared to 433 medium-term survivors (MTS; 5-7.99 years) and 416 short-term survivors (STS; 2-4.99 years). Unusually poor survivors (<2 years) were not included in the study because these patients may have had primary platinum-resistant disease or exceptionally late diagnosis.

The presence at diagnosis of tumor-infiltrating lymphocytes (TILs), in particular CD8+ T cells, is associated with improved survival from HGSC.(4–7) Other TIL subsets associated with favorable prognosis include CD4+ T cells, CD20+ B cells, and plasma cells.(7–14) Expression of PD-1 by TILs and expression of its ligand (PD-L1) by tumor and myeloid cells (15–17) is also a favorable prognostic feature in HGSC, likely reflecting the role of this pathway in active anti-tumor immunity.(18) However, the immune cell composition associated with exceptional patient survival has not been defined and represents a critical knowledge gap on the path to developing more effective immunotherapies.

In contrast to TILs, cancer-associated fibroblasts (CAFs) and high stromal content in general are negative prognostic factors in HGSC and other cancers.(19–27) Indeed, numerous studies have identified a "C1/Mesenchymal" (C1/MES) subtype of HGSC that is enriched in CAFs and associated with poor prognosis.(3, 28, 29) However, the methods used to define stroma vary widely between studies (30, 31) and generally overlook the fact that TILs can dominate the stromal compartment of immunologically active tumors.(11) Thus, the relationship between tumor stroma and anti-tumor immunity is complex, and the relative influence of these factors on long-term survival is unknown.

Gene expression profiling studies have generally converged on four biologically relevant molecular subtypes of HGSC (3, 15, 29–32). As mentioned, C1/MES tumors have the highest stromal and CAF content. C2/Immunoreactive (C2/IMM) tumors are enriched for T cells, B cells and other immune cells. C4/Differentiated (C4/DIF) tumors express higher levels of MUC16 and other epithelial gene products and have moderate levels of immune cells. C5/Proliferative (C5/PRO) tumors express gene products associated with stem cells, cell cycle, and epithelial-to-mesenchymal transition and have negligible immune-cell infiltration. The C1/MES subtype has been associated with poor survival in most studies, whereas the C2/IMM subtype and, in many cases, the C4/DIF subtype have been associated with prolonged survival.(3, 15, 29–33)

Here we report a systematic analysis of TIL subsets, epithelial/stromal content and molecular subtype in the MOCOG cohort, which revealed what we believe to be a novel intersection between these factors and long-term survival in HGSC.

## Results

### Diverse immune-cell subsets are associated with long-term survival

**Figure 1:**
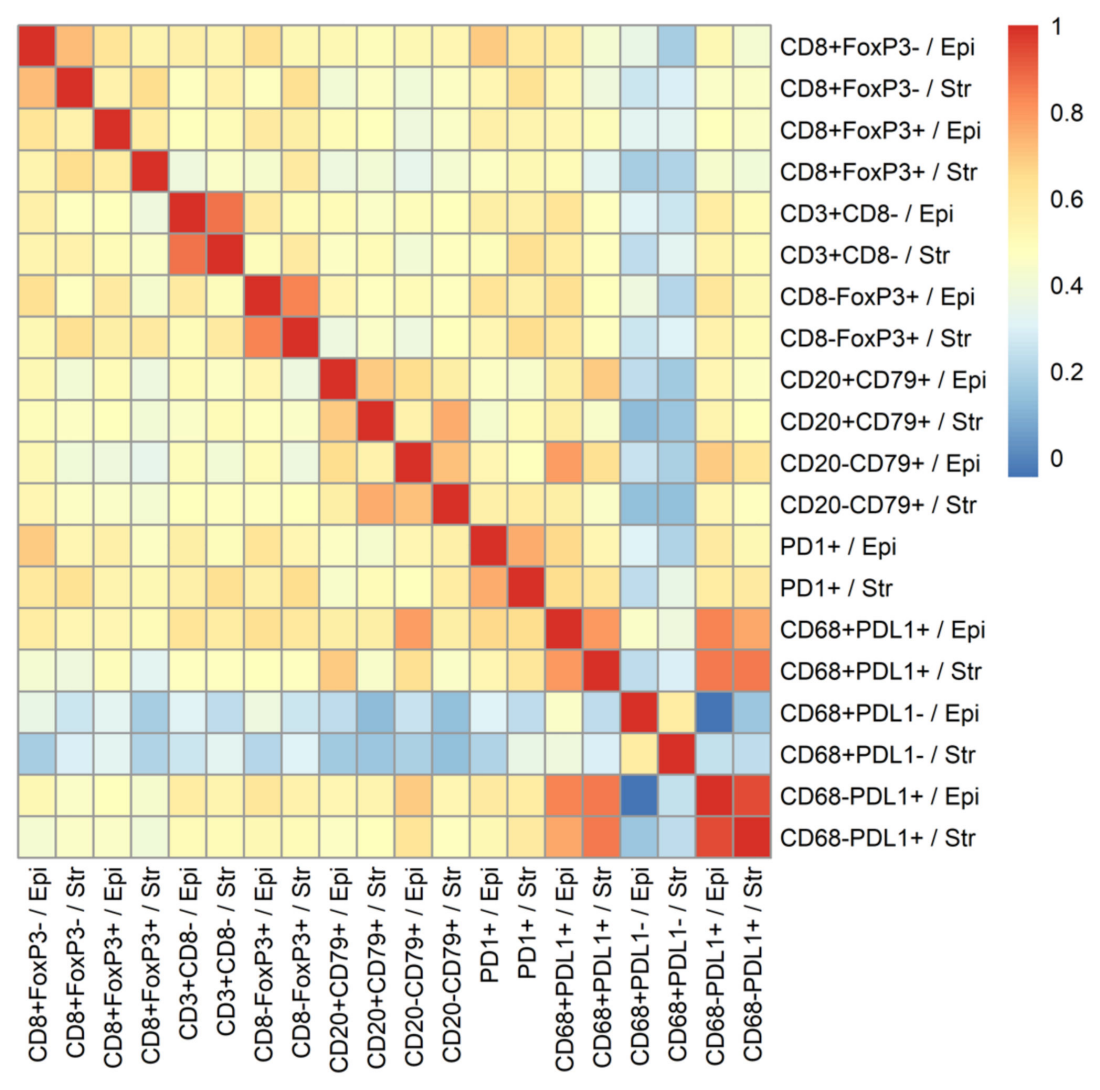
Heatmap showing pairwise Spearman correlations between immune cell subsets. The color scale indicates the strength of the correlation between densities with red indicating high positive correlation. Epi = intra-epithelial location, Str = intra-stromal location of immune cells

Table 1 and Supplementary Table 1 summarize key features of the 1,223 evaluated participants (374 LTS, 433 MTS, 416 STS). Multi-color immunohistochemistry (IHC) or immunofluorescence (IF) was used to determine the densities of 10 immune-cell subsets in the epithelial and stromal regions of the tumors; an 11th immune-cell subset was generated by summing CD8+FoxP3- and CD8+FoxP3+ T cell counts to create a single "CD8+ T cell" measure (Table 2). Immune cells were generally more abundant in stromal than epithelial tumor regions. Most immune-cell subsets were positively correlated (Figure 1), the only exception being CD68+PD-L1- TAMs, which showed no association with other immune-cell types.

**Figure 2:**
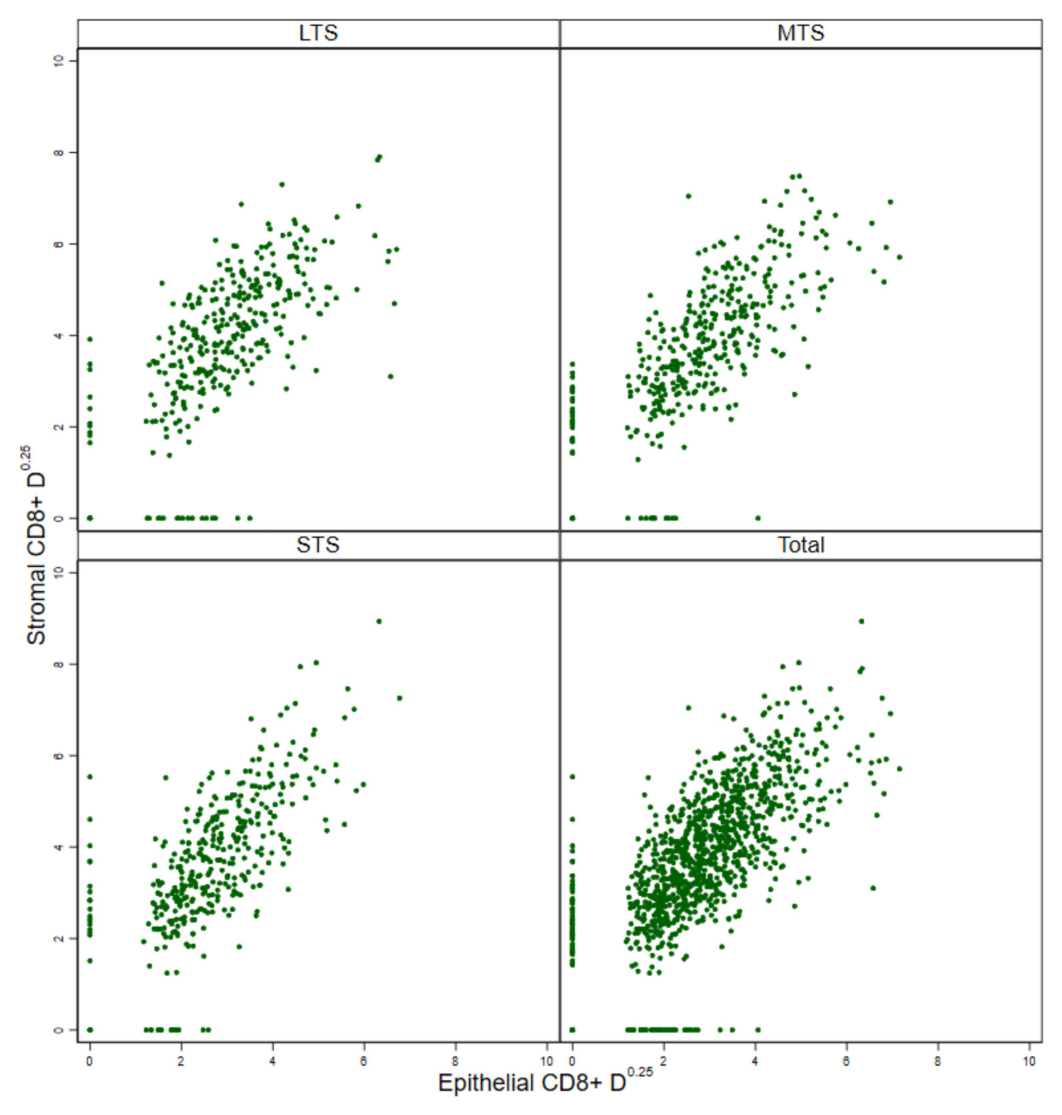
Intra-stromal versus intra-epithelial CD8+ TIL densities (cells/mm^2^) in tumors from all participants and from the STS, MTS and LTS subgroups. The relationship of the intra-stromal CD8+ density values to the intra-epithelial CD8+ density values showed no differences between STS and LTS (p=0.60).

In HGSC and other cancers, an "immune excluded" TIL pattern has been described in which T cells are predominantly restricted to tumor stroma rather than epithelium.(34) To assess this, we plotted intra-epithelial versus intra-stromal CD8+ T cell densities (Figure 2). Although a small number of tumors were devoid of intra-epithelial CD8+ cells or intra-stromal CD8+ cells (which was likely attributable to the small size of the TMA cores), we found no evidence of a distinct subgroup of tumors with substantial intra-stromal values and negligible intra-epithelial values overall or individually for the LTS, MTS and STS survival groups (Figure 2). Thus, immune exclusion was not evident in our cohort, and we did not consider this or other spatial patterns in subsequent analyses.

Table 3 shows the ORs (using D^0.25^; see Methods sections, Statistics) comparing LTS to STS, LTS to MTS, and MTS to STS for all immune-cell subsets in epithelium and in stroma. In the LTS to STS comparison, with the exception of CD68+PD-L1- TAMS, all immune-cell subsets were substantially more abundant in LTS than STS in the epithelium and/or stroma. Within the epithelial compartment, we found highly statistically significant associations with survival for intra-epithelial CD8+ T cells (including both FoxP3- and FoxP3+ subsets), PD-1+ immune cells, CD68-PD-L1+ cells (presumptive PD-L1+ tumor cells) and B cells. Within the stromal compartment, the most significant associations (p≤0.002) were seen for CD8+FoxP3+ T cells, PD-1+ cells, CD20-CD79+ plasma cells, and CD68-PD-L1+ cells. Intra-epithelial CD8+FoxP3+ T cells and PD-1+ cells had the strongest associations based on OR magnitude. Most immune marker associations for MTS versus STS were substantially weaker than those for LTS versus STS (Table 3). Results restricted to participants whose samples were known to be adnexal (data not shown) or who were known to have received primary cytoreductive surgery (PCS) (Supplementary Table 2) were not materially different from our main results (data not shown).

### Prognostic effects of TILs are strongest in tumors with high epithelial content

**Figure 3:**
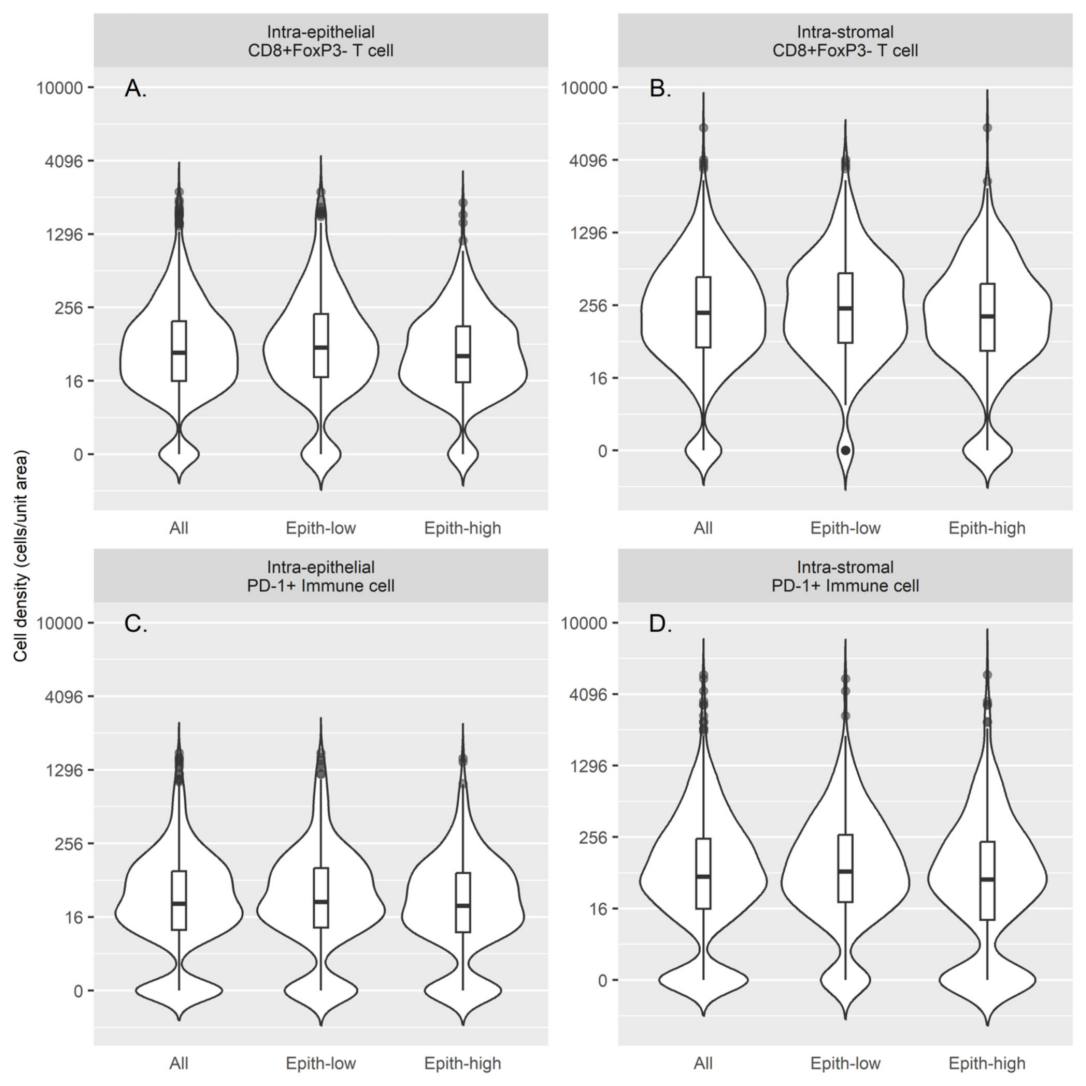
Violin plots comparing the densities of immune cell subsets in epithelium-high versus –low tumors. **A**. Density of intra-epithelial CD8+FoxP3- T cells in all tumors, epithelium-low tumors, and epithelium-high tumors. **B**. Intra-stromal CD8+FoxP3- T cells. **C**. Intra-epithelial PD-1+ immune cells. **D**. Intra-stromal PD-1+ immune cells. Embedded box plots indicate median (horizontal line), quartile (box edges) and outliers (points).

To investigate whether epithelial-to-stromal content influenced the magnitude or prognostic significance of immune-cell infiltrates, patients were stratified into epithelium-high versus -low groups (see Supplementary Methods, *Statistics* details). While epithelial content showed no significant association with LTS, MTS or STS survival group. The densities of almost all immune-cell subsets (both intra-epithelial and intra-stromal) were higher in the epithelium-low group, with the exception of intra-epithelial CD68+PD-L1+ TAMs and intra-epithelial and intra-stromal CD68+PD-L1- TAMs (Table 2). For example, Figure 3 compares the densities of CD8+FoxP3- T cells and PD-1+ cells in epithelium-high versus epithelium-low tumors.

**Figure 4:**
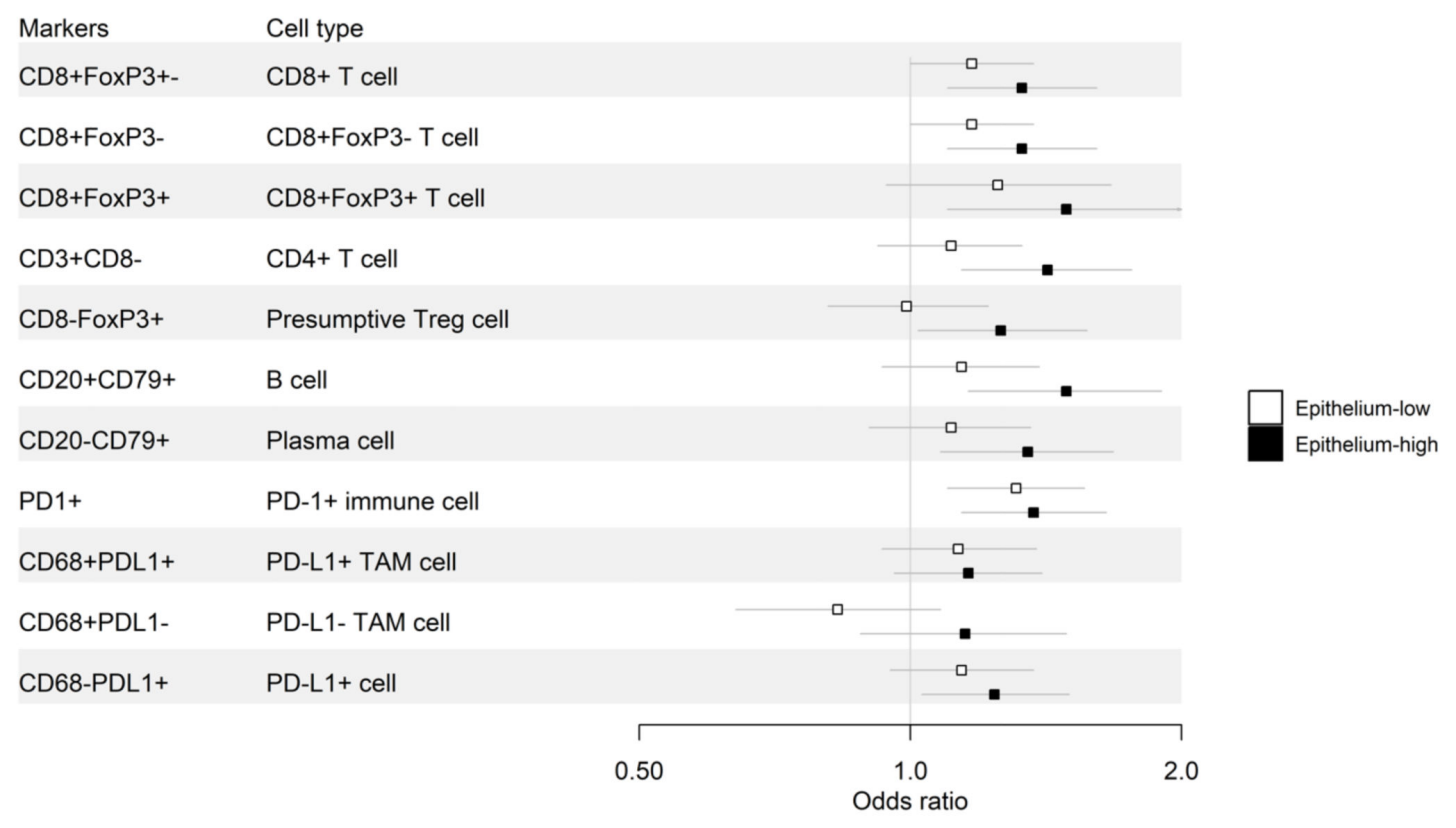
Forest plot of the odds ratios and 95% confidence intervals of LTS compared to STS for intra-epithelial immune cell subsets stratified by epithelium-high versus epithelium-low tumors.

We next evaluated the impact of epithelial content on the prognostic effects of immune markers. Table 4, Figure 4, and Supplementary Figure 1 show a comparison of the LTS and STS groups. Immune marker associations were markedly stronger in the epithelium-high group compared to the epithelium-low group. In the epithelium-high group, 9/11 intra-epithelial (Table 4, Figure 4) and 10/11 intra-stromal (Supplementary Figure 1) immune-cell subsets showed a statistically significant association with survival group (LTS versus STS); this included all T cell, B cell and plasma cell subsets. By contrast, in the epithelium-low group, only intra-epithelial CD8+ T cells and PD-1+ cells were statistically significantly associated with survival group. Thus, the association of TILs with LTS was largely restricted to tumors with high epithelial content, even though TIL densities were generally higher in epithelium-low tumors (Figure 3). Similar results were obtained when quartile (see Methods section, Statistics) OR results were used to compare LTS to STS by epithelium group (Supplementary Table 3). In the MTS versus STS comparison, the influence of epithelial content was evident but less striking (Supplementary Table 4).

Further analyses of immune-cell subsets were performed in two population-based HGSC cohorts: COEUR (n=981) (35) and OOU (n=192) (36); the latter had previously been stained to detect T cell phenotypes (including CD39+ and CD103+ T cells) that were not evaluated in the MOCOG or COEUR panels. Similar to the MOCOG findings, these analyses showed that the prognostic effects of most TIL subsets were restricted to epithelium-high tumors, despite immune-cell densities being generally higher in epithelium-low tumors (Supplementary Material and Supplementary Tables 5, 6, 7 and 8).

### Intra-stromal B cells complement the prognostic effect of intra-epithelial CD8+ T cells

We investigated which combination of immune cells best predicted outcome in the epithelium-high group. Because of the well-accepted importance of intra-epithelial CD8+ T cells, we included this marker in the analysis *a priori*. After accounting for intra-epithelial CD8+ T cells, intra-stromal B cells were the only other immune-cell subset that distinguished LTS and STS (Supplementary Table 9). PD-1+ immune cells and CD8+ T cells densities were highly correlated, and interchanging PD-1+ and CD8+ left the conclusions unchanged. In a joint effects model (Table 5), an OR of 4.87 was observed for the highest quartile for intra-epithelial CD8+ T cells and non-zero for intra-stromal B cells (p<0.001, with no statistical interaction between these two markers [p>0.05]).

### The prognostic effects of immune cells depend on molecular subtype

PrOTYPE molecular subtyping data was available for 217 LTS, 251 MTS and 226 STS MOCOG cases.(33) Across all three groups combined, the molecular subtypes were distributed as: C1/MES (24%), C2/IMM (27%), C4/DIF (33%) and C5/PRO (16%). There was no statistically significant association between molecular subtype and survival group. Consistent with prior reports,(33, 37) epithelial content was highest in the C4/DIF subtype (median, 75%), lowest in the C1/MES subtype (median, 57%), and intermediate in the C2/IMM (median, 67%) and C5/PRO subtypes (median, 69%), (p<0.001).

**Figure 5:**
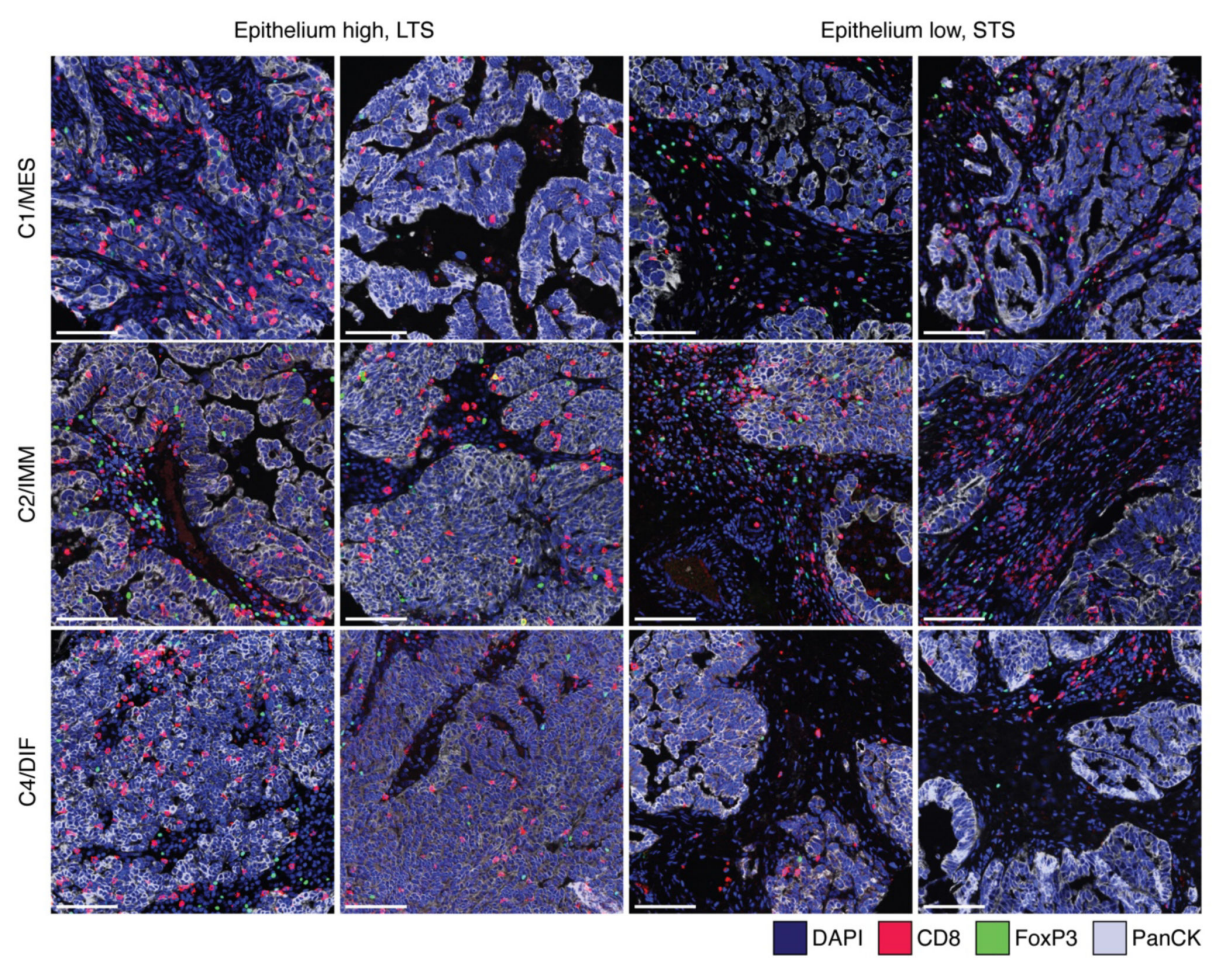
Multiplex immunofluorescence images showing CD8+ (red) and FoxP3+ (green) TILs in epithelium-high versus -low tumors, further stratified by molecular subtype (C1/MES, C2/IMM and C4/DIF); two representative examples of each subgroup are shown. Tumor cells are highlighted by pan-cytokeratin staining (light gray). DAPI staining (blue) detects all cell nuclei. Scale bars indicate 100 mm.

As expected, the C2/IMM subtype had the highest median densities of all intra-epithelial and intra-stromal immune-cell subsets, followed in order by the C1/MES, C4/DIF and C5/PRO subtypes (Table 6 and Figure 5). For most immune-cell subsets, the ratio of intra-stromal to intra-epithelial cell densities did not vary substantially between molecular subtypes.

**Figure 6:**
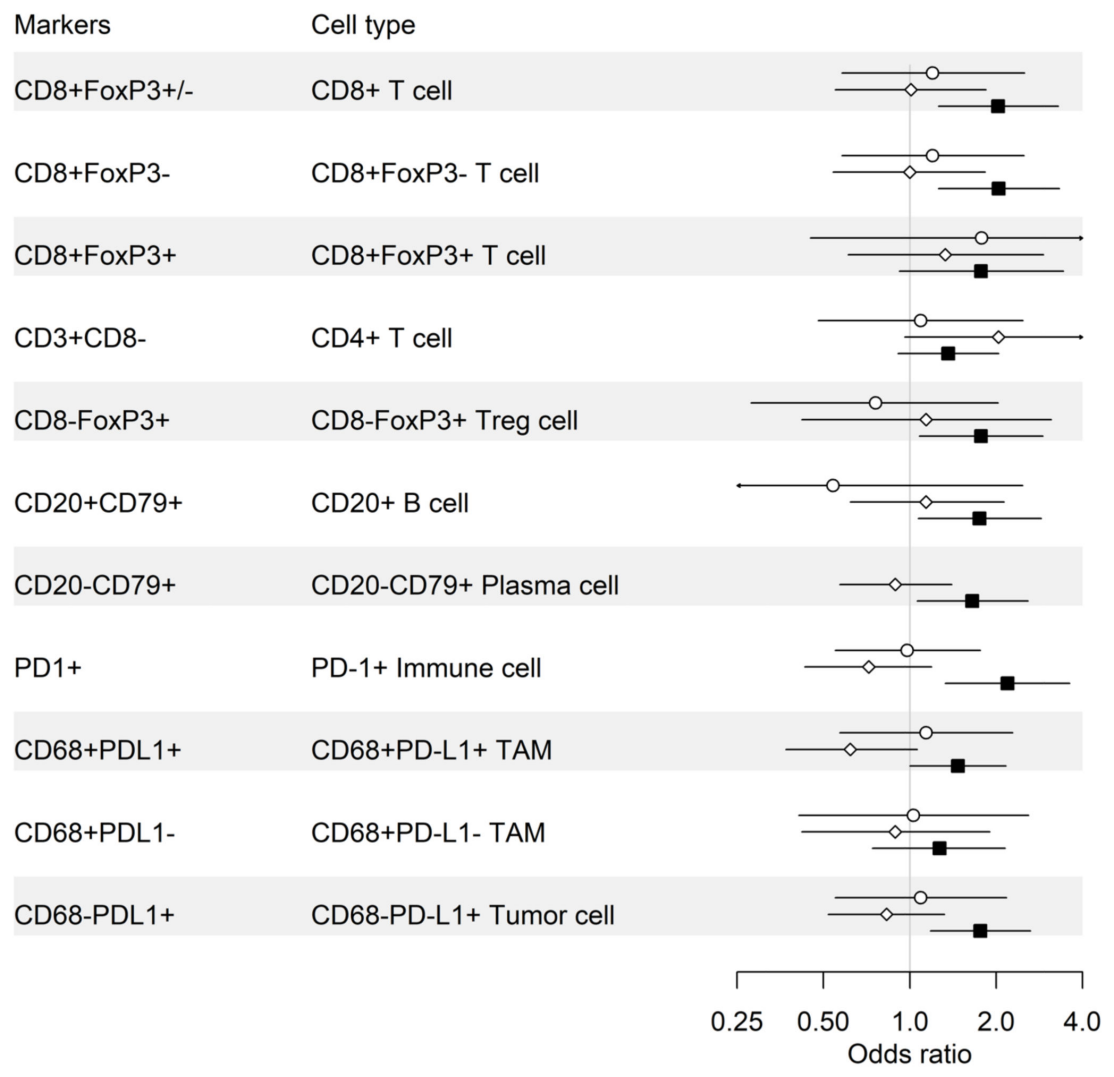
Forest plot of the odds ratios and 95% confidence intervals of LTS compared to STS of intra-epithelial immune-cell subsets for the C1/MES, C2/IMM and C4/DIF molecular subtypes in epithelium-high cases. Plasma cell results for the C1/MES subtype could not be calculated. The C5/PRO subtype is not presented as several could not be calculated. * indicates the p-value for heterogeneity across the subtypes is <0.05.

Unexpectedly, the association between immune cells and LTS was near exclusive (with only one exception) to the C4/DIF subtype. Statistically significant positive associations were seen for 5/11 intra-epithelial and 4/11 intra-stromal cell subsets (Table 7; Figure 6; Supplementary Figure 2), despite the fact that C4/DIF tumors ranked third among the molecular subtypes with respect to the densities of 19/22 immune-cell subsets (Table 6). In contrast, the C2/IMM molecular subtype had the highest median levels of all intra-epithelial and intra-stromal immune-cell subsets (Table 6), yet only intra-epithelial CD8+FoxP3+ T cells showed a statistically significant association with LTS within this molecular subtype (Table 7).

To investigate whether these C4/DIF results reflected the influence of tumor epithelium, we restricted the comparison between LTS and STS to epithelium-high tumors (Table 8). As expected,(33, 37) a higher proportion of C4/DIF tumors were epithelium-high (n=104/155, 67%) compared to C1/MES (n=31/109, 28%), C2/IMM (n=61/108, 56%) and C5/PRO tumors (n=33/71, 46%) (Tables 7 and 8). Strikingly, within epithelium-high tumors, the prognostic significance of immune cells was exclusive to the C4/DIF subtype, with 8/11 intra-epithelial and 6/11 intra-stromal subsets showing a statistically significant association with LTS. There were no statistically significant associations within the other molecular subtypes (Table 8).

## Discussion

The immunological and microenvironmental features associated with LTS had remained largely undefined in HGSC owing to the rarity of such patients and the paucity of biospecimens with sufficient follow-up data. To address this critical gap, we assembled a large international cohort comprised of similar numbers of STS, MTS and LTS patients. The TME of LTS cases was distinguished by co-infiltration of T cells, B cells, and plasma cells, along with upregulation of the PD-1/PD-L1 pathway. Remarkably, these prognostic associations were almost entirely restricted to tumors with high-epithelial content and the C4/DIF subtype, factors that have not previously been implicated as influencing the prognostic effects of TILs and TAMs. These findings were not attributable to higher TIL or TAM densities in epithelium-high or C4/DIF tumors, indicating they instead reflect other biological features associated with these tumor subgroups. Our findings suggest that immunotherapies for HGSC should be designed to engage not only T cells but also the B cell and myeloid cell lineages. They further suggest that the immunobiology of epithelium-high, C4/DIF tumors warrants further study to understand their apparent enhanced susceptibility to immune-based control mechanisms.

In addition to TILs, intra-epithelial CD68+PD-L1+TAMs showed an association with LTS, consistent with prior reports.(16, 38) While PD-L1 has a well-established immunosuppressive role, it is also an indicator of active TIL responses, which may explain the favorable prognostic association.(16) Apart from PD-L1, TAMs can suppress anti-tumor immunity through a variety of other mechanisms, and future studies are warranted to assess the influence of these additional factors on long-term outcomes.(39)

In principle, our finding that the prognostic effect of immune cells is attenuated in epithelium-low tumors could reflect the immunosuppressive properties of CAF-rich tumor stroma.(19–27, 40) In particular, the C1/MES subtype of HGSC is characterized by desmoplastic stroma and, reportedly, a preferential localization of T cells in tumor stroma.(28, 29) This "immune-excluded" pattern has also been reported in other cancers, although a consensus definition has not been reached.(41) Despite these prior reports, we saw no evidence of a distinct immune-excluded subgroup of tumors in the STS, MTS or LTS groups. Moreover, we found that epithelium-low tumors had higher average densities of almost all immune-cell subsets (except CD68+PD-L1- TAMs) in both the epithelial and stromal compartments. Therefore, while CAFs and/or other stromal elements could explain the blunted prognostic effect of TIL in epithelium-low tumors, immune exclusion does not appear to be the underlying mechanism. Notably, McGregor and colleagues found that epithelial content had a minimal influence on TIL densities and activation profiles in HGSC; instead, epithelium-high tumors showed evidence of increased activation of cross-presenting dendritic cells, which could activate tumor-specific CD8+ T cells.(42) Thus, further studies are warranted to assess the functional status of TILs in epithelium-high versus -low tumors.

Our finding that the prognostic effect of TILs is restricted to epithelium-high tumors has implications for TIL scoring in the HGSC setting. Several studies have scored cases based on the absolute number of intra-epithelial T cells per field;(4, 7) presumably epithelium-high tumors would score higher with this approach, which could inadvertently amplify the prognostic effect of TILs. Furthermore, exclusion of tumor cores with low epithelial content would enrich for epithelium-high tumors, again amplifying the prognostic significance of TILs. Thus, epithelial content is an important variable in immune-related prognostic studies.

To our knowledge, this is the first study to assess the prognostic significance of TILs across the four molecular subtypes of HGSC, which led to the unexpected finding that the association between TILs and LTS is almost entirely restricted to the C4/DIF subtype. A relatively large yet understudied group, C4/DIF tumors were initially reported to have only moderate TIL levels yet favorable prognostic significance on a par with the C2/IMM subtype.(29) The TCGA study named this the "Differentiated" subtype based on higher expression of epithelial markers (e.g., MUC16 and MUC1), which was suggested to reflect a more mature stage of development.(3) Wang and colleagues identified within the C4/DIF subtype a fifth molecular subtype they called "anti-mesenchymal" owing to the downregulation of genes associated with the C1/MES subtype.(43) This novel subtype, which could represent the epithelium-high C4/DIF subset reported here, was associated with the longest survival among the five subtypes.(43) Talhouk and colleagues also found that C4/DIF tumors were associated with high patient survival rates, equivalent to C2/IMM tumors.(33) Moreover, C4/DIF tumors were more more likely to have an adnexal location and exhibited high tumor purity, equivalent to C5/PRO tumors.(33) Intriguingly, C4/DIF tumors were also associated with a younger age at diagnosis,(33) which was also reported by Tothill and colleagues.(29)

Waldron and colleagues too found that C4/DIF tumors were associated with younger patient age and higher tumor purity.(37) They further showed that C4/DIF tumors had lower ploidy, lower copy number variation, and lower subclonality compared to the other molecular subtypes, consistent with a lower number of genome doublings.(37) In contrast, C5/PRO tumors showed a higher number of gene amplifications, higher ploidy, and increased frequency of genome duplication. Single-cell RNA-sequencing (scRNA-seq) revealed that the majority of tumor cells (as opposed to other cell types in the admixture) exhibited a C4/DIF transcriptional signature, with the remaining tumor cells being assigned a C5/PRO signature,(37) a finding which is consistent with other scRNA-seq studies.(44, 45) They proposed that C4/DIF and C5/PRO tumors represent different ends of an evolutionary time scale – more recently arising tumors versus older tumors, respectively – and that the C1/MES and C2/IMM subtypes are derivatives whose signatures merely reflect the presence of mesenchymal and immune cells.(37) If this hypothesis is correct, the favorable immunological and prognostic associations seen with C4/DIF tumors could be attributable to evolutionarily "younger" tumors, earlier diagnosis, age at diagnosis, and/or lower intratumoral heterogeneity, features that have previously been linked to improved prognosis (46–48) and could plausibly facilitate more effective anti-tumor immunity.(46–48)

Finally, the prognostic benefits of C4/DIF tumors could also have an immunological explanation. Applying scRNA-seq to primary HGSC samples, Olbrecht and colleagues found that tumor cells mapping to the C4/DIF subtype were enriched for transcripts reflecting interferon signaling, suggesting exposure to an active immune response.(45) Owing to their more differentiated state, C4/DIF tumors could also have higher densities of antigenic epitopes for recognition by CD8+ T cells; for example, peptides derived from MUC16 are predominant components of the MHC class I and class II peptide landscape in HGSC.(49) Thus, C4/DIF tumor cells and tumors exhibit distinct clinical, histological, transcriptional, genomic, and immunological features that warrant further study as potential determinants of patient survival.

C2/IMM tumors had the highest densities of all immune-cell subsets; however, with the exception of CD8+FoxP3+ T cells, immune cells showed no statistically significant association with LTS within this molecular subtype. This finding was unexpected given the well-established prognostic benefit of TILs in HGSC,(50) including the report of a positive dose-response association between intra-epithelial CD8+ T cells and survival.(4) This could reflect a dynamic range issue wherein most C2/IMM cases have immune cell densities that exceed the threshold required to promote LTS. If so, however, it is unclear why the C2/IMM subtype did not show a stronger association with LTS relative to the other molecular subtypes. Another possible explanation is that prior studies have not been as highly enriched for LTS cases as the present study. For example, the TCGA (30) and PrOTYPE (33) studies had only 1% and 8.4% LTS cases, respectively, and may have been underpowered to detect the combined effects of immune cell infiltrates and C4/DIF subtype on LTS shown here.

The substantial prognostic effect of B cells and plasma cells shown here aligns with prior reports in HGSC (8, 11–14, 51) and the emerging appreciation for the role of the B-cell compartment in anti-tumor immunity in other cancers.(52, 53) Furthermore, in a recent genomic/transcriptomic study of HGSC, we found that plasma cell gene signatures were independent predictors of LTS along with *BRCA2*-type homologous recombination deficiency, *PCNA* expression, and residual disease.(10) It was recently proposed that the prognostic benefit of plasma cells may be impaired when they co-localize with CAFs, (54) which fits with our finding that the prognostic benefit of plasma cells and B cells is strongest in epithelium-high (presumably CAF low) tumors. With respect to possible effector mechanisms, B cells and plasma cells can potentially enhance T cell responses by helping to organize lymphoid aggregates, including tertiary lymphoid structures, and by serving as antigen-presenting cells.(52, 53) Indeed, a hallmark of "exhausted/dysfunctional" tumor-infiltrating CD8 T cells in HGSC and other cancers is expression of the B cell-recruiting chemokine CXCL13,(36, 55–57) suggesting that T cells are programmed to solicit B cell help in the face of chronic antigen stimulation. Accordingly, we found that tumors containing dense intra-epithelial CD8+ T cells (highest quartile) combined with intra-stromal CD20+ B cells were associated with an almost five-fold higher likelihood of being LTS versus STS. In addition to T cell-based mechanisms, the antibodies produced by plasma cells in HGSC have been shown to bind tumor antigens (8, 12) enabling them to potentially block the function of their target protein directly and/or engage innate effector mechanisms such as complement-mediated cytotoxicity, antibody-dependent cellular phagocytosis by TAMs, and antibody-dependent cellular cytotoxicity by natural killer and TAM cells.(52, 53) These latter mechanisms could explain the co-associations between CD68+ PD-L1+ TAM cells and various TIL subsets and their marked enrichment in LTS cases.

Our study has several potential limitations. While the use of TMAs from 19 different studies, four continents and a 26-year time frame should increase the generalizability of our findings, it also presented technical challenges related to immunohistological staining and scoring. To mitigate variability in specimen age and quality, we deployed four small, robust panels of markers; this restricted our ability to definitively identify some cell types (e.g., Tregs, plasma cells) and to assess nearest-neighbor relationships between diverse immune-cell subsets in the same tissue section. A related limitation was the use of TMAs instead of whole sections, which reduced our ability to detect prognostically relevant immune aggregates, including tertiary lymphoid structures.(11) TMAs also may not capture the full heterogeneity of TIL and TAM patterns, (58) but this issue would be partly mitigated by our large sample sizes. The use of TMAs is also relevant to our classification of cases into epithelium-high versus -low groups, as the tumor cores on TMAs represent only a small fraction of a patient’s overall tumor burden and are typically punched from areas with the highest epithelial content. Indeed, the cores used for TMA construction were selected by each study’s pathologist, resulting in non-standardized epithelial versus stromal content between studies; however, such variation was mitigated by the large sample size of the MOCOG cohort and the corroborating results obtained from the COEUR and OOU cohorts. A further limitation is that we defined stromal regions by the absence of epithelial features rather than directly staining for markers of CAFs, endothelium or other stromal cell types that can influence prognosis.(19–27) Thus, an important future direction will be to assess immune cells in larger tumor regions using more highly multiplexed methods (which were not available when this study was initiated) that include detection of key stromal cell types. Use of such methods will also enable analysis of the spatial relationships between cell types, which can have a substantial influence on anti-tumor immunity and patient survival.(59–61) It will also be important to validate new findings in independent cohorts, in particular what we believe to be the novel influence of epithelial content and C4/DIF molecular subtype on the prognostic effect of TIL. Finally, our use of a high threshold for LTS (≥10 years) necessitated inclusion of patients predominately from the 2000s; therefore, contemporary treatment regimens (e.g., angiogenesis and PARP inhibitors) were not well represented in the cohort. In future studies, it will be important to determine how these new therapeutic agents modify the relationship between tumor biology and patient survival.

Our findings have implications for the treatment of HGSC. First, they provide further justification for the development of combination immunotherapies that coordinately enhance the orthogonal effector mechanisms used by T cells, B-lineage cells, and myeloid cells.(52, 53) Second, they challenge the notion that immune exclusion is a major barrier in C1/MES tumors given these tumors harbored relatively abundant TILs (similar or higher than C4/DIF tumors) in both the epithelial and stromal compartments. Finally, our work suggests that epithelium-high and/or C4/DIF tumors may represent especially attractive targets for immunotherapy and could help elucidate the critical immune barriers present in other subtypes. In this regard, C4/DIF tumors represent the largest molecular subtype (33.2% of cases in the PrOTYPE study),(33) yet they have received the least investigation from an immunological perspective. By resembling normal epithelium more closely, C4/DIF tumors may be more conducive to immune-mediated control mechanisms. For example, there is growing appreciation of the importance of biomechanical forces in immune surveillance and tumor cell killing.(62) This could provide rationale for combining immunotherapy with pharmaceutical agents that promote tumor cell differentiation and/or a more normal epithelial architecture.(63) Thus, C4/DIF tumors may hold important clues for developing the next generation of immunotherapies for HGSC and related malignancies.

## Methods

### Sex as a biological variable

This study was focused exclusively on HGSC, a disease which affects only biological females.

### Study population and tumor samples

The MOCOG cohort was assembled from studies in Australia, Europe, North America, and Brazil. Each participating study received local ethics review board approval. Specimens were obtained with written informed consent (or a formal waiver of consent) with approval by the relevant ethics review board. Of n=1,298 total tumors, 1,223 were successfully stained and scored (Supplementary Table 1). Patients were diagnosed between 1985 and 2011 with FIGO Stage III/IV ovarian, fallopian tube or primary peritoneal HGSC. Survival groups were defined as LTS (10+ years), MTS (5-7.99 years) and STS (2-4.99 years) from the date of diagnosis, respectively. STS and MTS patients were frequency matched to LTS patients by study, year of diagnosis, and patient age at diagnosis. Supplementary Figure 3 illustrates the study design. Studies constructed their own tissue microarrays (TMAs) from formalin-fixed paraffin-embedded (FFPE) blocks of tumor tissue. TMA cores were 0.6-1.0 mm from areas selected by each study’s pathologist. 34.1% of cases had 1 core, 58.4% 2 cores, 7.1% 3 cores, and 0.4% 4 cores. See Supplementary Materials for additional details.

### Immune marker staining and scoring

All staining and scoring were performed at BC Cancer, Victoria. MOCOG TMAs were stained by multi-color IHC or IF with four panels of antibodies:(64) panel A detected CD3 and CD8; panel B detected CD20 and CD79; panel C detected CD8, FoxP3 and CD25; and panel D detected PD-1, PD-L1 and CD68. All panels detected pan-cytokeratin to identify tumor epithelium. CD4+ T cells were defined as CD3+CD8-cells.(36) See Supplementary Materials for additional details.

### Statistics

Immune-marker density (D; cells/mm^2^) for a particular marker was calculated separately for epithelial and stromal compartments. For cases with multiple cores, the epithelial area was taken as the sum of all their individual TMA epithelial areas and similarly for the stromal area. For analyses including all participants, we transformed the densities (D) by raising them to the power 0.25 (D^0.25^); this transformation gave close to the maximum log-likelihood of the fitted models across the range of immune subsets and substantially reduced the skewness of the distribution of the values.(65) To provide categorical comparisons and to better appreciate the magnitude of the associations, we also categorized marker D values into quartiles separately for the five largest studies (AUS, DOV, MAY, SEA and VAN), based on the distribution of the D values of the STS participants, separately for epithelial and stromal markers. If the proportion of zero D values was greater than 50% overall, we compressed the quartile values into two categories (zero, non-zero). Quartile analyses was not appropriate for the remaining studies due to smaller sample sizes. Statistical significance was defined as p≤0.05 using a two-sided test. No adjustments were made for multiple testing. Analyses were conducted using R Studio (version 1.3.1073) and Stata (version 16). See Supplementary Materials for additional details.

## Supplementary Material

Supplementary Material

## Figures and Tables

**Table 1:** Characteristics of the study population by survival group.

	LTS^A^ 10+ Years (n=374)	MTS^A^ 5-7.99 Years (n=433)	STS^A^ 2-4.99 Years (n=416)	Total (n=1223)
**Continent**				
Australia	119 (31.8%)	126 (29.1%)	126 (30.3%)	371 (30.3%)
Europe	47 (12.6%)	48 (11.1%)	52 (12.5%)	148 (12.1%)
North America	202 (54.0%)	255 (58.9%)	232 (55.8%)	689 (56.3%)
South America	6 (1.6%)	4 (0.9%)	6 (1.4%)	16 (1.3%)
**Age**				
Median [Q1^B^, Q3^B^]	59 [51, 67]	60 [53, 66]	60 [52, 66]	60.0 [52, 66]
**Race/Ethnicty**				
White	259 (92.2%)	308 (94.8%)	285 (91.9%)	852 (93.0%)
Asian	13 (4.6%)	8 (2.5%)	11 (3.6%)	32 (3.5%)
Black	0 (0%)	2 (0.6%)	0 (0%)	2 (0.2%)
Other	9 (3.2%)	7 (2.2%)	14 (4.5%)	30 (3.3%)
Not Available	135 (32.5%)	108 (24.9%)	64 (17.1%)	307 (25.1%)
**Year of diagnosis**				
Range	1989-2010	1987-2011	1985-2011	1985-2011
**FIGO stage**				
III (NOS)	29 (7.8%)	30 (6.9%)	33 (7.9%)	92 (7.5%)
IIIA	20 (5.3%)	17 (3.9%)	6 (1.4%)	43 (3.5%)
IIIB	42 (11.2%)	32 (7.4%)	28 (6.7%)	102 (8.3%)
IIIC	201 (53.7%)	272 (62.8%)	244 (58.7%)	717 (58.6%)
IV	49 (13.1%)	48 (11.1%)	73 (17.5%)	170 (13.9%)
III/IV^C^	33 (8.8%)	34 (7.9%)	32 (7.7%)	99 (8.1%)

A LTS, long-term survivors; MTS, medium-term survivors; STS, short-term survivors.

B Q1, cutpoint between first and second quartiles; Q3, cutpoint between third and fourth quartiles.

C Stages III and IV not separated.

**Table 2:** Distribution of the immune-cell densities, overall and by epithelial content.

Marker	Area	Cell type	Overall (n=1,223)				Epithelium-low (n=618)				Epithelium-high (n=605)		
			Median	Q1^A^	Q3^A^		Median	Q1	Q3		Median	Q1	Q3
CD8+FoxP3+-	Epithelial	CD8+ T cell	60.5	15.9	175		71.3	19.4	213		52.5	15.0	151
CD8+FoxP3+-	Stromal	CD8+ T cell	212	66.1	536		237	77.9	593		191	56.5	468
CD8+FoxP3-	Epithelial	CD8+FoxP3- T cell	58.6	15.7	173		71.3	19.4	213		51.5	14.7	147
CD8+FoxP3-	Stromal	CD8+FoxP3- T cell	207	64.2	519		235	77.1	568		185	56.5	442
CD8+FoxP3+	Epithelial	CD8+FoxP3+ T cell	0	0	2.0		0	0.0	2.2		0	0	1.9
CD8+FoxP3+	Stromal	CD8+FoxP3+ T cell	0	0	11.3		2.0	0.0	13.2		0	0	8.3
CD3+CD8-	Epithelial	CD4+ T cell	11.6	0	36.8		12.4	0.0	44.4		11.0	2.2	31.4
CD3+CD8-	Stromal	CD4+ T cell	39.7	7.5	114		42.4	9.2	129.3		37.8	5.0	104
CD8-FoxP3+	Epithelial	Presumptive Treg cell	16.0	4.3	39.4		16.7	5.0	41.4		15.0	3.5	37.4
CD8-FoxP3+	Stromal	Presumptive Treg cell	74.0	18.3	194		82.5	25.9	200		66.0	10.1	180
CD20+CD79+	Epithelial	B cell	0	0	3.8		0	0	5.5		0	0	2.6
CD20+CD79+	Stromal	B cell	0	0	21.9		4.9	0	26.2		0	0	17.6
CD20-CD79+	Epithelial	Plasma cell	0	0	4.0		0	0	4.7		0	0	3.0
CD20-CD79+	Stromal	Plasma cell	13.1	0	103		24.8	0	122		0	0	89.3
PD-1 +	Epithelial	PD-1+ immune cell	31.1	7.3	111		33.6	8.6	123		28.3	6.3	104
PD-1 +	Stromal	PD-1+ immune cell	70.2	15.6	244		84.8	22.5	273		62.8	8.0	223
CD68+PD-L1 +	Epithelial	CD68+PD-L1+ TAM cell	1.8	0	35.5		0	0	40.5		1.9	0	29.6
CD68+PD-L1 +	Stromal	CD68+PD-L1+ TAM cell	4.5	0	105		5.0	0	111		0	0	92.5
CD68+PD-L1-	Epithelial	CD68+PD-L1- TAM cell	163	77.7	309		149	70.9	298		172	86.3	320
CD68+PD-L1-	Stromal	CD68+PD-L1- TAM cell	291	127	605		280	119	550		302	134	681
CD68-PD-L1 +	Epithelial	CD68-PD-L1+ cell	0	0	26.4		0	0	43.3		0	0	19.0
CD68-PD-L1 +	Stromal	CD68-PD-L1+ cell	2.3	0	61.3		4.5	0	78.1		0	0	53.2

A Q1, cutpoint between first and second quartiles; Q3, cutpoint between third and fourth quartiles.

**Table 3:** D^0.25^ odds ratios (ORs) of immune-cell subsets comparing survival groups.^A^

Marker	Area	Cell type	LTS compared to STS (n=790)				MTS compared to STS (n=849)				LTS compared to MTS (n=807)		
			OR	95% CI	p		OR	95% CI	p		OR	95% CI	p
CD8+FoxP3+-	Epithelial	CD8+ T cell	1.24	1.10 - 1.40	**<0.001**		1.10	0.99 - 1.22	0.080		1.10	0.99 - 1.22	0.092
CD8+FoxP3+-	Stromal	CD8+ T cell	1.12	1.02 - 1.23	**0.022**		1.01	0.92 - 1.11	0.86		1.10	1.00 - 1.21	**0.050**
CD8+FoxP3-	Epithelial	CD8+FoxP3- T cell	1.24	1.10 - 1.40	**<0.001**		1.10	0.99 - 1.22	0.084		1.10	0.99 - 1.22	0.092
CD8+FoxP3-	Stromal	CD8+FoxP3- T cell	1.12	1.01 - 1.23	**0.024**		1.01	0.92 - 1.10	0.90		1.10	1.00 - 1.21	0.049
CD8+FoxP3+	Epithelial	CD8+FoxP3+ T cell	1.40	1.14 - 1.72	**0.001**		1.19	0.97 - 1.45	0.091		1.17	0.97 - 1.41	0.11
CD8+FoxP3+	Stromal	CD8+FoxP3+ T cell	1.25	1.08 - 1.43	**0.002**		1.11	0.97 - 1.26	0.14		1.11	0.97 - 1.26	0.13
CD3+CD8-	Epithelial	CD4+ T cell	1.21	1.05 - 1.39	**0.007**		1.19	1.04 - 1.36	**0.010**		1.02	0.89 - 1.17	0.80
CD3+CD8-	Stromal	CD4+ T cell	1.13	1.01 - 1.25	**0.028**		1.08	0.97 - 1.20	0.16		1.05	0.94 - 1.16	0.40
CD8-FoxP3+	Epithelial	Presumptive Treg cell	1.11	0.96 - 1.29	0.15		1.12	0.97 - 1.29	0.12		1.00	0.87 - 1.16	0.95
CD8-FoxP3+	Stromal	Presumptive Treg cell	1.17	1.05 - 1.30	**0.004**		1.07	0.97 - 1.19	0.19		1.08	0.97 - 1.21	0.14
CD20+CD79+	Epithelial	B cell	1.27	1.09 - 1.48	**0.002**		1.22	1.05 - 1.42	**0.010**		1.05	0.92 - 1.20	0.49
CD20+CD79+	Stromal	B cell	1.07	0.97 - 1.18	0.20		1.04	0.94 - 1.15	0.45		1.04	0.94 - 1.15	0.49
CD20-CD79+	Epithelial	Plasma cell	1.22	1.05 - 1.41	**0.008**		1.17	1.01 - 1.35	**0.032**		1.05	0.92 - 1.19	0.51
CD20-CD79+	Stromal	Plasma cell	1.15	1.06 - 1.24	**0.001**		1.06	0.98 - 1.15	0.13		1.08	1.00 - 1.17	**0.047**
PD-1 +	Epithelial	PD-1+ immune cell	1.33	1.17 - 1.51	**<0.001**		1.19	1.05 - 1.34	**0.007**		1.15	1.01 - 1.30	**0.032**
PD-1 +	Stromal	PD-1+ immune cell	1.18	1.07 - 1.31	**0.001**		1.06	0.96 - 1.17	**0.27**		1.12	1.01 - 1.24	**0.031**
CD68+PD-L1 +	Epithelial	CD68+PD-L1+ TAM cell	1.15	1.00 - 1.31	**0.043**		1.12	0.98 - 1.27	**0.098**		1.01	0.89 - 1.14	**0.92**
CD68+PD-L1 +	Stromal	CD68+PD-L1+ TAM cell	1.10	1.00 - 1.22	**0.053**		1.04	0.94 - 1.14	**0.49**		1.04	0.95 - 1.15	**0.38**
CD68+PD-L1-	Epithelial	CD68+PD-L1- TAM cell	0.93	0.78 - 1.11	**0.44**		1.06	0.90 - 1.25	**0.49**		0.86	0.72 - 1.02	**0.087**
CD68+PD-L1-	Stromal	CD68+PD-L1- TAM cell	0.94	0.82 - 1.07	**0.36**		0.97	0.86 - 1.10	**0.63**		0.97	0.85 - 1.10	**0.60**
CD68-PD-L1 +	Epithelial	CD68-PD-L1+ cell	1.22	1.07 - 1.38	**0.002**		1.09	0.97 - 1.23	**0.14**		1.08	0.96 - 1.21	**0.21**
CD68-PD-L1 +	Stromal	CD68-PD-L1+ cell	1.18	1.07 - 1.31	**0.001**		1.06	0.96 - 1.17	**0.25**		1.11	1.00 - 1.22	**0.048**

A LTS, long-term survivors; MTS, medium-term survivors; STS, short-term survivors.

**Table 4:** D^0.25^ odds ratios (ORs) of immune-cell subsets comparing long-term survivors (LTS) to short-term survivors (STS) by epithelium group.

Marker	Area	Cell type		Epithelium-low (n=392)			Epithelium-high (n=398)		
			OR	95% CI	p		OR	95% CI	p
CD8+FoxP3+-	Epithelial	CD8+ T cell	1.17	1.00 - 1.37	**0.048**		1.33	1.10 - 1.61	**0.003**
CD8+FoxP3+-	Stromal	CD8+ T cell	1.04	0.90 - 1.20	0.62		1.17	1.02 - 1.33	**0.022**
CD8+FoxP3-	Epithelial	CD8+FoxP3- T cell	1.17	1.00 - 1.37	**0.048**		1.33	1.10 - 1.61	**0.003**
CD8+FoxP3-	Stromal	CD8+FoxP3- T cell	1.04	0.90 - 1.20	0.63		1.17	1.02 - 1.33	**0.025**
CD8+FoxP3+	Epithelial	CD8+FoxP3+ T cell	1.25	0.94 - 1.67	0.13		1.49	1.10 - 2.01	**0.010**
CD8+FoxP3+	Stromal	CD8+FoxP3+ T cell	1.11	0.90 - 1.36	0.32		1.36	1.11 - 1.67	**0.003**
CD3+CD8-	Epithelial	CD4+ T cell	1.11	0.92 - 1.33	0.28		1.42	1.14 - 1.76	**0.001**
CD3+CD8-	Stromal	CD4+ T cell	1.02	0.87 - 1.19	0.85		1.25	1.07 - 1.45	**0.004**
CD8-FoxP3+	Epithelial	Presumptive Treg cell	0.99	0.81 - 1.22	0.96		1.26	1.02 - 1.57	**0.034**
CD8-FoxP3+	Stromal	Presumptive Treg cell	1.07	0.89 - 1.29	0.45		1.21	1.06 - 1.39	**0.006**
CD20+CD79+	Epithelial	B cell	1.14	0.93 - 1.39	0.20		1.49	1.16 - 1.90	**0.002**
CD20+CD79+	Stromal	B cell	0.92	0.80 - 1.07	0.29		1.26	1.07 - 1.48	**0.004**
CD20-CD79+	Epithelial	Plasma cell	1.11	0.90 - 1.36	0.33		1.35	1.08 - 1.68	**0.009**
CD20-CD79+	Stromal	Plasma cell	1.09	0.96 - 1.23	0.17		1.19	1.06 - 1.33	**0.003**
PD-1 +	Epithelial	PD-1+ immune cell	1.31	1.10 - 1.56	**0.003**		1.37	1.14 - 1.65	**0.001**
PD-1 +	Stromal	PD-1+ immune cell	1.17	0.99 - 1.37	0.061		1.19	1.04 - 1.36	**0.012**
CD68+PD-L1 +	Epithelial	CD68+PD-L1+ TAM cell	1.13	0.93 - 1.38	0.21		1.16	0.96 - 1.40	0.13
CD68+PD-L1 +	Stromal	CD68+PD-L1+ TAM cell	1.02	0.87 - 1.19	0.80		1.16	1.01 -1.33	**0.030**
CD68+PD-L1-	Epithelial	CD68+PD-L1- TAM cell	0.83	0.64 - 1.08	0.16		1.15	0.88 -1.49	0.31
CD68+PD-L1-	Stromal	CD68+PD-L1- TAM cell	0.87	0.70 - 1.07	0.19		1.01	0.85 - 1.49	0.90
CD68-PD-L1 +	Epithelial	CD68-PD-L1+ cell	1.14	0.95 - 1.37	0.15		1.24	1.03 - 1.50	**0.022**
CD68-PD-L1 +	Stromal	CD68-PD-L1+ cell	1.13	0.98 - 1.31	0.10		1.21	1.05 - 1.40	**0.009**

**Table 5:** Odds ratios for the joint effects of intra-epithelial CD8+ T cells (per quartile) and intra-stromal B cells (zero/non-zero) for being a long-term survivor (LTS) compared to a short-term survivor (STS).^A^

Intra-epithelial CD8+ T cell^B^		Intra-stromal B cell	
		Zero	Non-zero
	Quartile 1	1.00	1.74
	Quartile 2	1.41	2.45
	Quartile 3	1.99	3.46
	Quartile 4	2.80	4.87

A p<0.001 for overall model fit.

B Quartiles fit as a linear term.

**Table 6:** Distribution of the immune-cell densities by molecular subtype (PrOTYPE).

Marker	Area	Cell type	C1/Mesenchymal (n=165)				C2/Immunoreactive (n=187)				C4/Differentiated (n=232)				C5/Proliferative (n=110)		
			Median	Q1^A^	Q3^A^		Median	Q1	Q3		Median	Q1	Q3		Median	Q1	Q3
CD8+FoxP3+-	Epithelial	CD8+ T cell	44.6	15.2	125		146	54.5	322		54.4	16.8	148		12.7	2.0	45.5
CD8+FoxP3+-	Stromal	CD8+ T cell	221	78.3	484		425	142	823		162	63.5	365		52.2	5.6	216
CD8+FoxP3-	Epithelial	CD8+FoxP3- T cell	44.6	15.2	125		144	52.7	318		53.2	16.8	146		12.7	2.0	44.9
CD8+FoxP3-	Stromal	CD8+FoxP3- T cell	218	78.3	477		415	141	806		160	58.3	353		52.2	5.6	210
CD8+FoxP3+	Epithelial	CD8+FoxP3+ T cell	0.0	0.0	2.1		0.0	0.0	3.5		0.0	0.0	1.6		0.0	0.0	0.0
CD8+FoxP3+	Stromal	CD8+FoxP3+ T cell	0.0	0.0	11.5		5.4	0.0	21.4		0.0	0.0	6.6		0.0	0.0	0.0
CD3+CD8-	Epithelial	CD4+ T cell	10.5	0.0	36.4		20.4	7.6	55.9		10.8	0.0	36.1		2.0	0.0	10.2
CD3+CD8-	Stromal	CD4+ T cell	48.7	6.8	120		72.7	20.6	186		35.4	0.0	90.0		9.2	0.0	40.8
CD8-FoxP3+	Epithelial	Presumptive Treg cell	15.8	5.5	37.1		24.7	12.1	56.7		12.8	4.2	32.1		2.3	0.0	9.5
CD8-FoxP3+	Stromal	Presumptive Treg cell	97.9	34.3	206		144	61.4	253		49.6	11.1	158		12.9	0.0	53.9
CD20+CD79+	Epithelial	B cell	0.0	0.0	3.1		0.0	0.0	7.8		0.0	0.0	2.0		0.0	0.0	0.0
CD20+CD79+	Stromal	B cell	4.4	0.0	25.8		7.7	0.0	42.3		0.0	0.0	11.2		0.0	0.0	2.5
CD20-CD79+	Epithelial	Plasma cell	0.0	0.0	0.0		0.0	0.0	8.1		0.0	0.0	1.8		0.0	0.0	0.0
CD20-CD79+	Stromal	Plasma cell	19.3	0.0	88.6		37.9	0.0	245		2.5	0.0	77.6		0.0	0.0	23.6
PD-1 +	Epithelial	PD-1+ immune cell	25.2	5.3	103		68.2	14.6	145		21.2	6.2	78.3		7.7	0.0	29.7
PD-1 +	Stromal	PD-1+ immune cell	74.2	24.7	253		124	31.0	394		48.1	9.0	137		19.2	0.0	67.3
CD68+PD-L1 +	Epithelial	CD68+PD-L1+ TAM cell	1.7	0.0	22.5		6.1	0.0	47.3		0.0	0.0	16.0		0.0	0.0	2.2
CD68+PD-L1 +	Stromal	CD68+PD-L1+ TAM cell	0.0	0.0	65.4		17.6	0.0	164		0.0	0.0	47.1		0.0	0.0	0.0
CD68+PD-L1-	Epithelial	CD68+PD-L1- TAM cell	187	102	338		200	107	329		185	89.3	352		84.4	48.5	153
CD68+PD-L1-	Stromal	CD68+PD-L1- TAM cell	359	151	745		364	178	624		303	142	666		155	86.9	285
CD68-PD-L1 +	Epithelial	CD68-PD-L1+ cell	0.0	0.0	15.7		5.9	0.0	39.0		0.0	0.0	10.6		0.0	0.0	2.3
CD68-PD-L1 +	Stromal	CD68-PD-L1+ cell	2.2	0.0	26.5		11.9	0.0	101.1		0.0	0.0	24.8		0.0	0.0	6.1

A Q1, cutpoint between first and second quartiles; Q3, cutpoint between third and fourth quartiles.

**Table 7:** D^0.25^ odds ratios (ORs) of immune-cell subsets comparing long-term survivors (LTS) to short-term survivors (STS) by PrOTYPE.

Marker	Area	Cell type	C1/Mesenchymal (n=109)				C2/Immunoreactive (n=108)				C4/Differentiated (n=155)				C5/Proliferative (n=71)		
			OR	95% CI	p		OR	95% CI	p		OR	95% CI	p		OR	95% CI	p
CD8+FoxP3+-	Epithelial	CD8+ T cell	1.30	0.91 - 1.86	0.15		1.31	0.89 - 1.92	0.17		1.39	0.99 - 1.94	0.059		1.09	0.70 - 1.70	0.69
CD8+FoxP3+-	Stromal	CD8+ T cell	1.24	0.90 - 1.73	0.19		1.19	0.85 - 1.66	0.31		1.22	0.93 - 1.59	0.15		1.15	0.85 - 1.57	0.36
CD8+FoxP3-	Epithelial	CD8+FoxP3- T cell	1.30	0.91 - 1.86	0.16		1.30	0.88 - 1.91	0.18		1.38	0.98 - 1.94	0.063		1.10	0.70 - 1.71	0.68
CD8+FoxP3-	Stromal	CD8+FoxP3- T cell	1.25	0.90 - 1.74	0.19		1.18	0.84 - 1.66	0.33		1.22	0.93 - 1.59	0.15		1.15	0.85 - 1.57	0.37
CD8+FoxP3+	Epithelial	CD8+FoxP3+ T cell	1.28	0.73 - 2.25	0.39		1.89	1.03 - 3.47	**0.041**		2.00	1.14 - 3.51	**0.016**		0.85	0.25 - 2.84	0.79
CD8+FoxP3+	Stromal	CD8+FoxP3+ T cell	1.26	0.85 - 1.87	0.26		1.14	0.79 - 1.63	0.49		1.43	0.99 - 2.08	0.058		2.13	0.74 - 6.10	0.16
CD3+CD8-	Epithelial	CD4+ T cell	1.08	0.71 - 1.63	0.73		1.30	0.83 - 2.02	0.25		1.19	0.85 - 1.67	0.32		1.37	0.71 - 2.63	0.35
CD3+CD8-	Stromal	CD4+ T cell	1.07	0.77 - 1.47	0.70		1.17	0.82 - 1.66	0.38		1.19	0.92 - 1.55	0.19		1.05	0.69 - 1.61	0.81
CD8-FoxP3+	Epithelial	Presumptive Treg cell	1.07	0.70 - 1.64	0.76		1.50	0.81 - 2.76	0.20		1.43	0.98 - 2.08	0.065		0.98	0.59 - 1.61	0.93
CD8-FoxP3+	Stromal	Presumptive Treg cell	1.12	0.78 - 1.59	0.55		0.99	0.58 - 1.71	0.98		1.40	1.09 - 1.79	**0.008**		1.18	0.80 - 1.74	0.41
CD20+CD79+	Epithelial	B cell	0.90	0.56 - 1.42	0.64		1.12	0.79 - 1.60	0.52		1.64	1.09 - 2.49	**0.019**		0.82	0.36 - 1.91	0.65
CD20+CD79+	Stromal	B cell	0.92	0.68 - 1.26	0.62		0.96	0.76 - 1.21	0.73		1.09	0.84 - 1.42	0.53		0.70	0.40 - 1.22	0.21
CD20-CD79+	Epithelial	Plasma cell	1.31	0.79 - 2.16	0.30		1.07	0.75 - 1.54	0.70		1.53	1.05 - 2.24	**0.028**		1.22	0.52 - 2.87	0.65
CD20-CD79+	Stromal	Plasma cell	0.96	0.73 - 1.27	0.78		1.00	0.83 - 1.21	0.96		1.31	1.07 - 1.60	**0.010**		0.99	0.67 - 1.45	0.95
PD-1 +	Epithelial	PD-1+ immune cell	1.44	0.96 - 2.17	0.08		1.18	0.84 - 1.66	0.35		1.57	1.11- 2.22	**0.011**		1.08	0.65 - 1.80	0.77
PD-1 +	Stromal	PD-1+ immune cell	1.20	0.86 - 1.69	0.28		0.96	0.74 - 1.25	0.76		1.53	1.14 - 2.06	**0.005**		1.16	0.78 - 1.72	0.46
CD68+PD-L1 +	Epithelial	CD68+PD-L1+ TAM cell	1.22	0.82 - 1.82	0.33		0.92	0.65 - 1.29	0.62		1.31	0.95 - 1.80	**0.097**		1.39	0.59 - 3.26	0.45
CD68+PD-L1 +	Stromal	CD68+PD-L1+ TAM cell	1.11	0.84 - 1.47	0.48		1.01	0.77 - 1.31	0.97		1.19	0.95 - 1.48	**0.13**		0.74	0.38 - 1.43	0.37
CD68+PD-L1-	Epithelial	CD68+PD-L1- TAM cell	1.14	0.63 - 2.04	0.67		0.87	0.49 - 1.54	0.63		1.02	0.69 - 1.52	**0.91**		0.82	0.36 - 1.89	0.65
CD68+PD-L1-	Stromal	CD68+PD-L1- TAM cell	1.15	0.75 - 1.78	0.53		0.82	0.54 - 1.24	0.34		0.88	0.66 - 1.19	**0.41**		0.93	0.47 - 1.83	0.83
CD68-PD-L1 +	Epithelial	CD68-PD-L1+ cell	1.13	0.76 - 1.70	0.55		0.91	0.64 - 1.28	0.58		1.61	1.16 - 2.24	**0.005**		1.10	0.53 - 2.28	0.79
CD68-PD-L1 +	Stromal	CD68-PD-L1+ cell	1.26	0.91 - 1.75	0.16		0.95	0.73 - 1.25	0.74		1.42	1.10 - 1.84	**0.007**		1.09	0.60 - 1.99	0.78

**Table 8:** D^0.25^ odds ratios (ORs) of immune-cell subsets comparing epithelium-high long-term suvivors (LTS) to short-term survivors (STS) by PrOTYPE.

Marker	Area	Cell type	Epithelium-high														
			C1/Mesenchymal (n=31)				C2/Immunoreactive (n=61)				C4/Differentiated (n=104)				C5/Proliferative (n=33)		
			OR	95% CI	p		OR	95% CI	p		OR	95% CI	p		OR	95% CI	p
CD8+FoxP3+-	Epithelial	CD8+ T cell	1.20	0.58 - 2.51	0.62		1.01	0.55 - 1.84	0.98		2.03	1.26 - 3.29	**0.004**		1.38	0.68 - 2.78	0.37
CD8+FoxP3+-	Stromal	CD8+ T cell	0.99	0.60 - 1.65	0.98		1.01	0.64 - 1.59	0.98		1.43	1.03 - 1.99	**0.035**		1.05	0.71 - 1.55	0.80
CD8+FoxP3-	Epithelial	CD8+FoxP3- T cell	1.20	0.58 - 2.50	0.63		1.00	0.54 - 1.83	0.99		2.04	1.26 - 3.32	**0.004**		1.37	0.68 - 2.78	0.38
CD8+FoxP3-	Stromal	CD8+FoxP3- T cell	0.99	0.60 - 1.65	0.97		1.00	0.63 - 1.59	0.99		1.44	1.03 - 2.01	**0.034**		1.05	0.71 - 1.55	0.81
CD8+FoxP3+	Epithelial	CD8+FoxP3+ T cell	1.78	0.45 - 7.10	0.42		1.33	0.61 - 2.92	0.47		1.77	0.92 - 3.43	0.089		n/a^A^	. - .	.
CD8+FoxP3+	Stromal	CD8+FoxP3+ T cell	1.27	0.53 - 3.06	0.60		1.15	0.70 - 1.92	0.58		1.39	0.91 - 2.12	0.13		n/a^A^	. - .	.
CD3+CD8-	Epithelial	CD4+ T cell	1.09	0.48 - 2.48	0.84		2.04	0.96 - 4.31	0.062		1.36	0.91 - 2.04	0.13		n/a^A^	. - .	.
CD3+CD8-	Stromal	CD4+ T cell	0.92	0.53 - 1.60	0.78		1.45	0.84 - 2.50	0.18		1.30	0.96 - 1.76	0.093		1.89	0.84 - 4.27	0.13
CD8-FoxP3+	Epithelial CD8-FoxP3+ Treg cell		0.76	0.28 - 2.03	0.58		1.14	0.42 - 3.11	0.80		1.77	1.08 - 2.91	**0.023**		1.59	0.59 - 4.27	0.35
CD8-FoxP3+	Stromal	CD8-FoxP3+ Treg cell	0.67	0.34 - 1.35	0.26		0.83	0.39 - 1.74	0.62		1.44	1.08 - 1.93	**0.015**		1.20	0.67 - 2.15	0.54
CD20+CD79+	Epithelial	CD20+ B cell	0.54	0.12 - 2.47	0.43		1.14	0.62 - 2.13	0.67		1.75	1.07 - 2.87	**0.026**		1.28	0.18 - 8.97	0.81
CD20+CD79+	Stromal	CD20+ B cell	1.07	0.55 - 2.10	0.84		1.02	0.72 - 1.44	0.91		1.30	0.93 - 1.84	0.13		1.22	0.33 - 4.55	0.77
CD20-CD79+	Epithelial	CD20-CD79+ Plasma cell	n/a^A^	. - .	.		0.89	0.57 - 1.40	0.62		1.65	1.06 - 2.58	**0.027**		2.40	0.41 - 14.08	0.33
CD20-CD79+	Stromal	CD20-CD79+ Plasma cell	0.91	0.51 - 1.59	0.73		0.87	0.67 - 1.13	0.30		1.38	1.09 - 1.76	**0.009**		1.53	0.57 - 4.10	0.40
PD-1 +	Epithelial	PD-1+ Immune cell	0.98	0.55 - 1.76	0.96		0.72	0.43 - 1.19	0.20		2.19	1.33 - 3.60	**0.002**		1.07	0.42 - 2.76	0.89
PD-1 +	Stromal	PD-1+ Immune cell	0.82	0.46 - 1.45	0.49		0.67	0.44 - 1.03	0.068		1.94	1.31 - 2.85	**0.001**		1.38	0.75 - 2.52	0.30
CD68+PD-L1 +	Epithelial	CD68+PD-L1+ TAM cell	1.14	0.57 - 2.28	0.71		0.62	0.37 - 1.06	0.083		1.47	1.00 - 2.16	**0.049**		1.90	0.36 - 10.17	0.45
CD68+PD-L1 +	Stromal	CD68+PD-L1+ TAM cell	1.17	0.74 - 1.84	0.50		0.92	0.67 - 1.27	0.62		1.28	0.99 - 1.66	**0.062**		0.70	0.12 - 3.96	0.69
CD68+PD-L1-	Epithelial	CD68+PD-L1- TAM cell	1.03	0.41 - 2.59	0.94		0.89	0.42 - 1.90	0.77		1.27	0.74 - 2.15	**0.38**		1.09	0.30 - 4.03	0.89
CD68+PD-L1-	Stromal	CD68+PD-L1- TAM cell	1.08	0.51 - 2.27	0.85		0.90	0.55 - 1.46	0.66		0.88	0.61 - 1.27	**0.49**		0.64	0.20 - 2.10	0.46
CD68-PD-L1 +	Epithelial	CD68-PD-L1+ cell	1.09	0.55 - 2.17	0.80		0.83	0.52 - 1.32	0.43		1.76	1.18 - 2.63	**0.006**		1.56	0.38 - 6.49	0.54
CD68-PD-L1 +	Stromal	CD68-PD-L1+ cell	1.17	0.73 - 1.86	0.52		0.92	0.65 - 1.29	0.62		1.63	1.19 - 2.23	**0.003**		n/a^A^	. - .	.

A n/a=could not be calculated.

## Data Availability

Individual patient data and related tumor information underlying this article cannot be shared publicly due to data privacy protection laws. Requests for further analyses to be done on a collaborative basis will be addressed on reasonable request to the corresponding author. No custom code or software was used.
