# Supplementary material for: Immunological and molecular features of the tumor microenvironment of long-term survivors of ovarian cancer
Source: J Clin Invest. Author manuscript; Available in PMC 2024 Dec 14. (PMC11645148; doi:10.1172/JCI179501)
Supplement: Supplementary Material [file EMS201285-supplement-Supplementary_Material.pdf]

SUPPLEMENTARY MATERIALS

SUPPLEMENTARY METHODS

Study population details

The MOCOG cohort was assembled from 19 studies, including two studies from Australia, six from Europe, ten from North America, and one from Brazil (total n=1,298 tumors of which 1,223 were successfully stained and scored; **Supplementary Table 1**). The two Australian studies were combined, as the protocols were similar. All patients had been diagnosed with FIGO Stage III/IV ovarian, fallopian tube or primary peritoneal HGSC. LTS was defined by the funding agency as a survivor of 10+ years from the date of diagnosis. STS and MTS patients were defined as a survivor of 2-4.99 years and 5-7.99 years from the date of diagnosis, respectively. Patients were diagnosed between 1985 and 2011. The comparison groups of STS and MTS were frequency matched to LTS patients by study (or, in rare circumstances affecting some European and North American studies, to a study from the same geographical region if no suitable within-study matches were found); by year of diagnosis, to account for global changes in ovarian cancer treatment trends (grouped as ≤1994, 1995-1999, 2000-2004, ≥2005); and by patient age at diagnosis (grouped as ≤39, 40-49, 50-59, 60-69, ≥70 years). The five largest studies collectively contributed 78% of the samples (n=956): AUS (AOC+WMH, Australia; n=371); DOV (Washington, USA; n=205); MAY (Minnesota, USA; n=172); SEA (Cambridge, United Kingdom; n=87); and VAN (British Columbia, Canada; n=121).

For the majority of cases, a specialist gynecological cancer pathologist reviewed haematoxylin and eosin-stained section(s) from each case to confirm tumor histology consistent with HGSC.(1, 2) We further restricted cases to those with molecular features consistent with HGSC; this resulted in removing 22 cases that lacked evidence of a *TP53* mutation following next-generation panel sequencing and/or immunohistochemistry showing a normal (wild-type) p53 staining pattern (3) with a concomitant Ras-pathway gene mutation (*BRAF, KRAS or NRAS*), indicative of low-grade serous carcinoma.(4)

Of the 1,223 cases that were successfully stained, the majority of samples were from adnexal (including pelvic region, lower pelvis) tumors (n=649); the remainder were from omentum (n=152) or other anatomical sites (n=33); 389 sites were not known. We conducted sensitivity analyses restricting attention to adnexal samples. For 747 of the cases, information was available on whether they received primary cytoreductive surgery (PCS) or neoadjuvant chemotherapy (NACT); 689/747 (92.2%) of these patients underwent PCS, and 58/747 (7.8%) received NACT.-Among LTS, 5.2% received NACT compared to 7.8% and 10.0% of MTS and STS, respectively. This low percentage is consistent with clinical practice during the era these cases were accrued. As all participants included in this MOCOG study were from the same era, it is unlikely that the NACT rate significantly exceeded 8% among patients for whom those data were unavailable. We therefore included all participants in the analyses and conducted sensitivity analyses restricted to those who were known to have had PCS.

Additional datasets included the Canadian Ovarian Experimental Unified Resource (COEUR; n=981) (5) and the Ovarian Outcomes Unit (OOU; n=192); the latter is comprised of cases who had undergone optimal cytoreduction (i.e., without evidence of macroscopic residual disease) and had previously been studied for numerous immune cell markers.(6-11) Because we did not have access to COEUR patient identifiers, it is possible that up to 10% of our VAN patients overlapped with COEUR. There may also have been overlap between MOCOG and OOU cases; however, the latter were used for only one part of the study involving specific phenotypic subsets of T cells that were not evaluated in MOCOG samples.

Immune marker staining and scoring details

All reagents used for mcIHC/mcIF were from Biocare Medical (Pacheco, CA) unless otherwise stated, and all staining was performed at room temperature. For mcIHC with panels A and B, slides were deparaffinized through xylene and graded alcohols then subjected to antigen retrieval in a decloaking chamber with Diva decloaking solution. Slides were then loaded on the Intellipath FLX autostainer. Following blocking with peroxidased-1 and background sniper, a

cocktail of either CD3 (clone SP7, Spring Bioscience) and CD8 (clone C8/144b, Cell Marque) or CD20 (Clone L26, Biocare) and CD79a (clone SP18, Abcam) was added to the slide for 30 minutes followed by MACH 2 Double Stain #2 polymer for 30 minutes. Warp Red chromogen was applied for 7 minutes, then DAB chromogen for 5 minutes. The slides were then removed from the stainer and subjected to denaturation with a pH2.0 SDS-Glycine solution for 45 minutes at 50^0C as per Pirici et al.(12) For the second round of staining, slides were incubated with pan-cytokeratin antibody (clone AE1/AE3+5D3, Biocare) for 30 minutes; then Mach 2 Mouse-AP polymer, Ferangi Blue chromogen for 8 minutes; and finally, a 1/5 dilution of CAT Hematoxylin for 5 minutes. Slides were washed, airdried and cover-slipped with Ecomount.

mcIF panels C and D used OPAL staining reagents (Akoya Biosciences) as indicated in addition to the above-mentioned Biocare reagents. Protocols for slide preparation were similar to the mcIHC panels with the exception that a post-fixation step of 20 minutes in 10% neutral buffered formalin (Sigma) was performed after deparaffinization. Four sequential rounds of staining were performed with a microwave denaturation step using AR6 (Akoya) between each round. Each round of staining used a single primary antibody followed by MACH 4-HRP (for CD25) or MACH 2-HRP polymers and an OPAL fluor. The antibody-fluor pairings in order for panel C were: anti-CD25 (clone 4C9, Lab Vision) + OPAL520; anti-CD8 (clone C8/144B, Cell Marque) + OPAL570; anti-FoxP3 (clone 236A/E7, Abcam) + OPAL690; and anti-pan-cytokeratin (clone AE1/AE3+5D3, Biocare) + Coumarin. The pairings for panel D were: anti-PD-1 (clone EPR4877(2), Abcam) + OPAL650; anti-CD68 (Clone SP251, Spring Bioscience) + OPAL520; anti-pan-cytokeratin (clone AE1/AE3+5D3, Biocare) + OPAL690; and anti-PD-L1 (Clone SP142, Spring Bioscience) + OPAL570.

For the COUER cohort, the 6-color mcIF panels followed the same steps as the above 4-color panels. The antibody-fluor pairings in order for the B and T cell panel were: anti-CD79a (clone SP18) + OPAL520; anti-CD20 (clone L26) + OPAL620; anti-CD8 (clone C8/144B) + OPAL690; anti-CD3 (Clone PS1, Biocare) + OPAL480; anti-FoxP3 (clone 236A/E7) + OPAL

570; and anti-pan-cytokeratin (clone AE1/AE3+5D3) + OPAL780. For the PD-1/PD-L1 6-color panel, the combinations were: anti-CD8 (clone C8/144b) + OPAL620; anti-CD3 (clone PS1) + OPAL480; anti-CD68 (clone SP251) + OPAL520; anti-PD-L1 (clone SP142) + OPAL570; anti-PD-1 (clone NAT105, Cell Marque) + OPAL690; and anti-pan-cytokeratin (clone AE1/AE3+5D3) + OPAL780. mcIF staining methods for the OOU cohort were reported in Laumont et al.(6)

Panels A-D were imaged using the Vectra 3 multispectral imaging system (Akoya Biosciences). COUER panels were imaged using the motif mode of the Vectra Polaris multispectral imaging system (Akoya Biosciences); 20X fields of view were captured from the motif whole-slide scan and converted to component TIFF files using inForm image analysis software (Akoya Biosciences) for input into QuPath.(13)

For mcIHC (panels A and B), automated cell scoring, including the segregation of epithelial and stromal regions, was performed using inForm (Perkin Elmer). Epithelial regions were detected directly based on pan-cytokeratin positivity and cell morphology. Epithelium-negative, cellular (i.e., non-necrotic) tumor regions were defined as stroma. Acellular regions were defined as "other". After epithelium/stroma/other segmentation was completed, cell types and phenotypes of interest were identified, and examples of each were used to train the software to classify the remaining cells. The entire training procedure was performed five times to build consensus classifications of tissue proportions and cell counts for each core. Images and cell counts were manually inspected, and cores with discernible errors from automated scoring were corrected based on image review. Immune cells were quantified separately according to their intra-epithelial versus intra-stromal location.

mcIF images (panels C-D) were scored using QuPath software (v 0.2m2).(13) Similar to the approach used for mcIHC, tumor epithelium was segmented based on the intensity of pan-cytokeratin staining, and remaining regions were defined as stroma or "other". Segmentation results were manually inspected, and any poorly segmented images were re-processed using

different pan-cytokeratin thresholds or manual adjustment of segmentation masks to optimally separate epithelium from stroma. Cells were detected using the Watershed cell detection algorithm implemented in QuPath, and classifiers were trained to quantify cell populations of interest based on cell features using the random trees classifier. Classifications were visually inspected and revised as needed. For panel C, CD25 was not quantified due to weak and unreliable staining patterns; therefore, presumptive regulatory T cells (Tregs) were defined as CD8- FoxP3+ cells.(14) Images that could not be reliably segmented or analyzed due to other staining artefacts, tissue folds, or insufficient tissue area (<25% tissue or <5% tumor epithelium in the field of view) were removed from analysis.

As mentioned above, across all MOCOG participants (n=1,298), 94.2% of samples were successfully stained and scored for at least one panel. For the individual panels, 90.5% were successful for panel A, 89.3% for panel B, 87.9% for panel C, and 88.2% for panel D. Among participants who were successfully stained and scored for at least one panel (n=1,223), 80.5% were successful for all four panels. One study, MAY, was not stained with panel D due to lack of TMA availability; MAY was included in individual marker analyses but was excluded from multi-marker analyses. The COEUR cohort was scored using QuPath v0.3.0. Tissue segmentation was performed with the QuPath pixel classifier, and cell detection was performed using the QuPath implementation of StarDist.(15) Other scoring methods were the same as described for the MOCOG cohort. Scoring methods for the OOU cohort were reported in Laumont et al.(6) Additional image segmentation was performed using the QuPath pixel classifier to measure epithelial versus stromal content.

Statistics details

The epithelial content of each tumor sample was calculated as the average of the four ratios (from the four antibody staining panels) of the epithelial area to the sum of the epithelial area plus stromal area. These ratios were then dichotomized into epithelium-high and epithelium-

low based on the median values of the STS samples from the particular study. The median value for all samples was 66%, but this differed by study.

Pairwise Spearman correlations of the D values of the markers were calculated separately for each study and summarized as a weighted average (weighted by inverse variance after Fisher's z transformation of the correlation coefficients). Conditional logistic regression models were fit for LTS versus STS, MTS versus STS, and LTS versus MTS. Logistic regression analyses were carried out with $D^{0.25}$ using all the data. Logistic regression analyses were also carried out with the quartile values (scored as 1, 2, 3, 4). Logistic regression models were also fit separately for cases with epithelium-high and epithelium-low proportions. All logistic analyses were stratified by study.

Best subset variable selection (16) after forcing in intra-epithelial CD8+ T cells was used to determine the best set of immune cells to distinguish between the LTS and STS groups in the epithelium-high samples. Starting with single markers, additional markers associated with a statistically significant ($p \leq 0.05$) improvement in the Akaike Information Criteria were retained.(17)

Because survival times were available for COEUR and OOU cohorts, univariable Cox proportional hazards models were used to evaluate the association of each cell type's density (cells/mm^2, D; transformed as $D^{0.25}$) with overall survival. Models were fit across all patients in each study, as well as in epithelium-high and epithelium-low subgroups separately, with subgroups based on the median epithelial content in each study.

Molecular subtype (PrOTYPE) (1) data were available for a subset of the MOCOG patients (n=694). Analyses of the association between survival group and immune markers were conducted for each of the molecular subtypes using conditional logistic regression stratified by study.

SUPPLEMENTARY RESULTS

Complementary analyses using the COEUR and OOU HGSC cohorts.

Complementary analyses were performed using the Canadian COEUR cohort (n=981), which is a population-based cohort.(5) As with the MOCOG cohort, most TIL subsets in the COEUR cohort were associated with longer survival, including most phenotypic subsets of intra-epithelial T cells, B cells, PD-1+ cells, and PD-L1+ TAMs (**Supplementary Table 5**). Intra-stromal immune cells generally showed weaker associations with survival than intra-epithelial immune cells. Also as seen with the MOCOG cohort, the prognostic associations of most immune-cell subsets were stronger in epithelium-high cases (**Supplementary Table 5**). This was not attributable to increased immune cell densities, as epithelium-low cases generally had equal or higher densities of immune-cell subsets compared to epithelium-high cases (**Supplementary Table 6**), as seen in the MOCOG cohort.

We also evaluated HGSC tumors from a third group of patients (OOU; n=192) which had been stained with several T cell-relevant phenotypic markers (6) that were not included in the MOCOG or COEUR immunostaining panels. In particular, we recently showed that intra-epithelial CD4 and CD8 T cells co-expressing CD39, CD103 and PD-1 (so-called ‘triple positive’ TILs) have a substantially stronger prognostic effect than those expressing these markers singly or in pairwise combinations.(6) Consistent with the MOCOG and COEUR results, immune-cell subsets showed significant positive prognostic associations in epithelium-high but not epithelium-low cases (**Supplementary Table 7**), despite showing similar or higher densities in epithelium-low cases (**Supplementary Table 8**). Intra-epithelial 'triple-positive' CD8 TILs were strongly prognostic in the epithelium-high group (p=0.006) but lacked prognostic significance in the epithelium-low group (p=0.16) despite the density of these cells being no higher than in epithelium-high cases compared to epithelium-low cases. Thus, the prognostic influence of tumor epithelium extends to even the most prognostically significant phenotypic subset of T cells.

References for Supplementary Materials

1. Talhouk A, George J, Wang C, Budden T, Tan TZ, Chiu DS, et al. Development and validation of the Gene Expression Predictor of High-grade Serous Ovarian Carcinoma Molecular SubTYPE (PrOTYPE). *Clin Cancer Res.* 2020;26(20):5411-23.
2. Millstein J, Budden T, Goode EL, Anglesio MS, Talhouk A, Intermaggio MP, et al. Prognostic gene expression signature for high-grade serous ovarian cancer. *Ann Oncol.* 2020;31(9):1240-50.
3. Kobel M, Kang EY, Weir A, Rambau PF, Lee CH, Nelson GS, et al. p53 and ovarian carcinoma survival: an Ovarian Tumor Tissue Analysis consortium study. *J Pathol Clin Res.* 2023;9(3):208-22.
4. Emmanuel C, Chiew YE, George J, Etemadmoghadam D, Anglesio MS, Sharma R, et al. Genomic classification of serous ovarian cancer with adjacent borderline differentiates RAS pathway and TP53-mutant tumors and identifies NRAS as an oncogenic driver. *Clin Cancer Res.* 2014;20(24):6618-30.
5. Le Page C, Rahimi K, Kobel M, Tonin PN, Meunier L, Portelance L, et al. Characteristics and outcome of the COEUR Canadian validation cohort for ovarian cancer biomarkers. *BMC Cancer.* 2018;18(1):347.
6. Laumont CM, Wouters MCA, Smazynski J, Gierc NS, Chavez EA, Chong LC, et al. Single-cell profiles and prognostic impact of tumor-infiltrating lymphocytes coexpressing CD39, CD103, and PD-1 in ovarian cancer. *Clin Cancer Res.* 2021;27(14):4089-100.
7. Milne K, Kobel M, Kalloger SE, Barnes RO, Gao D, Gilks CB, et al. Systematic analysis of immune infiltrates in high-grade serous ovarian cancer reveals CD20, FoxP3 and TIA-1 as positive prognostic factors. *PLoS One.* 2009;4(7):e6412.
8. Nielsen JS, Sahota RA, Milne K, Kost SE, Nesslinger NJ, Watson PH, et al. CD20+ tumor-infiltrating lymphocytes have an atypical CD27- memory phenotype and together

with CD8+ T cells promote favorable prognosis in ovarian cancer. *Clin Cancer Res.* 2012;18(12):3281-92.

9. Webb JR, Milne K, Kroeger DR, and Nelson BH. PD-L1 expression is associated with tumor-infiltrating T cells and favorable prognosis in high-grade serous ovarian cancer. *Gynecol Oncol.* 2016;141(2):293-302.

10. Webb JR, Milne K, and Nelson BH. PD-1 and CD103 are widely coexpressed on prognostically favorable intraepithelial CD8 T cells in human ovarian cancer. *Cancer Immunol Res.* 2015;3(8):926-35.

11. Webb JR, Milne K, Watson P, Deleeuw RJ, and Nelson BH. Tumor-infiltrating lymphocytes expressing the tissue resident memory marker CD103 are associated with increased survival in high-grade serous ovarian cancer. *Clin Cancer Res.* 2014;20(2):434-44.

12. Pirici D, Mogoanta L, Kumar-Singh S, Pirici I, Margaritescu C, Simionescu C, et al. Antibody elution method for multiple immunohistochemistry on primary antibodies raised in the same species and of the same subtype. *J Histochem Cytochem.* 2009;57(6):567-75.

13. Bankhead P, Loughrey MB, Fernandez JA, Dombrowski Y, McArt DG, Dunne PD, et al. QuPath: Open source software for digital pathology image analysis. *Sci Rep.* 2017;7(1):16878.

14. deLeeuw RJ, Kroeger DR, Kost SE, Chang PP, Webb JR, and Nelson BH. CD25 identifies a subset of CD4(+)FoxP3(-) TIL that are exhausted yet prognostically favorable in human ovarian cancer. *Cancer Immunol Res.* 2015;3(3):245-53.

15. Weigert M, Schmidt U, Haase R, Sugawara K, and Myers G. *The IEEE Winter Conference on Applications of Computer Vision (WACV)*. Snowmass Village, Colorado; 2020.

16. Furnival GM, and Wilson RW. Regression by leaps and bounds. *Technometrics.* 1974;16:499-511.

17. Akaike H. A new look at the statistical model identification. *IEEE Transactions on Automatic Control.* 1974;19:716-23.

Supplementary Table 1. Included studies and number of samples that were stained and scored successfully.

Site	Name	Location	Years	Ascertainment of patients and clinical data	Pathology data and review	Ethics committee	Informed consent	Number scored
AOC	Australian Ovarian Cancer Study	Australia	2002-2006	Treatment centers throughout Australia; cancer registries serving Queensland, South and West Australia; regular follow- up by medical record review	Central review of pathology reports and histological slides by study pathologist	Peter MacCallum Cancer Centre Human Research Ethics Committee	Yes	311
DOV	Diseases of the Ovary and their Evaluation	US	2002-2009	13 counties from western Washington SEER registry	Central review of pathology reports and histological slides by study pathologist	Fred Hutchinson Cancer Research Center Institutional Review Board	Yes	205
MAY	Mayo Clinic Ovarian Cancer Study	US	2000-2013	Mayo Clinic medical records and death certificates	Central review of pathology reports and histological slides by study pathologist	Institutional Review Board of Mayo Clinic	Yes	172
VAN	Vancouver Ovarian Cancer Study	Canada	1982-present	Vancouver General, UBC, and BC Cancer Hospitals with outcome data provided the British Columbia cancer registry and the Cheryl Brown Outcomes Unit	Central review of pathology reports and histological slides by study pathologist	University of British Columbia - British Columbia Cancer Agency Research Ethics Board	Yes	121

Site	Name	Location	Years	Ascertainment of patients and clinical data	Pathology data and review	Ethics committee	Informed consent	Number scored
SEA	Study of Epidemiology and Risk Factors in Cancer Heredity	UK	1998-present	Eastern Region Cancer Intelligence Unit, West Midlands Cancer Intelligence Unit, and multiple cancer networks	Central review of pathology reports and histological slides by study pathologist	Cambridgeshire 4 Research Ethics Committee	Yes	87
WMH	WestMead Hospital	Australia	1992-present	The Crown Princess Mary Cancer Centre and affiliated hospitals	Review of pathology reports and histological slides by panel of gynecologic pathologists	Western Sydney Local Health District, Human Research Ethics Committee	Yes	60
NEC	New England Case Control Study	US	1992- 2008	Hospital tumor boards and cancer registries; clinical data from medical records	Central review of pathology reports and histological slides by study pathologist	Mass General Brigham Institutional Review Board	Yes	58
LAX	Women's Cancer Research Program - Cedars-Sinai Medical Center	US	1989-present	Women's Cancer Program Biorepository	Central review of pathology reports and histological slides by study pathologist	Institutional Review Board 3 of Cedars-Sinai Medical Center	Yes	57
HAW	Hawaii Ovarian Cancer Study	US	1993-2008	Hawaii Tumor Registry and medical records	Central review of pathology reports and histological slides by study pathologist	University of Hawaii, Committee on Human Studies	Yes	39

Site	Name	Location	Years	Ascertainment of patients and clinical data	Pathology data and review	Ethics committee	Informed consent	Number scored
GER	Germany Ovarian Cancer Study	Germany	1993-1996	Population-based study involving local and regional hospitals in Rhein-Neckar-Odenwald and Freiburg.	Central review of pathology reports and histological slides by study pathologist	Ethics Committee of the Heidelberg University Clinic	Yes	19
BAV	Bavarian Ovarian Cancer Study	Germany	2002-2006	Gynaecologic Oncology Center at the Comprehensive Cancer Center Erlangen-Nuremberg	Central review of pathology reports and histological slides by study pathologist	Ethics Committee of the Friedrich-Alexander-University Erlangen-Nuremberg	Yes	17
BRZ	Brazil Gynecologic Tumor Bank (BRZ) Study	Brazil	1987-2010	University Hospital of Ribeirao Preto School of Medicine, case series with prospective follow up	Pathology reports and histologic slides reviewed by gynecologic pathologists	Research Ethics Committee of Hospital das Clínicas of the Ribeirão Preto Medical School	No: pathology material	16
CAL	Calgary Serous Carcinoma Study	Canada	2003-2007	Hospital based retrospective observational study	Central review of pathology reports and histological slides by study pathologist	Conjoint Health Research Ethics Board	Yes	12
HOP	Hormones and Ovarian Cancer PrEdiction	US	2003-2009	Hospital registries and active surveillance of medical practices in PA, OH, and NY	Pathology information through medical chart review	University of Pittsburgh Insititutional Review Board and Roswell Park Cancer Institute Insititutional Review Board	Yes	9
TVA	OVAL BC	Canada	2004-2012	Alberta Cancer Registry and Provincial Cancer Treatment Centers	IHC supported slide review by gynecological pathologists	Health Research Ethics Board of Alberta	Yes, some cases No: pathology material	9

Site	Name	Location	Years	Ascertainment of patients and clinical data	Pathology data and review	Ethics committee	Informed consent	Number scored
UKO	United Kingdom Ovarian Cancer Population study	UK	2006-2010	Pathologist-reviewed cases from ten major Gynecologic Oncology NHS centres in England, Wales and Northern Ireland; NHS cancer and death registries	Central review of pathology reports by gynaecologic oncologist	NHS Central Office for Research Ethics Committees and University College London Committee on the Ethics of Human Research	Yes	9
CNI	CNIO Ovarian Cancer Study	Spain	2006-2013	Hospitals in Madrid in Medical Oncology Divisions	Pathology information was obtained through medical chart review in the Medical Oncology units	Bioethics and Animal Welfare Committee of the Carlos III Health Institute	Yes	8
AOV	Alberta Ovarian Tumor Types Study	Canada	1978-2010	Population-based Alberta Cancer Registry; periodic updates are performed for vital statistics	Pathology reports and histological slides review by the study pathologist	Alberta Health Services, Research Ethics	No: pathology material	7
TUE	Tuebingen University Women's Hospital (TUE) study	Germany	1999-2008	Department of Obstetrics and Gynaecology, Eberhard Karls Universitats Tübingen, Tübingen Germany	Pathology reports and histologic slides reviewed by gynecologic pathologist	Ethics-Committee at the University Hospital of Tübingen	Yes	7
TOTAL								1223

Supplementary Table 2. Sensitivity analysis of the $D^{0.25}$ odds ratios (ORs) of immune-cell subsets for long-term survivors (LTS) compared to short-term survivors (STS) for all participants and those who were known to have primary cytoreductive surgery (PCS).

			LTS compared to STS (n=790)					LTS compared to STS PCS only (n=454)				
Marker	Area	Cell type	OR	95% CI			p	OR	95% CI			p
CD8+FoxP3+-	Epithelial	CD8+ T cell	1.24	1.10	-	1.40	**<0.001**	1.30	1.10	-	1.53	**0.002**
CD8+FoxP3+-	Stromal	CD8+ T cell	1.12	1.02	-	1.23	**0.022**	1.12	0.98	-	1.27	0.094
CD8+FoxP3-	Epithelial	CD8+FoxP3- T cell	1.24	1.10	-	1.40	**<0.001**	1.30	1.10	-	1.53	**0.002**
CD8+FoxP3-	Stromal	CD8+FoxP3- T cell	1.12	1.01	-	1.23	**0.024**	1.11	0.98	-	1.27	0.10
CD8+FoxP3+	Epithelial	CD8+FoxP3+ T cell	1.40	1.14	-	1.72	**0.001**	1.65	1.24	-	2.20	**0.001**
CD8+FoxP3+	Stromal	CD8+FoxP3+ T cell	1.25	1.08	-	1.43	**0.002**	1.30	1.08	-	1.56	**0.006**
CD3+CD8-	Epithelial	CD4+ T cell	1.21	1.05	-	1.39	**0.007**	1.29	1.07	-	1.55	**0.008**
CD3+CD8-	Stromal	CD4+ T cell	1.13	1.01	-	1.25	**0.028**	1.09	0.95	-	1.26	0.22
CD8-FoxP3+	Epithelial	Presumptive Treg cell	1.11	0.96	-	1.29	0.15	1.09	0.90	-	1.33	0.37
CD8-FoxP3+	Stromal	Presumptive Treg cell	1.17	1.05	-	1.30	**0.004**	1.21	1.05	-	1.39	**0.007**
CD20+CD79+	Epithelial	B cell	1.27	1.09	-	1.48	**0.002**	1.37	1.11	-	1.68	**0.003**
CD20+CD79+	Stromal	B cell	1.07	0.97	-	1.18	0.20	1.08	0.95	-	1.24	0.25
CD20-CD79+	Epithelial	Plasma cell	1.22	1.05	-	1.41	**0.008**	1.31	1.08	-	1.59	**0.007**
CD20-CD79+	Stromal	Plasma cell	1.15	1.06	-	1.24	**0.001**	1.15	1.04	-	1.29	**0.009**
PD-1+	Epithelial	PD-1+ immune cell	1.33	1.17	-	1.51	**<0.001**	1.36	1.15	-	1.61	**<0.001**
PD-1+	Stromal	PD-1+ immune cell	1.18	1.07	-	1.31	**0.001**	1.13	0.99	-	1.30	0.077
CD68+PD-L1+	Epithelial	CD68+PD-L1+ TAM cell	1.15	1.00	-	1.31	**0.043**	1.16	0.97	-	1.38	0.099
CD68+PD-L1+	Stromal	CD68+PD-L1+ TAM cell	1.10	1.00	-	1.22	0.053	1.15	1.01	-	1.32	**0.039**
CD68+PD-L1-	Epithelial	CD68+PD-L1- TAM cell	0.93	0.78	-	1.11	0.44	1.02	0.79	-	1.32	0.87
CD68+PD-L1-	Stromal	CD68+PD-L1- TAM cell	0.94	0.82	-	1.07	0.36	0.95	0.80	-	1.14	0.61
CD68-PD-L1+	Epithelial	CD68-PD-L1+ cell	1.22	1.07	-	1.38	**0.002**	1.22	1.03	-	1.46	**0.025**
CD68-PD-L1+	Stromal	CD68-PD-L1+ cell	1.18	1.07	-	1.31	**0.001**	1.19	1.04	-	1.37	**0.014**

Supplementary Table 3. Quartile odds ratios (ORs) of immune-cell subsets comparing long-term survivors (LTS) to short-term survivors (STS) by epithelium group.[A]

			Epithelium-low (n=295)			Epithelium-high (n=304)		
Marker	Area	Cell type	OR	95% CI	p	OR	95% CI	p
CD8+FoxP3+-	Epithelial	CD8+ T cell	1.25	1.00 - 1.55	**0.047**	1.54	1.25 - 1.89	**<0.001**
CD8+FoxP3+-	Stromal	CD8+ T cell	1.11	0.90 - 1.38	0.32	1.44	1.17 - 1.78	**0.001**
CD8+FoxP3-	Epithelial	CD8+FoxP3- T cell	1.20	0.97 - 1.48	0.092	1.51	1.22 - 1.86	**<0.001**
CD8+FoxP3-	Stromal	CD8+FoxP3- T cell	1.13	0.91 - 1.39	0.26	1.44	1.17 - 1.78	**0.001**
CD8+FoxP3+	Epithelial	CD8+FoxP3+ T cell	1.38	0.96 - 1.97	0.078	1.58	1.13 - 2.22	**0.007**
CD8+FoxP3+	Stromal	CD8+FoxP3+ T cell	1.13	0.78 - 1.64	0.53	1.83	1.26 - 2.66	**0.001**
CD3+CD8-	Epithelial	CD4+ T cell	1.10	0.91 - 1.34	0.33	1.35	1.10 - 1.67	**0.004**
CD3+CD8-	Stromal	CD4+ T cell	0.97	0.79 - 1.19	0.76	1.34	1.10 - 1.63	**0.004**
CD8-FoxP3+	Epithelial	Presumptive Treg cell	1.04	0.85 - 1.28	0.70	1.23	1.01 - 1.49	**0.037**
CD8-FoxP3+	Stromal	Presumptive Treg cell	1.04	0.83 - 1.30	0.71	1.31	1.08 - 1.59	**0.006**
CD20+CD79+[B]	Epithelial	B cell	1.24	0.75 - 2.04	0.41	2.28	1.38 - 3.78	**0.001**
CD20+CD79+[B]	Stromal	B cell	0.78	0.48 - 1.27	0.32	2.20	1.36 - 3.57	**0.001**
CD20-CD79+[B]	Epithelial	Plasma cell	1.18	0.70 - 2.01	0.53	2.25	1.35 - 3.75	**0.002**
CD20-CD79+[B]	Stromal	Plasma cell	0.91	0.54 - 1.55	0.73	1.92	1.20 - 3.08	**0.007**
PD-1+	Epithelial	PD-1+ immune cell	1.32	1.04 - 1.68	**0.023**	1.43	1.14 - 1.79	**0.002**
PD-1+	Stromal	PD-1+ immune cell	1.14	0.90 - 1.45	0.28	1.33	1.07 - 1.65	**0.011**
CD68+PD-L1+	Epithelial	CD68+PD-L1+ TAM cell	1.06	0.86 - 1.30	0.59	1.10	0.90 - 1.33	0.36
CD68+PD-L1+	Stromal	CD68+PD-L1+ TAM cell	0.97	0.79 - 1.20	0.81	1.15	0.95 - 1.39	0.15
CD68+PD-L1-	Epithelial	CD68+PD-L1- TAM cell	0.88	0.69 - 1.12	0.30	0.93	0.74 - 1.15	0.49
CD68+PD-L1-	Stromal	CD68+PD-L1- TAM cell	0.94	0.74 - 1.20	0.64	0.98	0.79 - 1.22	0.87
CD68-PD-L1+	Epithelial	CD68-PD-L1+ cell	1.10	0.89 - 1.34	0.38	1.17	0.96 - 1.41	0.11
CD68-PD-L1+	Stromal	CD68-PD-L1+ cell	1.10	0.90 - 1.35	0.35	1.24	1.02 - 1.50	**0.028**

[A]Based on the five largest studies. [B]Coded binary: zero/non zero.

Supplementary Table 4. $D^{0.25}$ odds ratios (ORs) of immune-cell subsets comparing medium-term survivors (MTS) to short-term survivors (STS) by epithelium group.

			Epithelium-low (n=429)					Epithelium-high (n=420)				
Marker	Area	Cell type	OR	95% CI			p	OR	95% CI			p
CD8+FoxP3+-	Epithelial	CD8+ T cell	1.07	0.93	-	1.24	0.35	1.10	0.93	-	1.29	0.27
CD8+FoxP3+-	Stromal	CD8+ T cell	0.96	0.83	-	1.11	0.56	1.02	0.90	-	1.15	0.80
CD8+FoxP3-	Epithelial	CD8+FoxP3- T cell	1.07	0.93	-	1.24	0.36	1.10	0.93	-	1.29	0.28
CD8+FoxP3-	Stromal	CD8+FoxP3- T cell	0.96	0.83	-	1.11	0.56	1.01	0.89	-	1.15	0.84
CD8+FoxP3+	Epithelial	CD8+FoxP3+ T cell	1.10	0.83	-	1.45	0.51	1.21	0.91	-	1.61	0.20
CD8+FoxP3+	Stromal	CD8+FoxP3+ T cell	0.92	0.76	-	1.12	0.41	1.26	1.04	-	1.53	**0.017**
CD3+CD8-	Epithelial	CD4+ T cell	1.15	0.95	-	1.37	0.14	1.24	1.01	-	1.51	**0.038**
CD3+CD8-	Stromal	CD4+ T cell	1.00	0.86	-	1.17	0.97	1.12	0.97	-	1.30	0.12
CD8-FoxP3+	Epithelial	Presumptive Treg cell	1.08	0.88	-	1.33	0.46	1.09	0.89	-	1.34	0.40
CD8-FoxP3+	Stromal	Presumptive Treg cell	0.94	0.78	-	1.13	0.51	1.10	0.96	-	1.25	0.16
CD20+CD79+	Epithelial	B cell	1.12	0.92	-	1.36	0.28	1.36	1.06	-	1.74	**0.014**
CD20+CD79+	Stromal	B cell	0.90	0.78	-	1.04	0.15	1.20	1.03	-	1.39	**0.018**
CD20-CD79+	Epithelial	Plasma cell	1.08	0.88	-	1.32	0.49	1.25	1.01	-	1.54	**0.040**
CD20-CD79+	Stromal	Plasma cell	0.97	0.86	-	1.10	0.65	1.13	1.01	-	1.26	**0.034**
PD-1+	Epithelial	PD-1+ immune cell	1.14	0.95	-	1.37	0.16	1.21	1.01	-	1.44	**0.035**
PD-1+	Stromal	PD-1+ immune cell	1.04	0.88	-	1.23	0.66	1.05	0.93	-	1.20	0.43
CD68+PD-L1+	Epithelial	CD68+PD-L1+ TAM cell	1.11	0.92	-	1.33	0.28	1.12	0.93	-	1.35	0.25
CD68+PD-L1+	Stromal	CD68+PD-L1+ TAM cell	0.97	0.83	-	1.14	0.74	1.07	0.94	-	1.22	0.31
CD68+PD-L1-	Epithelial	CD68+PD-L1- TAM cell	1.11	0.89	-	1.39	0.37	1.04	0.81	-	1.35	0.74
CD68+PD-L1-	Stromal	CD68+PD-L1- TAM cell	1.01	0.82	-	1.23	0.96	0.94	0.80	-	1.10	0.44
CD68-PD-L1+	Epithelial	CD68-PD-L1+ cell	1.01	0.86	-	1.19	0.87	1.15	0.96	-	1.37	0.14
CD68-PD-L1+	Stromal	CD68-PD-L1+ cell	0.94	0.81	-	1.09	0.41	1.14	0.99	-	1.31	0.063

Supplementary Table 5. $D^{0.25}$ hazard ratios (HRs) of immune-cell subsets for overall survival (OS) in the COEUR cohort.

			Overall (n=981)			Epithelium-low (n=491)			Epithelium-high (n=490)		
Marker	Area	Cell type	HR	95% CI	p	HR	95% CI	p	HR	95% CI	p
CD3+CD8+PD-1-	Epithelial	PD-1- CD8+ T cell	1.10	1.03 - 1.18	**0.004**	1.11	1.01 - 1.21	**0.022**	1.12	1.01 - 1.24	**0.028**
CD3+CD8+PD-1-	Stromal	PD-1- CD8+ T cell	1.03	0.97 - 1.08	0.33	1.05	0.97 - 1.13	0.26	1.03	0.96 - 1.11	0.37
CD3+CD8+PD-1+	Epithelial	PD-1+ CD8+ T cell	1.09	1.02 - 1.17	**0.009**	1.08	1.00 - 1.18	0.065	1.13	1.02 - 1.26	**0.019**
CD3+CD8+PD-1+	Stromal	PD-1+ CD8+ T cell	1.05	1.00 - 1.11	**0.049**	1.07	0.99 - 1.16	0.085	1.07	0.99 - 1.15	0.079
CD3+CD8+FoxP3+	Epithelial	CD8+ FoxP3+ T cell	1.22	1.07 - 1.40	**0.003**	1.09	0.92 - 1.30	0.30	1.40	1.14 - 1.73	**0.002**
CD3+CD8+FoxP3+	Stromal	CD8+ FoxP3+ T cell	1.06	0.94 - 1.20	0.31	1.13	0.97 - 1.32	0.11	1.05	0.86 - 1.27	0.66
CD3+CD8-	Epithelial	CD4+ T cell	1.10	1.01 - 1.19	**0.023**	1.05	0.95 - 1.16	0.35	1.19	1.05 - 1.34	**0.007**
CD3+CD8-	Stromal	CD4+ T cell	1.08	1.01 - 1.14	**0.021**	1.07	0.98 - 1.16	0.15	1.13	1.03 - 1.24	**0.008**
CD3+CD8-PD-1-	Epithelial	PD-1- CD4+ T cell	1.08	1.00 - 1.17	**0.040**	1.05	0.95 - 1.16	0.38	1.15	1.02 - 1.29	**0.020**
CD3+CD8-PD-1-	Stromal	PD-1- CD4+ T cell	1.05	0.99 - 1.11	0.091	1.07	0.99 - 1.17	0.083	1.04	0.97 - 1.13	0.28
CD3+CD8-PD-1+	Epithelial	PD-1+ CD4+ T cell	1.15	1.07 - 1.24	**<0.001**	1.12	1.02 - 1.24	**0.024**	1.20	1.07 - 1.35	**0.002**
CD3+CD8-PD-1+	Stromal	PD-1+ CD4+ T cell	1.09	1.03 - 1.15	**0.003**	1.14	1.04 - 1.24	**0.003**	1.08	1.00 - 1.17	**0.037**
CD3+CD8-FoxP3+	Epithelial	Presumptive Treg	1.11	1.03 - 1.20	**0.006**	1.08	0.98 - 1.20	0.11	1.16	1.04 - 1.30	**0.009**
CD3+CD8-FoxP3+	Stromal	Presumptive Treg	1.06	0.99 - 1.13	0.082	1.02	0.92 - 1.12	0.74	1.14	1.04 - 1.25	**0.007**
CD68-PDL1-PD-1+	Epithelial	PD-1+ lymphocyte	1.08	1.00 - 1.17	**0.045**	1.06	0.96 - 1.17	0.27	1.14	1.01 - 1.29	**0.040**
CD68-PDL1-PD-1+	Stromal	PD-1+ lymphocyte	1.09	1.03 - 1.16	**0.004**	1.19	1.08 - 1.32	**<0.001**	1.08	0.99 - 1.18	0.078
CD20+	Epithelial	B cell	1.15	1.05 - 1.27	**0.003**	1.13	1.00 - 1.27	0.048	1.23	1.04 - 1.44	**0.013**
CD20+	Stromal	B cell	1.08	1.02 - 1.14	**0.009**	1.10	1.02 - 1.18	**0.010**	1.11	1.00 - 1.22	**0.043**
CD79A+CD20-	Epithelial	Plasma cell	1.07	0.98 - 1.16	0.13	1.13	1.01 - 1.26	**0.031**	1.01	0.88 - 1.16	0.89
CD79A+CD20-	Stromal	Plasma cell	1.03	0.99 - 1.08	0.13	1.09	1.02 - 1.16	**0.007**	1.01	0.94 - 1.08	0.82
CD68+PD-L1-	Epithelial	PD-L1- TAM cell	0.99	0.89 - 1.09	0.79	0.89	0.77 - 1.04	0.14	1.11	0.96 - 1.29	0.17
CD68+PD-L1-	Stromal	PD-L1- TAM cell	0.95	0.87 - 1.03	0.20	0.99	0.85 - 1.15	0.86	0.94	0.85 - 1.05	0.27
CD68+PD-L1+	Epithelial	PD-L1+ TAM cell	1.11	1.04 - 1.18	**0.002**	1.05	0.97 - 1.14	0.20	1.18	1.06 - 1.32	**0.002**
CD68+PD-L1+	Stromal	PD-L1+ TAM cell	1.10	1.04 - 1.15	**<0.001**	1.08	1.00 - 1.16	**0.045**	1.09	1.02 - 1.17	**0.009**
PD-L1+CK+	Epithelial	PD-L1+ cell	1.01	0.90 - 1.13	0.87	1.03	0.88 - 1.20	0.73	0.97	0.82 - 1.15	0.74

Supplementary Table 6. Distribution of immune-cell densities, overall and by epithelial content in the COEUR cohort.

			Overall (n=981)			Epithelium-low (n=491)			Epithelium-high (n=490)		
Marker	Area	Cell type	Median	Q1[A]	Q3[A]	Median	Q1	Q3	Median	Q1	Q3
CD3+CD8+PD-1-	Epithelium	PD-1- CD8+ T cell	24.2	6.1	82.3	30.5	8.14	109	20	4.17	58.7
CD3+CD8+PD-1-	Stroma	PD-1- CD8+ T cell	84.6	21	267	105	27.6	312	64.4	14.7	220
CD3+CD8+PD-1+	Epithelium	PD-1+ CD8+ T cell	15.2	2.7	57.9	21.8	2.9	72.8	10.7	2.08	44.9
CD3+CD8+PD-1+	Stroma	PD-1+ CD8+ T cell	51.7	10.5	166	68.5	18.2	195	39.8	4.44	130
CD3+CD8+FoxP3+	Epithelium	CD8+ FoxP3+ T cell	0	0	1.5	0	0	0	0	0	1.5
CD3+CD8+FoxP3+	Stroma	CD8+ FoxP3+ T cell	0	0	0	0	0	2.9	0	0	0
CD3+CD8-	Epithelium	CD4+ T cell	12.9	3.0	38.4	18.3	3.08	47.1	10.7	3.0	29.4
CD3+CD8-	Stroma	CD4+ T cell	59.1	16.4	174	92.3	25.9	221	43.4	10.8	124
CD3+CD8-PD-1-	Epithelium	PD-1- CD4+ T cell	29.2	8.9	79.8	35.0	10.8	93.1	25.1	8.1	68.3
CD3+CD8-PD-1-	Stroma	PD-1- CD4+ T cell	193	61.6	432	210	74.2	447	172	54.9	416
CD3+CD8-PD-1+	Epithelium	PD-1+ CD4+ T cell	8.9	0	28.5	11.6	0	32.3	6.5	0	21.7
CD3+CD8-PD-1+	Stroma	PD-1+ CD4+ T cell	51.1	10.0	155	61.2	16.6	177	39.5	0	139
CD3+CD8-FoxP3+	Epithelium	Presumptive Treg	13.5	2.7	41.8	17.4	3.2	52.2	10.8	1.9	33.1
CD3+CD8-FoxP3+	Stroma	Presumptive Treg	31.1	6.4	85.9	42.0	10.4	103	20.7	3.4	66.1
CD68-PDL1-PD-1+	Epithelium	PD-1+ lymphocyte	17.3	4.6	50.4	23.3	6.11	70.4	14.2	4.3	37
CD68-PDL1-PD-1+	Stroma	PD-1+ lymphocyte	47.3	14.6	126	60.0	21.6	157	36.0	9.2	103
CD20+	Epithelium	B cell	0	0	3.6	0	0	5.9	0	0	3.1
CD20+	Stroma	B cell	4.1	0	30.2	6.7	0	48	0	0	16.6
CD79A+CD20-	Epithelium	Plasma cell	0	0	5.3	0	0	7.3	0	0	3.5
CD79A+CD20-	Stroma	Plasma cell	14.9	0	126	27.4	3.1	196	6.4	0	77.4
CD68+PD-L1-	Epithelium	PD-L1- TAM cell	215	124	352	240	142	386	200	109	331
CD68+PD-L1-	Stroma	PD-L1- TAM cell	356	213	604	376	233	600	331	177	611
CD68+PD-L1+	Epithelium	PD-L1+ TAM cell	52.3	14.5	146	51.3	10.4	164	52.8	18.6	141
CD68+PD-L1+	Stroma	PD-L1+ TAM cell	185	53.7	539	139	47.5	426	251	70.2	703
PDL1+CK+	Epithelium	PD-L1+ cell	0	0	0	0	0	0	0	0	1.4

[A]Q1, cutpoint between first and second quartiles; Q3, cutpoint between third and fourth quartiles.

Supplementary Table 7. $D^{0.25}$ hazard ratios (HRs) of intra-epithelial immune-cell subsets for overall survival (OS) in the OOU cohort.

	Overall (n=192)					Epithelium-low (n=96)					Epithelium-high n=96)				
Marker[A]	HR	95% CI			p	HR	95% CI			p	HR	95% CI			p
CD8+	1.23	0.99	-	1.52	0.061	1.06	0.79	-	1.42	0.69	1.47	1.06	-	2.04	**0.022**
CD103+	1.26	1.03	-	1.53	**0.022**	1.25	0.88	-	1.77	0.22	1.28	1.00	-	1.63	0.052
CD39+	1.22	1.02	-	1.47	**0.032**	1.07	0.80	-	1.42	0.67	1.42	1.09	-	1.85	**0.009**
CD103+PD-1+	1.28	1.08	-	1.52	**0.005**	1.22	0.97	-	1.54	0.095	1.36	1.04	-	1.78	**0.025**
CD39+PD-1+	1.25	1.00	-	1.56	0.052	0.99	0.72	-	1.37	0.96	1.53	1.11	-	2.11	**0.010**
CD39+CD103+PD-1+	1.46	1.14	-	1.86	**0.003**	1.32	0.95	-	1.83	0.10	1.67	1.12	-	2.48	**0.011**
CD8+CD39+CD103+PD-1+	1.41	1.12	-	1.76	**0.003**	1.25	0.92	-	1.69	0.16	1.62	1.15	-	2.29	**0.006**

[A]Not all markers define a specific cell type, therefore cell type names are not shown.

Supplementary Table 8. Distribution of intra-epithelial immune-cell densities, overall and by epithelial content in the OOU cohort.

	Overall (n=192)			Epithelium-low (n=96)			Epithelium-high (n=96)		
Marker[A]	Median	Q1[B]	Q3[B]	Median	Q1	Q3	Median	Q1	Q3
CD8+	6.6	0	17.7	10.6	2.5	24.8	4.5	0	11.9
CD103+	23.5	8.4	62.8	27.6	11.5	66.2	21.3	4.1	62.2
CD39+	91.7	30.2	223	89.0	33.7	223	93.2	21.2	222
CD103+PD-1+	7.6	0	32.4	9.7	0	55.9	4.4	0	22.2
CD39+PD-1+	2.9	0	11.0	2.8	0	13.4	3.4	0	8.7
CD39+CD103+PD-1+	0	0	6.7	0	0	8.6	0	0	4.7
CD8+CD39+CD103+PD-1+	2.0	0	8.5	2.3	0	9.0	1.9	0	8.4

[A]Not all markers define a specific cell type, therefore cell type names are not shown. [B]Q1, cutpoint between first and second quartiles; Q3, cut point between third and fourth quartiles.

Supplementary Table 9. $D^{0.25}$ odds ratios (ORs) of epithelium-high immune-cell densities comparing long-term survivors (LTS) to short-term survivors (STS) after fitting intra-epithelial CD8+ T cell and intra-stromal CD20+ B cell immune markers.

Marker	Area	Cell type	OR	95% CI			p
CD8+FoxP3+-	Stromal	CD8+ T cell	0.96	0.79	-	1.18	0.72
CD3+CD8-	Epithelial	CD4+ T cell	1.16	0.88	-	1.53	0.30
CD3+CD8-	Stromal	CD4+ T cell	1.09	0.91	-	1.31	0.34
CD8-FoxP3+	Epithelial	Presumptive Treg cell	1.09	0.79	-	1.50	0.61
CD8-FoxP3+	Stromal	Presumptive Treg cell	1.08	0.90	-	1.30	0.43
CD20+CD79+	Epithelial	B cell	1.18	0.82	-	1.70	0.36
CD20-CD79+	Epithelial	Plasma cell	1.10	0.81	-	1.47	0.55
CD20-CD79+	Stromal	Plasma cell	1.03	0.88	-	1.22	0.70
PD-1+	Epithelial	PD-1+ immune cell	1.23	0.95	-	1.60	0.12
PD-1+	Stromal	PD-1+ immune cell	1.05	0.89	-	1.24	0.58
CD68+PD-L1+	Epithelial	CD68+PD-L1+ TAM cell	1.02	0.82	-	1.26	0.88
CD68+PD-L1+	Stromal	CD68+PD-L1+ TAM cell	1.07	0.92	-	1.25	0.35
CD68+PD-L1-	Epithelial	CD68+PD-L1- TAM cell	1.03	0.78	-	1.38	0.82
CD68+PD-L1-	Stromal	CD68+PD-L1- TAM cell	0.97	0.81	-	1.16	0.75
CD68-PD-L1+	Epithelial	CD68-PD-L1+ cell	1.11	0.90	-	1.36	0.33
CD68-PD-L1+	Stromal	CD68-PD-L1+ cell	1.11	0.94	-	1.29	0.22

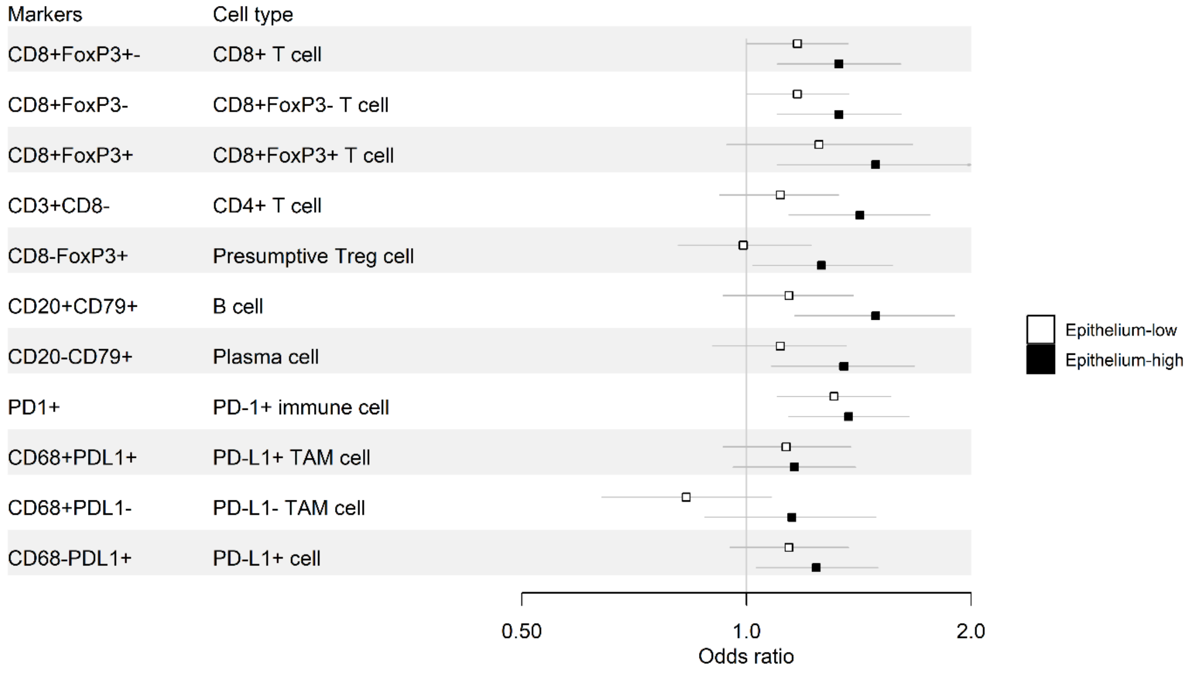

Supplementary Figure 1. Forest plot of the odds ratios and 95% confidence intervals of LTS compared to STS for intra-stromal immune-cell subsets stratified by epithelium-high versus epithelium-low tumors.

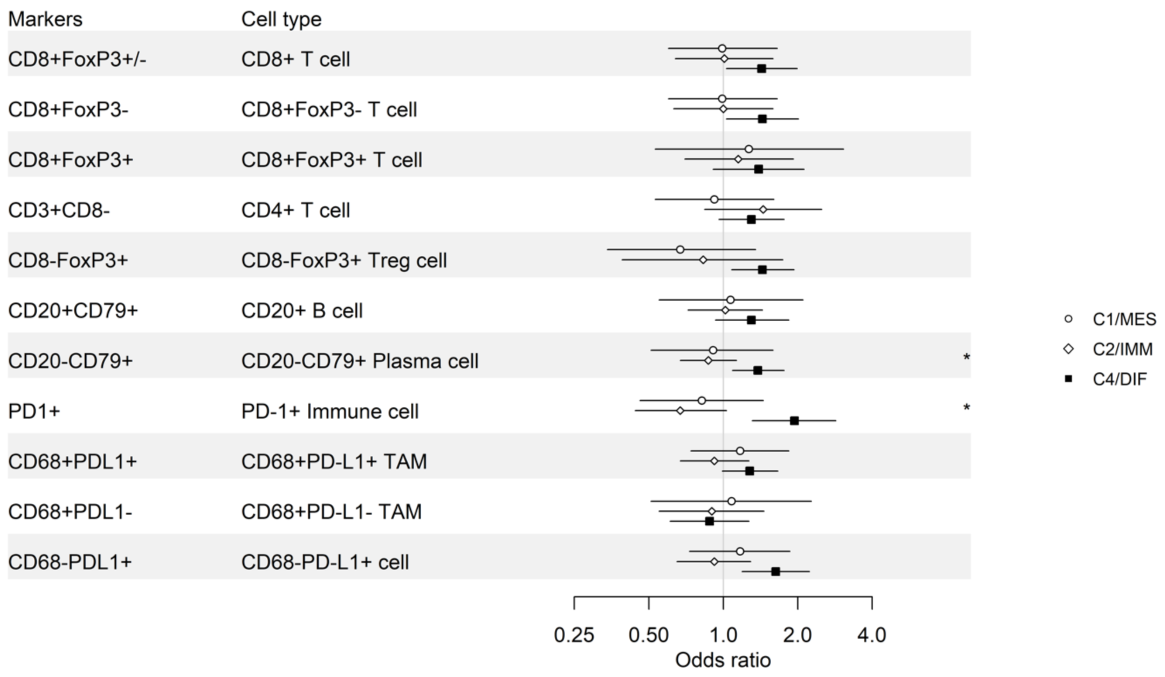

Supplementary Figure 2. Forest plot of the odds ratios and 95% confidence intervals of LTS compared to STS of intra-stromal immune-cell subsets for the C1/MES, C2/IMM and C4/DIF molecular subtypes in epithelium-high cases. The C5/PRO subtype is not presented as several could not be calculated. * indicates the p-value for heterogeneity across the subtypes is <0.05.

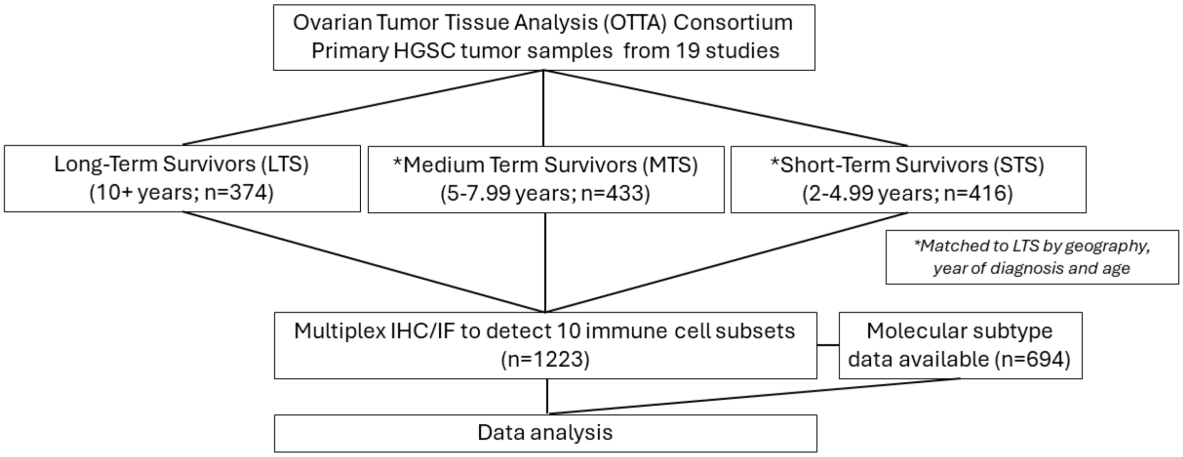

Supplementary Figure 3. Study design flow chart.
